# Dose-adjusted EPOCH chemotherapy with bortezomib and raltegravir for human T-cell leukemia virus-associated adult T-cell leukemia lymphoma

**DOI:** 10.1038/bcj.2016.21

**Published:** 2016-03-25

**Authors:** L Ratner, D Rauch, H Abel, B Caruso, A Noy, S K Barta, S Parekh, J C Ramos, R Ambinder, A Phillips, J Harding, H H Baydoun, X Cheng, S Jacobson

**Affiliations:** 1 Division of Oncology, Department of Medicine, Washington University School of Medicine, St Louis, MO, USA; 2 Department of Genetics, Washington University School of Medicine, St Louis, MO, USA; 3 Viral Immunology Section, Neuroimmunology and Neurovirology Division, NINDS, NIH, Bethesda, MD, USA; 4 Lymphoma Service, Memorial Sloan Kettering Cancer Center, New York, NY, USA; 5 Division of Hematology–Oncology, Department of Medicine, Montefiore Medical Center, Albert Einstein College of Medicine, Bronx, NY, USA; 6 Division of Hematology–Oncology, Department of Medicine, University of Miami School of Medicine, Miami, FL, USA; 7 Division of Hematologic Malignancies, Department of Oncology, Johns Hopkins School of Medicine, Baltimore, MD, USA; 8 Division of Hematology–Oncology, Department of Medicine, Columbia University Medical Center, New York, NY, USA

‘Acute' and ‘lymphoma' subtypes are the most common forms of human T-cell leukemia virus (HTLV)-associated adult T-cell leukemia lymphoma (ATLL), with median survivals of only 8–12 months.^1^ Treatment with interferon, zidovudine, arsenic, mogamulizumab, multiagent chemotherapy and allogeneic stem cell transplants yielded positive results, but most individuals with ATLL succumb to their disease. In our previous multicenter trial of 19 subjects, using dose-adjusted infusional chemotherapy (DA-EPOCH) followed by maintenance interferon and zidovudine, there were two complete remissions, four subjects with early severe toxicity and an overall response rate of 58%.^2^ Half of the evaluable subjects manifested significantly increased viral gene expression with disease progression and new integration sites were identified in most subjects. The current study was designed to test the tolerability and efficacy of a regimen with: (1) decreased dose intensity during the initial cycles of therapy, (2) addition of bortezomib to block NFκB activation and (3) incorporate the antiviral raltegravir (previously shown to block HTLV-1 replication) (Supplementary Protocol File).^3^ In addition, we assessed viral and cellular parameters of response.

Our phase 1/2 trial enrolled consenting subjects with untreated (*n*=14) or previously treated (*n*=4) acute (*n*=6) or lymphoma ATLL (*n*=12), adequate hematologic, renal and hepatic function, and Karnofsky performance score >50 (Figure 1a). Subjects were enrolled at six centers from 2011 to 2013 and were treated with DA-EPOCH with intravenous bortezomib (d1 and 4, 1.0 mg/m^2^), and daily raltegravir (400 mg bid) was initiated with cycle 2 therapy. Patients received up to six 21-day cycles of treatment unless they had evidence of disease progression or dose-limiting toxicity. Disease staging and viral studies were performed at baseline, just before cycles 3 and 5, and after completion of treatment. Viral DNA load, *tax* and *hbz* RNA levels were quantified by digital droplet polymerase chain reaction.^4^ Viral sequences were determined by Illumina HiSeq-2500 (San Diego, CA, USA). The sample size was based on a two-stage Simon's design for a response rate of at least 30%, with a significance level of 10% and a power of 80%. The Kaplan–Meier method was used to determine duration of response and survival. Correlations of viral parameters with response were assessed by two-sided Student's *t*-tests (see Supplementary Methods).

The mean age of subjects was 52 years and all but three US subjects were born in the Caribbean (Figure 1a). At baseline, all but one subject had stage IV disease, nine had hypercalcemia, 16 had elevated lactate dehydrogenase levels, one had thrombocytopenia and six patients had hypoalbuminemia. The mean absolute lymphocyte count was 38 900/mm^3^ for acute ATLL patients and 1610/mm^3^ for those with lymphoma. Patients received on average, 4.5 cycles of DA-EPOCH-bortezomib, with raltegravir. Complications of therapy were similar to those expected for DA-EPOCH alone (Figure 1b, Supplementary Table S1).

Three subjects achieved complete remission and eight subjects achieved partial remission, with similar response rates (67%) in acute and lymphoma ATLL (Figure 1a). With follow-up of >2 years for all subjects, median progression-free and overall survival were 5.8 and 6.2 mos, respectively, with four subjects still alive (Figure 1c). The responses in this study were similar to those of a previous DA-EPOCH trial without bortezomib and raltegravir, suggesting that NFκB target genes and virus replication were incompletely inhibited, or they did not contribute to chemotherapy resistance. In the current study, no patients had dose-limiting toxicity, likely due to the lower dose of cyclophosphamide at treatment initiation.^2^ Similar response rates were reported with other chemotherapy approaches.^5, 6^

Proviral loads at baseline were 0.368 copies/peripheral blood mononuclear cell (PBMC) for acute ATLL subjects and 0.216 copies/PBMC for lymphoma patients (*P*=0.002), but were similar for responders (mean 0.372) and nonresponders (mean 0.0417, *P*=0.99). However, proviral loads were lower at study completion for responders (mean 0.0128) compared with nonresponders (mean 0.033, *P*<0.0001).

Levels in PBMCs were determined for *tax* and *hbz* messenger RNAs, encoded from the plus and minus viral strands, respectively. Baseline *tax* messenger RNA levels for acute ATLL subjects were 0.04 copies, and for lymphoma ATLL, 2.0 copies/100 copies *hprt* messenger RNA (range 0–13.4, *P*=0.33). The mean *hbz* messenger RNA levels at baseline were 98.5 copies for acute ATLL and 8.7 copies/100 copies *hprt* messenger RNA for patients with lymphoma (*P*=0.016). There was no difference in baseline *hbz* RNA levels between responders (mean 37.0 copies) and nonresponders (mean 41.9 copies, *P*=0.11), but lower levels at study conclusion in responders (mean 7.33 copies) than nonresponders (mean 35.7 copies, *P*<0.0001).

HTLV-1 integrase sequences at baseline and at the end of the study exhibited <1% intra- and interpatient nucleotide divergence. Only three residues differed from the consensus HTLV-1 sequence, in agreement with the high levels of sequence conservation reported from this virus.^7^ Only one residue (E100K) exhibited an increased frequency at the end of the study (0.67) compared with the baseline (0.46), corresponding to a raltegravir-resistant mutation in HIV-1 (E92Q; http://hivdb.stanford.edu/DR/INIResiNote.html). Sequence analysis of integration sites revealed 1–5 clonal integration sites in each individual and no significant differences between baseline and end of the study samples. Thus, in contrast to our previous trial lacking antivirals during induction chemotherapy, the current study subjects exhibited little evidence of active virus replication.^2^

RNAseq analysis was performed on PBMC samples obtained at baseline and end point from subjects with acute ATLL including two responders and two nonresponders who had 66–99% CD4+ lymphocytes/PBMC at baseline. Effects on NFκB target genes are shown in Supplementary Figures S1 and 2. The most significant difference between these groups was the expression of the Src family tyrosine kinase Blk (Figure 2). This result was surprising since Blk is predominantly expressed in B cells. However, a subset of the 52 primary ATLL samples from a separate patient cohort also express elevated Blk (Supplementary Figure S3).^8^ Blk is constitutively active in cutaneous T-cell lymphoma^9^ and can be effectively targeted with dasatinib.^10^ Blk expression is inversely correlated with CD101 expression in most ATLL samples (Supplementary Figure S4). CD101 is a repressor of T cell receptor signaling and T-cell proliferation.^11^ This finding is consistent with the high rates of gain-of-function mutations in genes encoding T cell receptor-pathway proteins (unpublished data and Kataoka *et al.*^12^).

In conclusion, the current regimen was well-tolerated. Changes in proviral load and *hbz* RNA expression provide potential markers of antitumor response. Concurrent antiviral integrase inhibitor therapy was well-tolerated and limited virus replication. Repression of NFκB through proteasome inhibition targets a key pathway responsible for apoptosis resistance.^13^ Expression of Blk and reduction of CD101 in subjects that failed to respond to therapy suggests a mechanism and a therapeutic target for future trials. Allogeneic stem cell transplantation is an effective consolidation therapy for ATLL and was utilized in three patients on the current trial.^14^ Although response rates in the current trial were short-lived, this therapy could serve as a bridge to allogeneic stem cell transplantation to induce more long-lived responses. Future studies are focused on other immunotherapy approaches for this disease, such as therapeutic vaccines, CAR T cells or immune checkpoint therapies.^15^

## Figures and Tables

**Figure 1:**
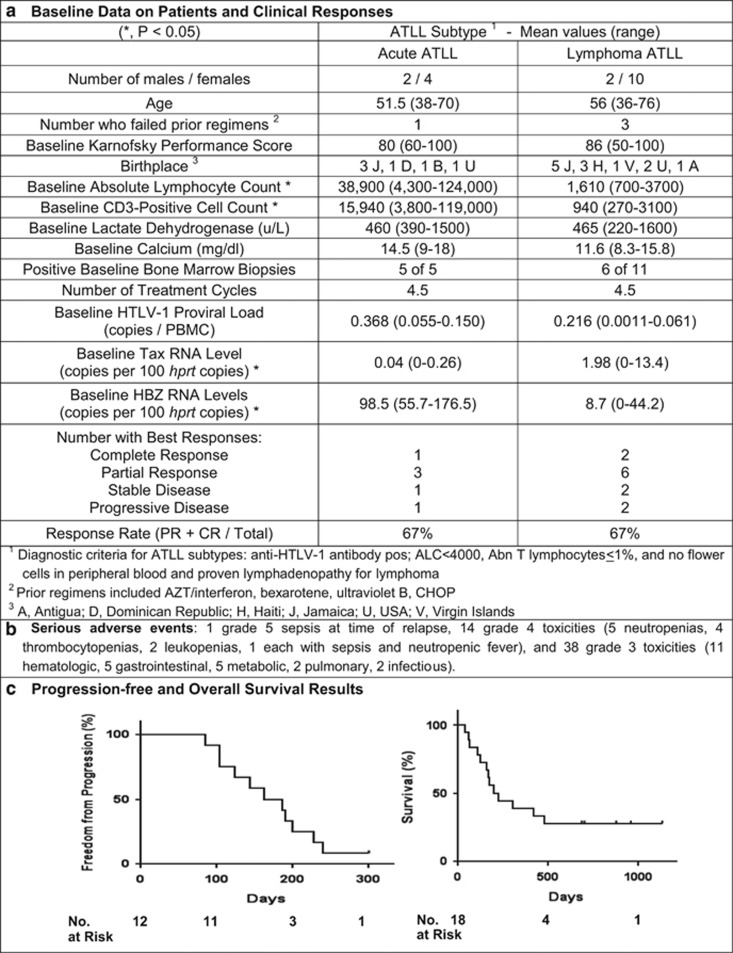
Baseline data, regimen toxicities and responses for patients. (**a**) Baseline clinical and virological data are provided for the 18 patients in the clinical trial, subdivided by acute versus lymphoma ATLL subtypes. (**b**) Serious adverse events during clinical trial participation are shown for these subjects. (**c**) Progression-free survival is shown for responders, as well as overall survival for all clinical trial participants. ALC, absolute lymphocyte count; AZT, azidothymidine; CHOP, cyclophosphamide, doxorubicin, vincristine, prednisone; CR, complete response; PR, partial response.

**Figure 2:**
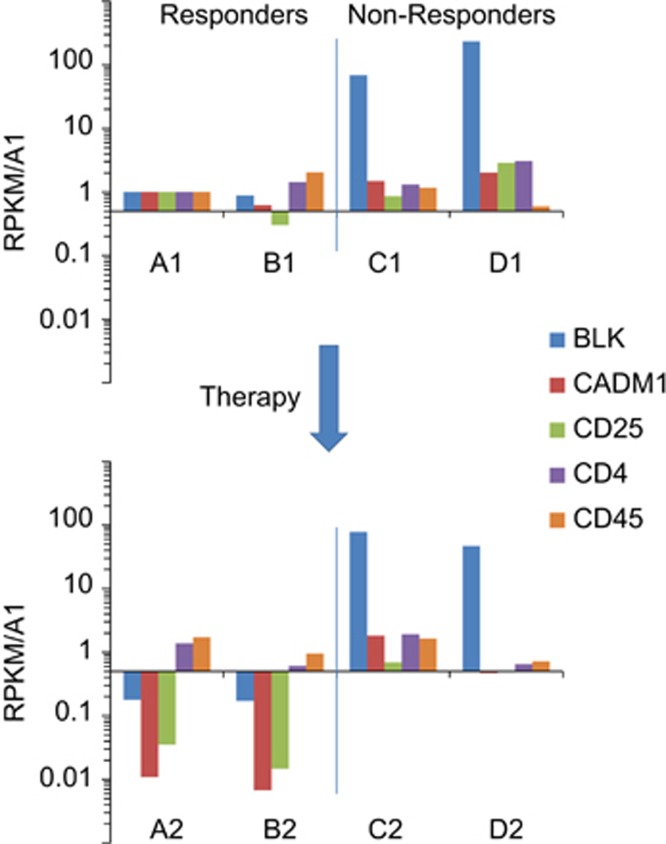
Blk expression is elevated in ATLL nonresponders. RNAseq was performed on RNA obtained from PBMCs collected from four patients (A, B, C and D) with acute disease before (upper graph) and after (lower graph) treatment with DA-EPOCH-based chemotherapy combined with bortezomib and raltegravir. Average reads per kilobase of transcript per million mapped reads (RPKM) values normalized to patient A before therapy (A1) are shown for protein-coding transcripts from the five genes indicated. CADM1 and CD25 are markers for ATLL and CD45 (PTPRC) is a panleukocyte marker.
