# Supplementary material for: Dose-adjusted EPOCH chemotherapy with bortezomib and raltegravir for human T-cell leukemia virus-associated adult T-cell leukemia lymphoma
Source: Blood Cancer J. 2016 Mar 25;6(3):e408–. doi: 10.1038/bcj.2016.21 (PMC4817103; doi:10.1038/bcj.2016.21)
Supplement: Supplementary Information [file bcj201621x1.pdf]

CONSORT

TRANSPARENT REPORTING of TRIALS

CONSORT 2010 Flow Diagram

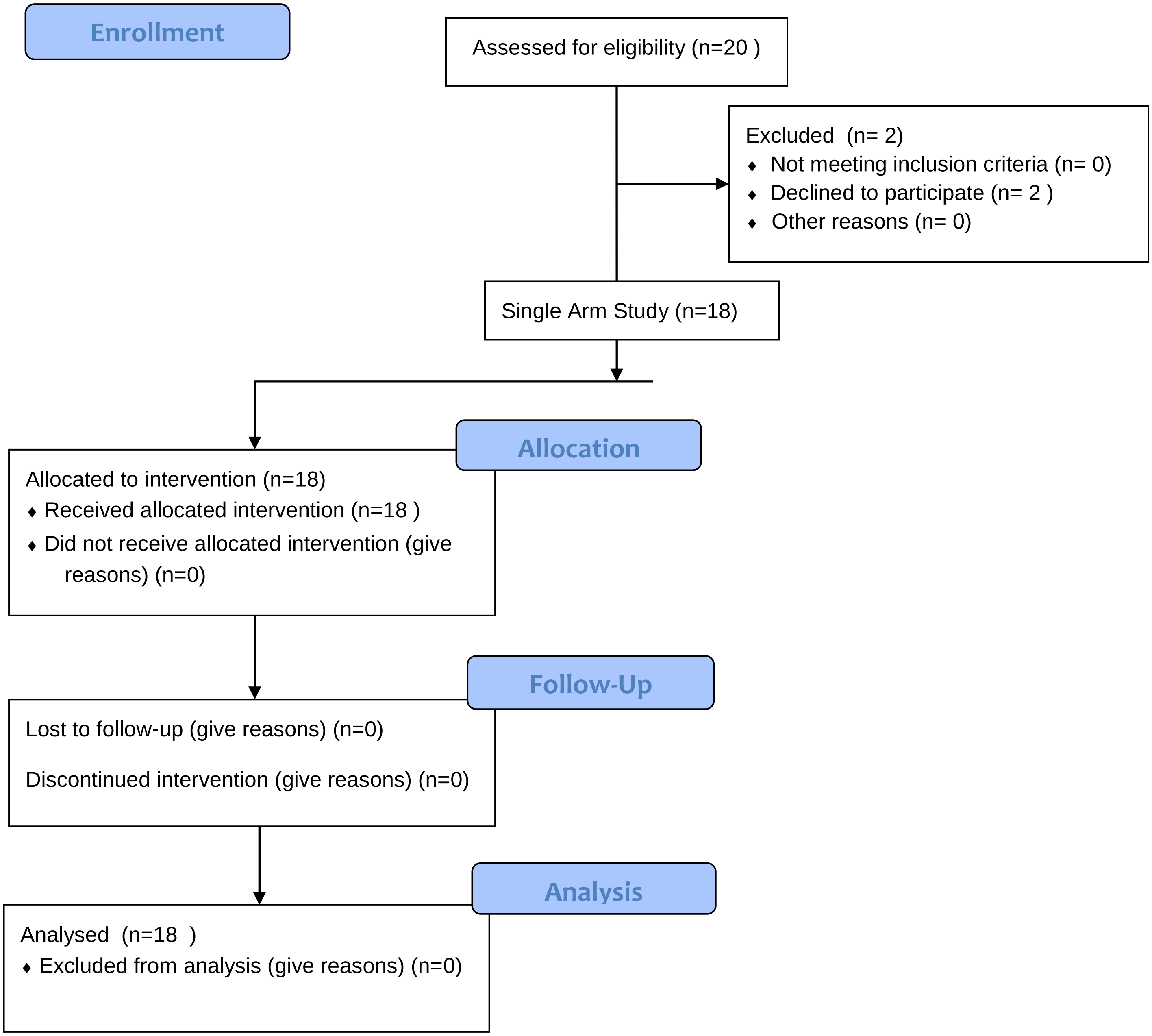

Phase I/II Trial of Dose-Adjusted EPOCH Chemotherapy with Bortezomib Combined with Integrase Inhibitor Therapy for HTLV-1 Associated T-Cell Leukemia Lymphoma

Washington University School of Medicine
Division of Oncology
660 South Euclid Avenue, Box 8056, St. Louis, MO 63110

Protocol #: 09-1758 / 201108212

Coordinating Center: **Washington University**

Principal Investigator/: Protocol Chair
Lee Ratner, M.D., Ph.D.
Washington University School of Medicine
Division of Oncology
660 S Euclid Ave, Box 8069, St Louis MO 63110
Telephone: 314-362-8836
Fax: 314-747-2120
Email: lratner@dom.wustl.edu

Protocol Co-Chair
Ariela Noy, M.D.
Lymphoma Service
Memorial Sloan Kettering Cancer Center
1275 York Avenue, New York, NY10065
Telephone: 212-639-7423
Fax: 646-422-2284
Email: noya@MSKCC.ORG

Statistician:
Philip Miller
Washington University School of Medicine
Division of Biostatistics
660 S Euclid Ave, Box 8067, St Louis MO 63110
Telephone: 314-362-3617
Fax: 314-362-3440
Email: phil@wubios.wustl.edu

**Sub Investigators:**	**Institution:**	**Modality:**
Juan Carlos Ramos, M.D	University of Miami	Medical Oncology
Samir Parekh,, M.D.	Montefiore Medical Center	Medical Oncology
Stefan Barta,M.D.	Montefiore Medical Center	Medical Oncology
Ariela Noy, M.D.	Memorial Sloan Kettering	Hematology/ Oncology
Richard Ambinder, M.D., Ph.D.	Johns Hopkins University	Medical Oncology
Adrienne A. Phillips, M.D., MPH	Columbia University	Hematology/Oncology

Study Drugs: **Bortezomib, Raltegravir**
IND#: 106994 EXEMPT

CONFIDENTIAL
The information contained in this document is regarded as confidential and, except to the extent necessary to obtain informed consent, may not be disclosed to another party unless law or regulations require such disclosure. Persons to whom the information is disclosed must be informed that the information is confidential and may not be further disclosed by them.

Protocol Revisions History

Initial Approval	12/15/09
Amendment	05/27/10
Amendment	09/02/10
Amendment	12/30/10
Amendment	12/23/11
Amendment	01/27/12 (QASMC only approved)
Amendment	04/06/12
Amendment	06/15/13

PRINCIPAL INVESTIGATOR SIGNATURE PAGE

Principal Investigator:

______________________________ __________

Signature of Investigator Date

____

Printed Name of Investigator

By my signature, I agree to personally supervise the conduct of this study and to ensure its conduct in compliance with the protocol, informed consent, IRB/HRPO procedures, the Declaration of Helsinki, ICH Good Clinical Practices guidelines, and the applicable parts of the United States Code of Federal Regulations or local regulations governing the conduct of clinical studies.

Investigator Contact Information

Study Principal Investigator

Lee Ratner M.D., Ph.D.
Washington University School of Medicine
Division of Oncology
660 S Euclid Ave, Box 8069
St Louis MO 63110
Telephone: 314-362-8836
Fax: 314-747-2120
Email: lratner@dom.wustl.edu

Sub-Investigators

Juan Carlos Ramos, M.D.
University of Miami Hospital/Sylvester
1475 NW 12th Ave.
Miami, FL 33136
Phone: 305-243-4909
Fax: 305-243-5885
Email: jramos2@med.miami.edu

Samir Parekh, M.D.
Montefiore Medical Center
111 East 210th Street
Bronx, NY 10467
Phone: 718-920-4057
Fax: 718-798-7474
Email: sparekh@montefiore.org

Stefan Barta, M.D.
Montefiore Medical Center
111 East 210th Street
Bronx NY 10467
Phone:718-920-4826
Fax:718-798-7474
Email:sbarta@montefiore.org

Ariela Noy M.D.
Memorial Sloan Kettering Cancer Center
1275 York Avenue, New York, NY10065
Telephone: 212-639-7423
Fax: 646-422-2284
Email: noya@MSKCC.ORG

Richard Ambinder, M.D.
The Johns Hopkins Hospital
401 N. Broadway
Baltimore, MD 21231
Phone: 410-955-8839
Fax: 410-955-0960
Email: ambinri@jhmi.edu
Adrienne A. Phillips MD, MPH
Columbia University, College of Physicians and Surgeons

Division of Hematology/Oncology
Herbert Irving Pavilion
161 Fort Washington Avenue, Room 9-907
New York, NY 10032
Phone: 212-305-3606
Fax: 212-305-6891
Email: ap2027@columbia.edu

Washington University Study Team Contact Information

Protocol Coordinator
Lee Ratner M.D., Ph.D.
Washington University School of Medicine
Division of Oncology
660 S Euclid Ave, Box 8069
St Louis MO 63110
Telephone: 314-362-8836
Fax: 314-747-2120
Email: lratner@dom.wustl.edu

SCHEMA

Population

20 patients with HTLV-1 associated ATLL

Treatment

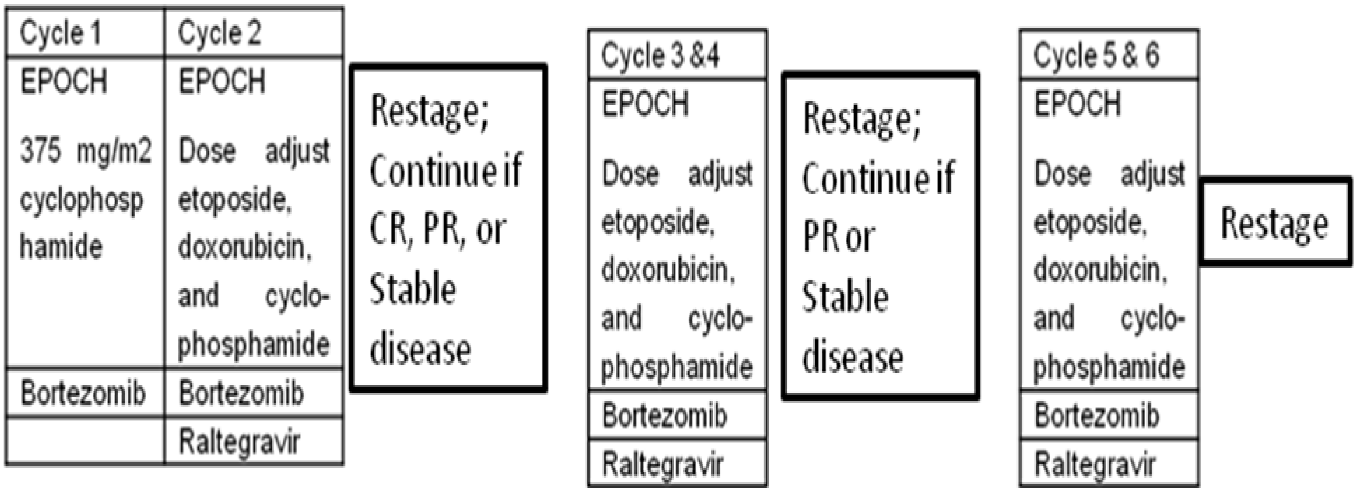

Bortezomib 1.0 mg/m^2 is infused on days 1 and 4

Etoposide 50 mg/m^2/d given as a continuous 96 hr IV infusion on days 1-4

Vincristine 0.4 mg/m^2/d given as a continuous 96 hrs IV infusion on day 1-4

Doxorubicin 10 mg/m^2/d given as a continuous 96 hrs IV infusion on days 1-4

Prednisone 60 mg/m^2/d given orally on days 1-5

Cyclophosphamide (day 5) 375 mg/m^2 given IV on day 5 over 30 min

Cycles 2-6:
If ANC nadir >500/mm3 and platelet nadir >25,000/mm3 in the previous cycle, then increase doses of etoposide, doxorubicin, and cyclophosphamide doses by 20%. If ANC nadir <500/mm3 on at least 3 measurements OR nadir platelet count less than 25,000/mm3 on at least 1 measurement during the previous cycle, then decrease etoposide, doxorubicin, and cyclophosphamide doses by 20%. Only cyclophosphamide doses are reduced at dose levels -1, -2 and -3.

Note: **Cycle 2**: Cyclophosphamide, etoposide, and doxorubicin doses can be escalated by 20% if the investigator feels grade 3-4 febrile neutropenia was precipitated by marrow infiltration by malignant cells

Cycle 3-6: The cyclophosphamide, etoposide, and doxorubicin doses should be escalated only if there is no previous episode in cycles 2-5 of grade 3-4 febrile neutropenia or any other toxicity that required a dose reduction of cyclophosphamide

Raltegravir is given orally 400 mg bid every day starting with cycle 2 therapy

Note: See section 7.0 for dose modifications for hematololgic and non-hematologic toxicity.

Cycles will be repeated every 21-28 days for two cycles beyond best response, or a maximum of 6 cycles. Best response is the response achieved when 1 or more additional cycles of chemotherapy are given and no additional tumor shrinkage is noted. That may include stable or progressive disease after 2 cycles of chemotherapy.

Plus: Neulasta 6 mg SQ on days 6, 7, or 8 or G-CSF 5 ug/kg (rounded to nearest 300 or 480 mcg) SQ daily for 10 days starting on day 6 or until the absolute neutrophil account has recovered to >4000 cells/mm3.

TABLE OF CONTENTS

SCHEMA 7
1.0 INTRODUCTION 12
1.1 HTLV-1 Infection 12
1.2 HTLV-1 Associated Adult T-Cell Leukemia Lymphoma 12
1.3 HTLV-1 Proviral DNA and Cell-Associated and Virion RNA Load and Protein Expression 13
1.4 HTLV-1 Clonality 13
1.5 Apoptosis Markers in HTLV-1 Infected Cells 13
1.6 Treatment of HTLV-1 Associated non-Hodgkin's Lymphoma 13
1.7 Antiviral Therapy for HTLV-1 Infection 14
1.8 EPOCH Chemotherapy 15
1.9 Bortezomib for Injection 15
1.9.1 Scientific Background 15
1.9.2 Nonclinical Pharmacology 17
1.9.3 Nonclinical Toxicity 17
1.9.4 Clinical Pharmacokinetics and Pharmacodynamics 18
1.9.5 Clinical Experience 19
1.9.6 Potential Risks of Bortezomib 21
1.10 Rationale 27
2.0 OBJECTIVES 28
2.1 Primary Endpoint 28
2.2 Secondary Endpoints 28
3.0 PATIENT SELECTION 28
3.1 Inclusion Criteria 28
3.2 Exclusion Criteria 29
4.0 REGISTRATION PROCEDURES 30
4.1 Enrollment/Registration Process 30
4.2 Confirmation of Patient Eligibility 30
4.3 Patient Registration in the Siteman Cancer Center Database 31
4.4 Assignment of UPN 31
5.0 CLINICAL AND LABORATORY EVALUATIONS 31
5.1 Baseline/Pretreatment Evaluation (See Appendix IV) 31
5.2 Evaluations During Chemotherapy Treatment (See Appendix IV). 32
5.3 Post-Treatment Evaluation (See Appendix IV) 33
5.4 Early Discontinuation of Therapy 33
6.0 STUDY MEDICATIONS 34
6.1 Treatment 34
6.2 Bortezomib 35
6.2.1 Preparation, Reconstitution, and Dispensation 36
6.2.2 Bortezomib reconstitution 36
6.2.3 Packaging and Labeling 36
6.2.4 Administration 36
6.2.5 Bortezomib Return 37
6.2.6 Storage, Handling, and Accountability 37
6.3 Chemotherapy Medications 37
6.3.1 Etoposide 37
6.3.2 Vincristine Sulfate 39
6.3.3 Doxorubicin 40
6.3.4 Cyclophosphamide 42

6.3.5 Prednisone 43
6.4 Antiviral Medication 44
6.4.1 Preparation, Reconstitution, and Dispensation 44
6.4.2 Packaging and Labeling 44
6.4.3 Administration 45
6.4.4 Storage, Handling, and Accountability 45
6.4.5 Side-effects 45
6.5 Concurrent Medication 45
6.5.1 Antiretroviral Therapy 45
6.5.2 Pneumocystis Carinii Prophylaxis 45
6.5.3 Prevention of Tumor Lysis Syndrome (Cycle 1 only) 45
6.5.4 Anti-Infectives 46
6.5.5 Herpes Zoster Prophylaxis 46
6.5.6 Prohibited Concurrent Medication 46
6.6 CNS Prophylaxis 46
6.7 Meningeal Lymphoma 46
6.8 Radiotherapy 46
6.9 Treatment Compliance 47
7.0 DOSE MODIFICATION AND DELAY/TOXICITY MANAGEMENT 47
7.1 Bortezomib Modifications 47
7.1.1 Dosage in Patients with Hepatic Impairment 49
7.2 Chemotherapy Modifications: Hematologic Toxicity 49
7.3 Chemotherapy Modifications: Non-Hematologic Toxicity 50
7.3.1 Dose adjustment for hyperbilirubinemia 50
7.3.2 Dose Adjustment for Cardiac Toxicity 50
7.3.3 Dose Adjustment for Neutrotoxicity 50
7.3.4 Dose Adjustment for Renal Impairment 51
7.4 Antiviral Toxicity Management 51
8.0 CRITERIA FOR TREATMENTAND/OR STUDY PARTICIPATION DISCONTINUATION 51
8.1 Patient Withdrawal 51
9.0 REGULATORY AND REPORTING REQUIREMENTS 52
9.1 Multi-center Sites Timeframe for Reporting Adverse Events 52
9.2 Adverse Events (AEs) 53
9.3 Unanticipated Problems 53
9.4 Noncompliance 53
9.5 Serious Noncompliance 53
9.6 Reporting to the Human Research Protection Office (HRPO) and the Quality Assurance and Safety Monitoring Committee (QASMC) at Washington University: 54
9.7 Protocol Exceptions 54
9.8 Reporting of AE Information Following Study Completion 54
9.9 Reporting of Adverse Events at Participating Institutions 55
9.9.1 To the Washington University Research Patient Coordinator 55
9.10 Procedures for Reporting Drug Exposure During Pregnancy and Birth Events ..56
9.11 Monitoring of Adverse Events and Period of Observation 56
9.12 Reporting Requirement for Secondary Sites 56
9.13 Reporting to Secondary Sites 57
9.14 Multicenter Regulatory Requirements 57
9.15 Regular Conference Call Participation 58
10.0 DATA SUBMISSION SCHEDULE 58

10.1 Data Submission Guidelines 58
11.0 EVALUATION OF RESPONSE 60
11.1 Response Assessment 60
11.2 Definition of Response 60
12.0 DATA AND SAFETY MONITORING 63
12.1 Data Safety Monitoring Reports 63
12.2 Data and Safety Monitoring Committee (DSMC) Requirements 63
12.3 DSMC Membership 63
12.4 DSMC Responsibilities 64
12.5 DSMC Meetings 64
12.6 DSMC Recommendations 65
13.0 AUDITING 65
13.1 Auditing Process 65
14.0 STATISTICAL CONSIDERATIONS 66
14.1 Study objectives and endpoints 66
14.2 Sample Size Estimation and Accrual 66
14.3 Statistical Analysis Plan 67
14.4 Safety Monitoring Plan 67
15.0 ETHICAL AND REGULATORY CONSIDERATIONS 67
15.1 Good Clinical Practice 67
15.2 Ethical Considerations 67
15.3 Informed Consent 68
15.4 Women and Minorities 69
15.5 Research Authorization 69
15.6 Subject Confidentiality 69
15.7 Protocol Compliance 69
15.8 On-site Audits 69
15.9 Drug Accountability 70
15.10 Premature Closure of the Study 70
15.11 Product Complaints 70
15.12 Record Retention 71
16.0 REFERENCES 72
Appendix I: Karnofsky Performance Status Scale 76
Appendix II: Ann Arbor Staging Criteria 77
Appendix III: Central Pathology Review 78
Appendix IV: Study Calendar 79
Appendix V: Body Surface Area and Creatinine Clearance Calculations 81
Appendix VI: New York Heart Association Classification of Cardiac Disease 82
Appendix VII : FACT/GOG-Neurotoxicity Questionnaire, Version 4.0 83
Appendix VIII: Blood for HTLV-1 DNA Proviral Load, RNA, HTLV-1 Integrase Gene Sequence, Integration Site Analysis, & NFKB Expression Profiling 84

1.0 INTRODUCTION

1.1 HTLV-1 Infection

Human T-cell leukemia virus type 1 (HTLV-1) is a gamma retrovirus (1). It is closely related to simian T-cell leukemia virus type 1 (STLV-1) and phylogenetic trees show that STLV-1 and HTLV-1 strains are intermingled (2). A somewhat more distantly related virus, with 60% nucleotide sequence homology to HTLV-1, is HTLV-2. These infections can be distinguished by differences in antibody reactivity to recombinant proteins or by polymerase chain reaction assays. HTLV-1 infections are prevalent in southern Japan, the Caribbean, and many parts of Central and South America, the Middle East, and Africa, where 10-15% of the population is infected (1,3). In the United States, 0.25% of volunteer blood donors are infected with HTLV-1 or HTLV-2. Infections by both viruses are not unusual among intravenous drug abusers. HTLV-2 is endemic in native North, Central, and South American populations. The HTLV-1 genome includes *gag*, *pro, pol,* and *env* genes, encoding the inner capsid of the virion, the viral protease, the viral reverse transcriptase and integrase enzymes, and the glycoprotein on the virion surface necessary for viral entry (4). Regulatory proteins include Tax, a potent transcriptional trans-activator, and Rex, a mediator of nuclear export of viral RNAs. Accessory genes encode proteins designated, p12, p13, p27, p30, and HBZ whose functions remain to be fully characterized. There is considerable evidence that the Tax protein is critical important for the leukemogenic activity of the virus (5,6). Although most viral genes are not expressed in ATLL samples, Tax expression is usually detectable. Tax together with Ras is capable of transforming rodent fibroblasts, and when expressed in a retrovirus of *Herpes samirii* vector is capable of immortalizing human lymphocytes. Constitutive Tax expression in transgenic mice results in several different types of malignancies, whereas expression in the lymphoid compartment of a transgenic mouse results in a lymphoproliferative malignancy. Tax functions at transcriptional and post-transcriptional steps. Tax upregulates several transcriptional pathways to enhance the expression of the viral promoter as well as several interleukins and their receptors (e.g. IL2, IL2Rα, IL15, IL15Rα), cytokines (e.g. IFNγ, GM-CSF), adhesion molecules (e.g. ICAM-1, VCAM-1, VLA-4), growth promoting factors (e.g. c-myc, c-sis, c-fos, egr-1, cyclin D2, and E2F-1), and apoptosis inhibiting factors (e.g. bcl xL, A20). Moreover, Tax can directly bind and enhance or inhibit the activity of several growth promoting (e.g. cyclins D2 and D3, E2F) or inhibiting factors (e.g. p15, p16, and p53 tumor suppressor proteins and cell cycle checkpoint protein MAD1), respectively. In addition Tax promotes the phosphorylation and inactivation of the p53 tumor suppressor protein.

1.2 HTLV-1 Associated Adult T-Cell Leukemia Lymphoma

Although HTLV-2 is not clearly associated with a clinical disorder, HTLV-1 is closely associated with two clinical disorders (1). About 5% of HTLV-1 infected individuals eventually develop a form of myelopathy known as HTLV-1 myelopathy (HAM) or tropical spastic paraparesis (TSP). It is characterized by lower limb spasticity, bowel and bladder disturbances, and slow but steady progression over several years. Additionally, about 5% of HTLV-1 infected individuals develop a T-cell non-Hodgkin's lymphoma designated adult T-cell leukemia lymphoma (ATLL). It occurs almost

exclusively in individuals who acquired HTLV-1 as a result of breast feeding, although four or five decades are generally required before development of disease. ATLL is characterized by frequent blood and bone marrow involvement, hypercalcemia, and lytic bone lesions. It is most commonly a CD4+ leukemia/lymphoma, although occasional examples of CD8+ lymphoma have been described. ATLL may fit into a variety of pathological subtypes as classified by the International Working Party Formulation. Clinical classifications of ATLL have included a “smoldering” disorder characterized by rash and minimal blood involvement, as well as a chronic form of ATLL, which have median survivals of two years or more (7). In contrast, a “lymphomatous” form of ATLL, and an “acute” form of ATLL have median survivals of 3-6 months.

1.3 HTLV-1 Proviral DNA and Cell-Associated and Virion RNA Load and Protein Expression

Recently assays of HTLV-1 proviral DNA load using PCR techniques have been developed and applied to clinical samples. These studies demonstrated levels of about 0.02 copies/PBMC in asymptomatic individuals and 0.08 copies/PBMC in HAM patients(8). RT-PCR assays of cell-associated viral and virion RNA have been developed and used to demonstrate that the majority of infected cells do not express viral products (9). However, quantitative data on transcript levels has not been obtained with clinical samples.

1.4 HTLV-1 Clonality

In ATLL, there is a clonal expansion of T cells with clonal T-cell receptor gene rearrangements and clonal sites of provirus integration into chromosomal DNA, as determined by Southern blot hybridization (10). More recently, a ligation mediated PCR technique has been utilized as a more sensitive way to detect different clonal populations in asymptomatic infected individuals, HAM patients, and ATLL patients (11). These studies have demonstrated that the expanded clone of malignant ATLL has arisen from an oligoclonal expansion of HTLV-1 infected cells. These studies suggest that HTLV-1 dissemination can occur by viral replication and clonal expansion of infected cells.

1.5 Apoptosis Markers in HTLV-1 Infected Cells

HTLV-1 infected cells are resistant to inducers of apoptosis. This may be a result of Tax inactivation of the p53 gene, or up-regulation of apoptosis inhibitor A20 or bcl-XL (5, 6). Arsenic trioxide and Interferon Alpha-2a have been used to induce apoptosis in HTLV-1 infected T-cell lines (12).

1.6 Treatment of HTLV-1 Associated non-Hodgkin’s Lymphoma

Various chemotherapy regimens have been utilized to treat patients with ATLL with complete response rates of only about 30%, which are not durable (13-16). In a large

cooperative study in Japan, 4-year survival of patients with acute or lymphomatous forms of ATLL was only 4%.

Two reports describe major response rates of 58% and 100%, respectively, in the lymphomatous form of ATLL, with the use of a combination of azidothymidine and Interferon Alpha-2a (17, 18). However, other investigators reported lower response rates with this approach (19). Other approaches have included the use of antibodies to IL2Rα with or without conjugates to radioisotopes, or the use of an IL2-diptheria toxin product (20).

1.7 Antiviral Therapy for HTLV-1 Infection

Although the AZT/Interferon Alpha-2a combination was used in ATLL patients, it is unclear whether this combination had antiviral, antitumor, and/or immunomodulatory activity towards HTLV-1. A recent report of the use of lamivudine in 5 HAM patients suggested antiviral activity, as demonstrated by a 1 log median decrease in virus load (21). This was associated with clinical improvement in one patient. Use of anti-retroviral agents has recently been shown to decrease HTLV-1 proviral DNA (22).

Our recent studies have examined various diketone integrase inhibitors for HTLV-1 infection. We found that Raltegravir inhibited HTLV-1 replication with IC50 of 35,Raltegravir has been well tolerated in combination with chemotherapy in patients with HIV malignancies, and Raltegravir is not associated with myelosuppression (see section 6.4). Raltegravir is approved for treatment of HIV-1 infection in combination with other antiretroviral agents in treatment-experienced adult patients who have evidence of viral replication and HIV-1 strains resistant to multiple antiretroviral agents.

In addition to direct anti-viral effects, viral suppression may be necessary in the treatment of cancer. We demonstrated this in our recent phase II clinical trial of infusional chemotherapy with etoposide, doxorubicin, and vincristine, daily prednisone, and bolus cyclophosphamide (EPOCH) given for two to six cycles until maximal clinical response, and followed by antiviral therapy with daily zidovudine, lamivudine, and alpha interferon-2a for up to one year. Seven patients were on study for less than one month due to progressive disease or chemotherapy toxicity. Eleven patients achieved an objective response with median duration of response of thirteen months, and two complete remissions. During chemotherapy induction, viral RNA expression increased (median 190-fold), and virus replication occurred, coincident with development of disease progression. Thus, while EPOCH chemotherapy followed by antiretroviral therapy is an active therapeutic regimen for adult T-cell leukemia-lymphoma, viral reactivation during induction chemotherapy may contribute to treatment failure. Alternative therapies are sorely needed in this disease that simultaneously prevent virus expression, and are cytocidal for malignant cells.

1.8 EPOCH Chemotherapy

The current trial will utilize DA EPOCH chemotherapy, which provides a 4-day continuous infusion of etoposide, vincristine, and doxorubicin, in addition to an intravenous bolus of cyclophosphamide and 5 days of oral prednisone. The selection of infusional chemotherapy is based on the poor response durations of bolus chemotherapy (see section 1.2), and since EPOCH chemotherapy has produced complete remissions in 57-74% of aggressive newly diagnosed or refractory non-Hodgkin's lymphomas in the non-HIV patient population (23, 24). Moreover EPOCH chemotherapy used in aggressive HIV-associated non-Hodgkin's lymphomas has produced a complete remission rate of 78%, and was well tolerated (25). Our experience with EPOCH chemotherapy in 19 patients with ATLL has demonstrated efficacy and tolerability (26). Moreover, EPOCH chemotherapy is feasible in areas of the world in which HTLV-1 infection is endemic. In our previous EPOCH trial, dose adjustments of EPOCH were not utilized, and may have accounted for a high rate of toxicity during cycle 1. In the current trial, lower starting doses of EPOCH will be used in cycle 1 to improve tolerability.

1.9 Bortezomib for Injection

Bortezomib is a proteasome inhibitor that blocks degradation of several proteins, including inhibitor of NFκB (IκB). NFκB is overexpressed in a wide range of hematopoietic malignancies, including ATLL and is thought to contribute to chemotherapy resistance. Bortezomib has been shown to inhibit proliferation of ATLL cells in culture and animal model systems (27, 28). Moreover, there are several anecdotal reports of bortezomib induced responses in ATLL (29). Bortezomib has been shown to have activity in a wide range of lymphomas. It is active in treatment of patients with relapsed or refractory cutaneous T-cell lymphomas. Moreover, bortezomib has been found to be synergistic with various chemotherapeutic agents. Moreover, bortezomib has been combined with several different chemotherapy regiments, including EPOCH, in an ongoing NCI trial of refractory lymphomas (NCT00057902).

1.9.1 Scientific Background

Bortezomib for Injection is a small-molecule proteasome inhibitor developed by Millennium Pharmaceuticals, Inc., (Millennium) as a novel agent to treat human malignancies. Bortezomib is currently approved by the United States Food and Drug Administration (US FDA) for the treatment of patients with multiple myeloma (MM). It is also indicated for the treatment of patients with mantle cell lymphoma (MCL) who have received at least 1 prior therapy. In the European Union (EU), bortezomib in combination with melphalan and prednisone is indicated for the treatment of patients with previously untreated MM who are not eligible for high-dose chemotherapy with bone marrow transplant. Bortezomib is indicated as monotherapy for the treatment of progressive MM in patients who have received at least 1 prior therapy and who have already undergone or are unsuitable for bone marrow transplantation.

By inhibiting a single molecular target, the proteasome, bortezomib affects multiple signaling pathways. The anti-neoplastic effect of bortezomib likely involves several distinct mechanisms, including inhibition of cell growth and survival pathways, induction of apoptosis, and inhibition of expression of genes that control cellular adhesion, migration and angiogenesis. Thus, the mechanisms by which bortezom ibelicits its antitumor activity may vary among tumor types, and the extent to which each affected pathway is critical to the inhibition of tumor growth could also differ. Bortezomib has a novel pattern of cytotoxicity in National Cancer Institute (NCI) in vitro and in vivo assays (30). In addition, bortezomibhas cytotoxic activity in a variety of xenograft tumor models, both as a single agent and in combination with chemotherapy and radiation (31, 32, 33, 34, 35) Notably, bortezomib induces apoptosis in cells that over express bcl-2, a genetic trait that confers unregulated growth and resistance to conventional chemotherapeutics (36).

Bortezomib is thought to be efficacious in multiple myeloma via its inhibition of nuclear factor κB (NF-κB) activation, its attenuation of interleukin-6 (IL-6)-mediated cell growth, a direct apoptotic effect, and possibly anti-angiogenic and other effects (37).

Constitutive activation of nuclear factor kappaB (NF-kappaB) plays a major role in the pathogenesis of human T-cell lymphotrophic virus I-associated malignancy. Bortezomib decreased NF-kappaB DNA binding activity by preventing degradation of IkappaB(alpha). (Tan Waldmann 200Cancer Res. 2002 Feb 15;62(4):1083-6.) Proteasome inhibitors thus provide a rational approach to control constitutively activated NF-kappaB in human T-cell lymphotrophic virus I-infected T cells. Efficacy against ATL cells in vitro at least partly depends on the upregulation of Noxa and functional repression of Mcl-1, as is also the case in MM and malignant melanoma. . (Ri Cancer Sci. 2009 Feb;100(2):341-8.) In further experiments, Nasr et al. (Oncogene. 2005 Jan 13;24(3):419-30.**)** demonstrated bortezomib inhibition of cell proliferation and induction of apoptosis in fresh ATL cells, HTLV-I transformed and HTLV-I-negative malignant T cells, while normal resting or activated T lymphocytes were resistant. Combination of bortezomib and doxorubicin or etoposide resulted in an additive growth inhibition. In HTLV-I-negative malignant cells, bortzomib treatment significantly downregulated the antiapoptotic protein X-IAP and to a lesser extent c-IAP-1 and bcl-X(L) and resulted in caspase-dependent apoptosis. In HTLV-I transformed cells, the inhibition of the proteasomal degradation of Tax by PS-341 likely explained the relative protection of HTLV-I infected cells against caspase-dependent apoptosis. PS-341 treatment of these cells stabilized IkappaBalpha, IkappaBbeta, IkappaBvarepsilon, p21, p27 and p53 proteins and selectively inhibited Rel-A DNA binding NF-kappaB complexes. In both HTLV-I-positive and -negative cells, PS-341 treatment induced ceramide accumulation that correlated with apoptosis. They concluded that bortezomib affects multiple pathways critical for the survival of HTLV-I-positive and -negative malignant T cells supporting a potential therapeutic role for PS-341 in both ATL and HTLV-I-negative T-cell lymphomas, whether alone or in combination with chemotherapy.

In a murine model of adult T-cell leukemia, bortezomib alone did not yield prolongation of the survival of tumor-bearing mice. However, when combined with the current clinically approved drug humanized anti-Tac, therapy, bortezomib was associated with a complete remission in a proportion of treated animals, whereas only a partial response was observed in animals treated with humanized anti-Tac alone. (Same ref. Tan Waldmann Cancer Res. 2002 Feb 15;62(4):1083-6

1.9.2 Nonclinical Pharmacology

Pharmacokinetic (PK) and pharmacodynamic studies were conducted in the rat and cynomolgus monkey. Upon intravenous (IV) bolus administration, bortezomib displays a rapid distribution phase ($t½\alpha$ <10 minutes) followed by a longer elimination phase ($t½\beta$ 5–15 hours). Bortezomib has a large volume of distribution (range 5–50 L/kg). The plasma PK profile is well described by a 2-compartment model.

The pharmacodynamic action of bortezomib is well established and can be measured through an ex vivo assay (20S proteasome activity) (38). This assay was used to determine the duration of drug effect in lieu of the PK data in the early preclinical toxicology studies as well as to set a guide for dose escalation in humans. Following dosing with bortezomib in the rat and cynomolgus monkey, proteasome inhibition in peripheral blood had a half-life less than 24 hours, with proteasome activity returning to pretreatment baseline within 24 hours in monkey and within 48 to 72 hours in rat after a single dose of bortezomib. Further, intermittent but high inhibition (>70%) of proteasome activity was better tolerated than sustained inhibition. Thus, a twice-weekly clinical dosing regimen was chosen in order to allow return of proteasome activity towards baseline between dose administrations.

1.9.3 Nonclinical Toxicity

Single-dose IV toxicity studies were conducted with bortezomib in the mouse, rat, dog, and monkey to establish the single-dose maximum tolerated dose (MTD). The MTDs were 0.25 mg/kg (1.5 mg/m^2) and 0.067 mg/kg (0.8 mg/m^2) in the 2 most sensitive species, rat and monkey, respectively. Repeat-dose multi-cycle toxicity studies of 3 and 6 months in the rat and 9 months in the monkey, each with 8-week recovery periods, were conducted to characterize the chronic toxicity of bortezomib when administered by the clinical route and regimen of administration. The MTD in the 6-month rat study was 0.10 mg/kg (0.6 mg/m^2) and the key target organs were the gastrointestinal (GI) tract, hematopoietic and lymphoid systems. The MTD in the 9-month monkey study was 0.05 mg/kg (0.6 mg/m^2) and the key target organs were the GI tract, hematopoietic and lymphoid systems, peripheral nervous system, and kidney. Full or partial reversibility was observed for each of the toxicities described to date.

In general, the nature of the toxicity of bortezomibis similar across species, and target organs of toxicity in animals have been largely predictive of human toxicity. The toxicity of bortezomib in animals is characterized by a steep dose-response with mortality seen at dosages above the MTD. The cause of death at acutely lethal dosages is considered

to be related to indirect cardiovascular (CV) effects of hypotension and vascular changes with secondary bradycardia and the cause of death in long-term studies has been attributed to GI or hematologic toxicity. The pharmacologic effects of bortezomib on the CV system have been extensively characterized and have demonstrated that indirect effects on CV function occur only at acutely lethal dosages and are abrogated by routine supportive care. Additional detailed information regarding the nonclinical pharmacology and toxicology of bortezomib may be found in the Investigator's Brochure.

1.9.4 Clinical Pharmacokinetics and Pharmacodynamics

The clinical pharmacology characterization of bortezomib has been determined from phase 1 studies in subjects with solid tumors and hematological malignancies, and confirmed in phase 2 studies in subjects with multiple myeloma.

Bortezomib demonstrates multi-compartmental pharmacokinetics. Following intravenous administration of 1.0 mg/m^2 and 1.3 mg/m^2 dose, the mean first-dose maximum observed plasma concentrations of bortezomib were 57 and 112 ng/mL, respectively in 11 patients with multiple myeloma and creatinine clearance values >50 mL/min participating in a pharmacokinetics study. In subsequent doses, mean maximum observed plasma concentrations ranged from 67 to 106 ng/mL for the 1.0 mg/m^2 dose and 89 to 120 ng/mL for the 1.3 mg/m^2 dose. The mean elimination half-life of bortezomib upon multiple dosing ranged from 40 to 193 hours. Bortezomib is eliminated more rapidly following the first dose. Mean Total Body Clearances were 102 and 112 L/h following the first dose for doses of 1.0 mg/m^2 and 1.3 mg/m^2, respectively, and ranged from 15 to 32 L/h following subsequent doses for doses of 1.0 and 1.3 mg/m^2, respectively. Clinical experience has shown that the change in clearance does not result in overt toxicity from accumulation in this multidose regimen in humans.

In subjects with advanced malignancies, the maximum pharmacodynamic effect (inhibition of 20S activity) occurred within 1-hour post dose. At the therapeutic dose of 1.3 mg/m^2 in subjects with multiple myeloma, the mean proteasome inhibition at 1-hour post dose was approximately 61%.

The time course of proteasome inhibition in subjects is characterized by maximum inhibition observed within the first hour after administration, followed by partial recovery of proteasome activity over the next 6 to 24 hours to within 50% of the pretreatment activity. On the Day 1, 4, 8, and 11 schedule, variable (10%–30%) levels of proteasome inhibition have been observed at next scheduled dosing. In theory, this advantage allows cells to recover proteasome activity for normal cellular housekeeping functions between doses.

The relationship between bortezomib plasma concentrations and proteasome inhibition can be described by a maximum effect (E_{max}) model. The E_{max} curve is initially very steep, with small changes in plasma bortezomib concentration over the range of 0.5 to 2.0 ng/mL relating to large increases in the percent inhibition (0–60%). After that, a

plateau occurs where marginal increases of proteasome inhibition are observed in spite of large changes in plasma bortezomib concentrations.

1.9.5 Clinical Experience

It is estimated that more than 436,000 patients have been treated with bortezomib, including patients treated through Millennium-sponsored clinical trials, Investigator-Initiated Studies, the US NCI Cancer Therapy Evaluation Program (CTEP), and with commercially available drug. Bortezomib has been commercially available since 13 May 2003.

The overall goal of the Millennium phase 1 program was to determine the MTD and dose-limiting toxicity (DLT) of bortezomib in a number of therapeutic settings involving subjects with various advanced malignancies. In a Phase I trial in patients with refractory hematologic malignancies, the MTD for a twice weekly for 4 weeks of a 42 day cycle was 1.04 mg/m^2/dose, with DLTs of thrombocytopenia, hyponatremia, hypokalemia, fatigue, and malaise (39). The toxicity was greatest during the third and fourth weeks of therapy. In the 3-week schedule of bortezomib monotherapy (4 doses, given on Days 1, 4, 8, and 11 of a 21-day treatment cycle), the DLT occurred at 1.56 mg/m^2/dose (3 subjects with Grade 3 diarrhea and 1 with peripheral sensory neuropathy). Therefore, the MTD at this schedule was 1.3 mg/m^2/dose. In a 35-day treatment cycle with 4 weekly doses of bortezomib monotherapy, the MTD was 1.6 mg/m^2/dose and DLT included hypotension, tachycardia, diarrhea, and syncope.

In phase 1 clinical studies, anti-tumor activity was reported in subjects with NHL, multiple myeloma, Waldenström's Macroglobulinemia, squamous cell carcinoma of the nasopharynx, bronchoalveolar carcinoma of the lung, renal cell carcinoma, and prostate cancer.

The safety and efficacy of bortezomib in subjects with multiple myeloma were investigated in two phase 2 clinical studies, studies M34100-024 (subjects with first relapse) (40) and M34100-025 (subjects with second or greater relapse and refractory to their last prior therapy) (41). In M34100-025, 202 heavily pre-treated subjects with refractory multiple myeloma after at least 2 previous treatments received bortezomib, 1.3 mg/m^2 on Days 1, 4, 8, and 11 of a 21-day treatment cycle. The European Group for Blood and Marrow Transplant (EBMT) response criteria, as described by Blade (42) were utilized to determine disease response. CRs were observed in 4% of subjects, with an additional 6% of patients meeting all criteria for CR but having a positive immunofixation test. PR or better was observed in 27% of subjects, and the overall response rate (CR, PR and minor response [MR] combined) was 35%. Seventy percent of subjects experienced stable disease or better.

The phase 3 study (M34101-039) (43), also referred to as the APEX study, was designed to determine whether bortezomib provided benefit (time to progression [TTP], response rate, and survival) to patients with relapsed or refractory MM relative to treatment with high-dose dexamethasone. The study was also designed to determine

the safety and tolerability of bortezomib relative to high-dose dexamethasone, and whether treatment with bortezomib was associated with superior clinical benefit and quality of life relative to high-dose dexamethasone. A total of 669 patients were enrolled and 663 patients received study drug (bortezomib: 331; dexamethasone: 332). Patients randomized to bortezomib received 1.3 mg/m^2 I.V. push twice weekly on days 1, 4, 8, and 11 of a 3-week cycle for up to eight treatment cycles as induction therapy, followed by 1.3 mg/m^2 bortezomib weekly on days 1, 8, 15, and 22 of a 5-week cycle for three cycles as maintenance therapy. Patients randomized to dexamethasone received oral dexamethasone 40 mg once daily on days 1 to 4, 9 to 12, and 17 to 20 of a 5-week cycle for up to four treatment cycles as induction therapy, followed by dexamethasone 40 mg once daily on days 1 to 4 followed of a 4-week cycle for five cycles as maintenance therapy. The European Group for Blood and Marrow Transplant (EBMT) response criteria, as described by Blade (42) were utilized to determine disease response. There was a 78% increase in TTP for the bortezomib arm. Median TTP was 6.2 months for the bortezomib arm and 3.5 months for the dexamethasone arm (*P*<.0001). CR (complete response) + PR (partial response) was 38% with bortezomib vs. 18% with dexamethasone (*P*<.0001). CR was 6% with bortezomib vs. <1% with dexamethasone (*P*<.0001). The CR + nCR rate was13% with bortezomib vs. 2% with dexamethasone. In patients who had received only one prior line of treatment (bortezomib: 132; dexamethasone: 119), CR + PR was 45% with bortezomib vs. 26% with dexamethasone (*P*=.0035). With a median 8.3 months of follow-up, overall survival was significantly longer (*P*=.0013) for patients on the bortezomib arm vs. patients on the dexamethasone arm. The probability of survival at one year was 80% for the bortezomib arm vs. 66% for the dexamethasone arm, which represented a 41% decreased relative risk of death in the first year with bortezomib (*P*=.0005). In patients who had received only one prior line of treatment, the probability of survival at one year was 89% for the bortezomib arm vs. 72% for the dexamethasone arm, which represented a 61% decreased relative risk of death in the first year with bortezomib (*P*=.0098). Updated response rates and survival data were reported for M34101-039 (44). The updated CR (complete response) + PR (partial response) rate was 43% with bortezomib. The CR + nCR rate was 16% with bortezomib. With a median 22 months of follow-up, overall survival was significantly longer for patients on the bortezomib arm vs. patients on the dexamethasone arm. The median overall survival was 29.8 months (95% CI: 23.2, not estimable) for the bortezomib arm vs 23.7 months (95% CI: 18.7, 29.1) for the dexamethasone arm (hazard ratio = 0.77, *P*= 0.0272). The probability of survival at one year was 80% for the bortezomib arm vs. vs 67% for the dexamethasone arm (*P*=0.0002).

Studies using bortezomib as monotherapy and in combination with other chemotherapy agents are continuing. Most recently, Dunleavy et al. (Blood **June 11, 2009** vol. 113 no. 24 **6069-6076**) showed dose adjusted dose EPOCH could be combined with bortezomib at an MTD of 1.5 mg/m2 in patients with relapsed and refractory diffuse large B cell lymphoma (DLBCL). Bortezomib alone had no activity in DLBCL, but when combined with chemotherapy, it demonstrated a significantly higher response (83% vs 13%; *P* < .001) and median overall survival (10.8 vs 3.4 months; *P* = .003) in the

activated B cell subtype compared with the germinal center subtype DLBCL, respectively.

In contrast, single agent activity of bortezomib has been anecdotally reported to be active in ATLL, (reference 29). Using gene expression arrays, Pise-Maisison et al. identified BIRC5 among other genes overexpressed in ATLL. Blood. 2009 Apr 23;113(17):4016-26. Epub 2009 Jan 8. shRNA decreased BIRC5 expression and cell viability 80%. Clinical treatment of an ATL patient with bortezomib decreased BIRC5 expression and cell viability as well. To gain further insight into the cellular factors involved in sensitivity to bortezomib, they examined expression of several antiapototic factors. Indeed, they found that, as with the mRNA level, the level of BIRC5 protein decreased with bortezomib treatment. In contrast, the antiapototic factors XIAP and Bcl-xL did not decrease. As expected, treatment with bortezomib caused an increase in IκBα levels, indicating blockage of the proteasome.

1.9.6 Potential Risks of Bortezomib

To date, more than 436,000 patients have been treated with bortezomib in both clinical trials investigating its use in hematological malignancies and solid tumors, and in patients who were treated with commercially available bortezomib.

Prescribing physicians and health care practitioners are referred to their locally approved product label for bortezomib regarding Indications and Usage, Contraindications, Warnings, and Precautions.

The known anticipated risks of bortezomib therapy are presented in Table 1.1 and Table 1.2 These risks are grouped according to the combined frequency observed in an integrated analysis of AEs in sponsored clinical studies of single-agent bortezomib dosed at 1.3 mg/m^2 twice weekly on a 21-day schedule, in patients with multiple myeloma and mantle cell lymphoma.

Table 1-1 Known Anticipated Risks of Bortezomib by MedDRA System Organ Class, Observed Incidence, and Preferred Term

System Organ Class<br>Observed Incidence	Preferred Term
Blood and Lymphatic System Disorders	
Most common	Thrombocytopenia*, anaemia*
Very common	Neutropenia*
Common	Lymphopenia, pancytopenia*, leukopenia*, febrile neutropenia
Cardiac Disorders	
Common	Tachycardia, atrial fibrillation, palpitations, cardiac failure congestive*
Uncommon	Cardiogenic shock*, atrial flutter, cardiac tamponade*±, bradycardia, atrioventricular block complete, arrhythmia, cardiac arrest*, cardiac failure, arrhythmia, pericardial effusion, pericarditis, pericardial disease±, cardiopulmonary failure±
Ear and Labyrinth Disorders	
Uncommon	Deafness, hearing impaired
Eye Disorders	
Common	Blurred vision, conjunctivitis, conjunctival haemorrhage
Gastrointestinal Disorders	
Most common	Constipation, diarrhoea*, nausea, vomiting*
Very common	abdominal pain (excluding oral and throat)
Common	Dyspepsia, pharyngolaryngeal pain, gastroesophageal reflux, abdominal distension, gastritis, stomatitis, mouth ulceration, dysphagia, gastrointestinal haemorrhage*, lower gastrointestinal haemorrhage*± rectal haemorrhage
Uncommon	Eructation, gastrointestinal pain, tongue ulceration, retching, upper gastrointestinal haemorrhage*, haematemesis*, oral mucosal petechiae, ileus paralytic*, ileus, odynophagia, enteritis, colitis, oesophagitis, enterocolitis, diarrhoea haemorrhagic, acute pancreatitis*, intestinal obstruction
General Disorders and Administration Site Conditions	
Most common	Fatigue, pyrexia
Very common	Chills, oedema peripheral, asthenia

Table 1-1 Known Anticipated Risks of Bortezomib by MedDRA System Organ Class, Observed Incidence, and Preferred Term

System Organ Class<br>Observed Incidence	Preferred Term
Common	Neuralgia, lethargy, malaise, chest pain, mucosal inflammation*
Uncommon	Injection site pain, injection site irritation, injection site phlebitis, general physical health deterioration*, catheter-related complication
Hepatobiliary Disorders	
Uncommon	Hyperbilirubinaemia, hepatitis*±
Immune System Disorders	
Uncommon	Drug hypersensitivity, angioedema
Infections and Infestations	
Very common	Upper respiratory tract infection, nasopharyngitis, pneumonia*, Herpes zoster*
Common	Lower respiratory tract infection*, sinusitis, pharyngitis, oral candidiasis, urinary tract infection*, sepsis*, bactaeremia*, cellulitis*, Herpes simplex, bronchitis, gastroenteritis*, infection
Uncommon	Septic shock*, catheter-related infection*, skin infection*, Herpes zoster disseminated*, lung infection*, infusion site cellulitis, catheter site cellulitis, infusion site infection, urosepsis*, Aspergillosis*, tinea infection, Herpes zoster ophthalmic, Herpes simplex ophthalmic, meningoencephalitis herpetic±, varicella, empyema±, fungal oesophagitis±
Injury, Poisoning, and Procedural Complications	
Common	Fall
Uncommon	Subdural haematoma
Investigations	
Common	Weight decreased, alanine aminotransferase (ALT) increased, aspartate aminotransferase (AST) increased, blood alkaline phosphatase increased, liver function test abnormal, blood creatinine increased*
Uncommon	Gamma-glutamyltransferase (GGT) increased, oxygen saturation decreased*, blood albumin decreased, ejection fraction decreased*
Metabolism and Nutritional Disorders	
Very common	Decreased appetite, anorexia, dehydration*

Table 1-1 Known Anticipated Risks of Bortezomib by MedDRA System Organ Class, Observed Incidence, and Preferred Term

System Organ Class<br>Observed Incidence	Preferred Term
Common	Hyperglycaemia, hypoglycaemia, hyponatraemia, hypokalaemia, hypercalcaemia*
Musculoskeletal and Connective Tissue Disorders	
Very common	Bone pain, myalgia, arthralgia, back pain
Common	Muscular weakness
Uncommon	Limb discomfort
Neoplasms, Benign, Malignant, and Unspecified (including cysts and polyps)	
Uncommon	Tumour lysis syndrome*
Nervous System Disorders	
Most common	Peripheral neuropathy (including all preferred terms under the MedDRA High-level term Peripheral neuropathy NEC)
Very common	Paresthesia, dizziness excluding vertigo, headache
Common	Polyneuropathy, syncope, dysesthesia, dysgeusia, postherpetic neuralgia
Uncommon	Convulsion, loss of consciousness, ageusia, encephalopathy, paralysis*,autonomic neuropathy, reversible posterior leukoencephalopathy syndrome±
Psychiatric Disorders	
Very common	Anxiety, insomnia
Common	Confusional state
Uncommon	Delirium
Renal and Urinary Disorders	
Common	Renal impairment*, renal failure*, haematuria
Uncommon	Micturition disorder
Respiratory, Thoracic, and Mediastinal Disorders	
Very common	Cough, dyspnoea
Common	Epistaxis, dyspnoea exertional, pleural effusion*, rhinorrhea, hypoxia*, pulmonary oedema*
Uncommon	Hemoptysis*, acute respiratory distress syndrome*, respiratory failure*, pneumonitis*, lung infiltration, pulmonary alveolar haemorrhage*, interstitial lung disease*, pulmonary hypertension*, pleurisy, pleuritic

Table 1-1 Known Anticipated Risks of Bortezomib by MedDRA System Organ Class, Observed Incidence, and Preferred Term

System Organ Class<br>Observed Incidence	Preferred Term
	pain
Skin and Subcutaneous Tissue Disorders	
Very common	Rash
Common	Rash pruritic, rash erythematous, urticaria, petechiae
Uncommon	Cutaneous vasculitis, leukocytoclastic vasculitis±
Vascular Disorders	
Common	Hypotension*, orthostatic hypotension
Uncommon	Cerebral haemorrhage*

Source: VELCADE® (bortezomib) for Injection Investigator's Brochure Edition 14.

Most common = ≥ 30%, Very common = 10% to 29%, Common = 1% to 9%, Uncommon = < 1%.

* Fatal outcomes have been reported.

± Indicates a Preferred term not listed in the source table, however the event is deemed medically important and so is included.

Table 1-2 Reports of Adverse Reactions From Postmarketing Experience

System Organ Class<br>Preferred Term	Observed Incidence[a]
Blood and lymphatic system disorders	
Disseminated intravascular coagulation	Rare
Cardiac Disorders	
Atrioventricular block complete	Rare
Cardiac tamponade	Rare
Ear and labyrinth disorders	
Deafness bilateral	Rare
Eye Disorders	
Ophthalmic herpes	Rare
Optic neuropathy	Rare
Blindness	Rare
Gastrointestinal Disorders	
Acute pancreatitis	Rare
Ischemic colitis	Rare
Hepatobiliary disorders	
Hepatitis	Uncommon
Liver failure	Unknown
Infections and infestations	
Herpes meningoencephalitis	Rare
Septic shock	Rare
Immune System Disorders	
Angioedema	Rare
Nervous System Disorders	
Autonomic neuropathy	Rare
Dysautonomia	Unknown
Encephalopathy	Rare
Respiratory, thoracic and mediastinal disorders:	
Acute diffuse infiltrative pulmonary disease[b]	Rare
Acute respiratory distress syndrome	Rare

Table 1-2 Reports of Adverse Reactions From Postmarketing Experience

(ARDS)	
Interstitial pneumonia	Rare
Lung infiltration	Rare
Pneumonitis	Rare
Pulmonary hypertension	Rare
Skin and subcutaneous system disorders	
Acute febrile neutrophilic dermatosis	Unknown
Toxic epidermal necrolysis	Unknown

Source: VELCADE® (bortezomib) for Injection Investigator's Brochure Edition 14.

a Incidence is assigned using the following convention: very common (≥ 1/10); common (≥ 1/100 and < 1/10); uncommon (≥ 1/1000 and < 1/100); rare (≥ 1/10,000 and < 1/1000); very rare (< 1/10,000, including isolated reports).

b Acute diffuse infiltrative pulmonary disease is a MedDRA Lower Level Term which corresponds to a Preferred Term of Interstitial lung disease.

Other medical events of interest that are considered not causally related to bortezomib include hepatic failure and QT prolongation. Fatal outcomes have been reported.

Women of childbearing potential should avoid becoming pregnant while being treated with bortezomib. Genotoxicity testing has shown that bortezomib is negative in the in vitro Ames assay and in the in vivo micronucleus assay, but it is a clastogen in the in vitro chromosomal aberration assay.

Additional details on the potential risks of bortezomib may be found in the Investigator's Brochure.

1.10 Rationale

The rationale of the current study is to explore the use of combination chemotherapy together with antiretroviral agents in order to determine the efficacy and toxicity of this approach, while also examining markers of virus replication and expression, and tumor cell proliferation to gain understanding of the biological basis of this malignancy and to identify predictors of response.

2.0 OBJECTIVES

2.1 Primary Endpoint

To determine the tolerability and efficacy (response rate) of dose adjusted bortezomib-EPOCH (DA bortezomib-EPOCH) chemotherapy combined with Raltegravir in patients with HTLV-1 associated leukemia/lymphoma (ATLL).

2.2 Secondary Endpoints

1. To determine the duration of response of DA bortezomib-EPOCH chemotherapy combined with Raltegravir for HTLV-1 associated leukemia/lymphoma (ATLL).
2. To evaluate the effects of DA bortezomib-EPOCH chemotherapy combined with Raltegravir on HTLV-1 DNA and RNA load, HTLV-1 integrase gene sequence, and HTLV-1 integration sites. To determine if relapsed or progressive disease is a result of renewed virus replication.
3. To evaluate the relation of NFκB gene expression profile on response to DA bortezomib-EPOCH chemotherapy combined with Raltegravir.

3.0 PATIENT SELECTION

The total number of patients to be enrolled on this study is 20. Enrollment is defined as the first day of bortezomib treatment (i.e., Day 1 of Cycle 1).

3.1 Inclusion Criteria

1. Histologically or cytologically documented ATLL. Patients with previously untreated or treated ATLL are eligible.
2. Tumors must be CD3 positive (>50% cells express CD3).
3. Documented HTLV-1 infection: documentation may be serologic assay (ELISA, Western blot). Confirmation ofHTLV-1 rather than HTLV-2 by differential Western blot (e.g. Genelabs Diagnostics HTLV Blot 2.4) or PCR is desirable but his result is not required prior to trial enrollment.
4. Measurable disease must be present. These nodes or masses should be selected according to all of the following: they should be clearly measurable in at least two perpendicular dimensions; if possible they should be from disparate regions of the body; and they should include mediastinal and retroperitoneal areas of disease whenever these sites are involved. For patients with acute (leukemic) form of ATLL, measureable disease can be derived from CD4+ lymphocyte flow data on the peripheral blood and/or bone marrow.
5. All stages are eligible.
6. Adequate hematologic function within 14 days before enrollment: ANC >1000 cells/mm3, platelet count >75,000 cells/mm3 unless cytopenias are secondary to ATLL. All patients must be off hematologic growth factors for at least 24 hrs.

7. Adequate hepatic function, transaminase <3 times the upper limit of normal unless due to Gilbert's disease or hepatic involvement by tumor; total bilirubin ≤1.5 times the upper limit of normal
8. Creatinine <2.0 unless due to lymphoma. For creatinine clearance < 50 mL/min due to kidney involvement by tumor see Section 7.3.4 for chemotherapy adjustment.
9. KPS at least 50 (Appendix I).
10. Age at least 18.
11. Voluntary written informed consent before performance of any study-related procedure not part of normal medical care, with the understanding that consent may be withdrawn by the subject at any time without prejudice to future medical care.
12. Female patients of child bearing potential must have a negative pregnancy test within 72 hrs of initiation of therapy. Female patients are either post-menopausal or surgically sterilized or willing to use two acceptable methods of birth control (i.e., a hormonal contraceptive, intra-uterine device, diaphragm with spermicide, condom with spermicide, or abstinence) during the study. Male patients must agree to use two acceptable methods for contraception for the duration of the study. Women must avoid pregnancy and men avoid fathering children while in the study.
13. HIV positive patients are eligible if they are receiving at least two other active anti-HIV therapies other than zidovudine or atazanavir.
14. Patients with active HBV infection are eligible if they are receiving effective anti-HBV therapy.
15. Inclusion of Women and Minorities: Both men and women and members of all races and ethnic groups are eligible for this trial.

3.2 Exclusion Criteria

Patients meeting any of the following exclusion criteria are not to be enrolled in the study.

1. Acute active infection requiring acute therapy. Chronic therapy with potentially myelosuppressive agents is allowed provided that entry hematologic criteria are met.
2. Diagnosed or treated for another malignancy within 3 years of enrollment, with the exception of complete resection of basal cell carcinoma or squamous cell carcinoma of the skin, an in situ malignancy, or low-risk prostate cancer after curative therapy.
3. Women who are pregnant or breastfeeding. Confirmation that the subject is not pregnant must be established by a negative serum β-human chorionic gonadotropin (β-hCG) pregnancy test result obtained during screening. Pregnancy testing is not required for post-menopausal or surgically sterilized women.
4. Patient has ≥Grade 2 peripheral neuropathy

5. Myocardial infarction within 6 months prior to enrollment or has New York Heart Association (NYHA) Class III or IV heart failure (see Appendix VI), uncontrolled angina, severe uncontrolled ventricular arrhythmias, or electrocardiographic evidence of acute ischemia or active conduction system abnormalities. Prior to study entry, any ECG abnormality at Screening has to be documented by the investigator as not medically relevant.
6. Patient has hypersensitivity to bortezomib, boron or mannitol.
7. Patient has received other investigational drugs with 14 days before enrollment
8. Serious medical or psychiatric illness likely to interfere with participation in this clinical study.
9. >1.5x ULN total bilirubin, except if is determined to be related to Gilbert's disease or tumor biliary/liver involvement.

4.0 REGISTRATION PROCEDURES

4.1 Enrollment/Registration Process

The study will be available to U.S. sites with IRB approval. After eligibility has been determined and an informed consent has been signed by the patient, they must be registered. Registration must occur prior to initiation of therapy (cycle 1 day 1).

Patients must not start any protocol intervention prior to registration through the Siteman Cancer Center.

The following steps must be taken before registering patients to this study:

1. Complete Siteman Cancer Center Patient Registration Form and fax to Project Coordinator 314-747-2120
2. Confirmation of patient eligibility by Washington University
3. Registration of patient in the Siteman Cancer Center database
4. Assignment of unique patient number (UPN)

Once the patient has been entered in the Siteman Cancer Center database, the Project Coordinator will forward verification of enrollment and the UPN via email.

4.2 Confirmation of Patient Eligibility

Confirm patient eligibility by scanning and emailing (or faxing) the information below to the Project coordinator. You may contact the Project Coordinator at 314-362-8836, Monday through Friday, 8am to 5pm, Central Standard Time. Email to lratner@dom.wustl.edu or fax the following information to 314-747-2120.

1. Your name, telephone, fax number and email address
2. Protocol version number and protocol version date
3. Your principal investigator's name, registering MD's name, and your institution name

4. Patient's race, sex, date of birth
5. Three letters (or two letters and a dash) for patient's initials
6. Date the informed consent was signed, and copy of signed consent form (patient's name and signature may be blacked out)
7. Planned date of enrollment
8. Completed and signed Eligibility Checklist for Protocol
9. Copy of appropriate source documentation confirming patient eligibility

The Project Coordinator will assign a study identification number to the patient. This study identification number is to be used on all data collection forms for the protocol. Confirmation of registration will be faxed to the registering institution.

4.3 Patient Registration in the Siteman Cancer Center Database

Registrations may be submitted Monday through Friday between 8am and 5pm CST. Urgent late afternoon or early morning enrollments should be planned in advance and coordinated with the Washington University Project Coordinator. Registration will be confirmed by the Project Coordinator or his/her delegate by email within one business day. Verification of eligibility and registration should be kept in the patient chart.

Patients at all sites must be registered through the Siteman Cancer Center database at Washington University.

4.4 Assignment of UPN

Each patient will be identified with a unique patient number (UPN) for this study. Patients will also be identified by first, middle, and last initials. If the patient has no middle initial, a dash will be used on the case report forms (CRFs). All data will be recorded with this identification number on the appropriate CRFs.

5.0 CLINICAL AND LABORATORY EVALUATIONS

5.1 Baseline/Pretreatment Evaluation (See Appendix IV)

The following will be obtained no more than 30 days prior to the initiation of therapy:

- Complete medical history to include:
    - Duration of HTLV-1 infection if known, birthplace, travel history, history of other complications of HTLV-1 infection.
    - Date of initial diagnosis of ATLL. A copy of the pathology report must be available in the medical record. Central pathological review is required within 30 days of study initiation. (Appendix III).
    - Presence or absence of "B"-symptoms (unexplained fevers, night sweats, involuntary weight loss greater than 10% normal body weight).
    - History of other symptoms related to ATLL or HAM.
    - History of drug allergies.

- Medication list to include all antivirals, antibiotics, and prophylactic medications.
- For HIV+, date of diagnosis of HIV.

- Complete physical examination to include:
  - Karnofsky performance score (see Appendix I)
  - vital signs
  - weight
  - height
  - body surface area
  - neurologic examination including the neurotoxicity questionnaire (Appendix VII)
  - careful measurement of all palpable peripheral lymph nodes
  - measurement of other sites of disease present on physical examination.
- EKG and ECHO or MUGA to assess LV ejection fraction
- Urinalysis.
- Laboratory tests:
  - HTLV-1 serology by ELISA, confirmed by RIPA, PCR, or Western blot
  - (GeneLabs Diagnostics, HTLV Blot 2.4).
  - Hematology: CBC, platelet count, and differential. CBC and differential will be repeated on Days, 8, 10, 12, and 15 (up to -4 days). If by the second CBC, the nadir has been reached and the ANC is recovering, no further CBCs are necessary (i.e. 2 CBCs will suffice). will be performed on days 1, 8, 10, and 12 to determine initial and nadir CBC for each cycle.
  - Blood chemistries: to include sodium, potassium, chloride, CO2, creatinine, calcium, phosphorus, uric acid, total bilirubin, AST, ALT, alkaline phosphatase, total protein, albumin, LDH.
  - T cell subsets (CD3, CD4, CD8, and CD25).
  - Specimens sent for HTLV-1 DNA proviral load, RNA, protein expression, HTLV-1 integrase gene sequence, integration site, and gene array (50 ml yellow top) (See Appendix VIII).
  - Serum pregnancy test for women of childbearing age.
- Staging Evaluation: The following studies will be done for baseline evaluation of extent of disease. The Ann Arbor staging classification will be used (See Appendix II).
  - PET/CT scan of the chest, abdomen, and pelvis (CT scans alone are appropriate at centers that do not have PET scans).
  - Bone marrow aspirate and biopsy (single core, at least 2.0 cm). Bone marrows need to be repeated only if positive at baseline unless peripheral blood involvement is unequivocally demonstrated by flow cytometry studies.
  - Lumbar puncture with routine studies and cytology. Lumbar puncture should be deferred until after completion of cycle 1 therapy if patient has leukemia at presentation.

5.2 Evaluations During Chemotherapy Treatment (See Appendix IV).

- Medical History, including Neurotoxicity Questionnaire (Appendix VII)
- Physical examination including, vital signs, Karnofsky performance score will be repeated on day 1 (up to -4 days) of each chemotherapy cycle. Disease measurable on physical examination should be measured in two dimensions.
- CBC and differential will be repeated at the start of each chemotherapy cycle, on Days 1, and also on Days, 8, 10, 12, and 15 (up to -4 days). If by the second CBC, the nadir has been reached and the ANC is recovering, no further DBCs are necessary (i.e. 2 CBCs will suffice).
- Electrolytes, creatinine, BUN, liver function tests (including total bilirubin, alkaline phosphatase, AST, ALT) and LDH will be obtained at the start of each chemotherapy cycle (up to -4 days).
- HTLV-1 DNA proviral load, HTLV-1 RNA, and integration sites will be repeated on day 1 (up to -4 days) of cycle 2 and then each subsequent odd numbered chemotherapy cycle (30 ml in yellow top tube) (see Appendix VIII).
- T cell counts (CD3, CD4, CD8, and CD25) will be repeated at the beginning of each odd numbered cycle (up to -4 days) of chemotherapy and at the conclusion of chemotherapy.
- Restaging evaluation (CT or MRI, other studies as indicated) of all initial sites of measurable disease will be performed following completion of cycle 2 and cycle 4 (if cycles 3 and 4 given) and cycle 6 (if cycles 5 and 6 given) of chemotherapy. CT or MRI's may be performed up to Day-7 before next cycle.
- Bone marrow biopsy should be performed after cycle 2 and cycle 4 (if cycles 3 and 4 given) and cycle 6 of chemotherapy (if cycles 5 and 6 given) if positive at baseline unless peripheral blood involvements is unequivocally demonstrated by flow cytometry studies.

5.3 Post-Treatment Evaluation (See Appendix IV)

Patients will be seen every 2 months (+/- 7 days) and have a history and physical exam including vital signs and Karnofsky's performance status and CBC and serum chemistries for one year after completion of antiviral therapy, then every 3 months (+/- 7 days) for one year, and then every 6 months (+/- 14 days) for three additional years, to document survival, performance status, and duration of response. FACT/GOG-Neurotoxicity Questionnaire will be completed at the first follow-up visit only. CT or MRI scans will be repeated every 4 months (+/- 14 days) during the first year of follow-up. HTLV-1 DNA proviral load, HTLV-1 RNA, and integration sites will be repeated every 4 months (+/- 14 days) (30 ml in yellow top tubes), (see Appendix VIII).

5.4 Early Discontinuation of Therapy

Patients going off study prior to completion of therapy will have a complete physical examination, and blood drawn for the following studies: CBC with differential and platelets, serum chemistries, calcium, T cell counts, HTLV-1 proviral load, HTLV-1 RNA, and integration sites (30 ml in yellow top tubes) (up to +7 days). At time of relapse, 50 ml in yellow top tubes is requested.

6.0 STUDY MEDICATIONS

6.1 Treatment

Bortezomib 1.0 mg/m^2 is infused on days 1 and 4

Etoposide 50 mg/m^2/d given as a continuous 96 hr IV infusion on days 1-4

Vincristine 0.4 mg/m^2/d given as a continuous 96 hrs IV infusion on day 1-4

Doxorubicin 10 mg/m^2/d given as a continuous 96 hrs IV infusion on days 1-4

Prednisone 60 mg/m^2/d given orally on days 1-5

Cyclophosphamide (day 5) 375 mg/m^2 given IV on day 5 over 30 min

Cycles 2-6:
If ANC nadir >500/mm3 and platelet nadir >25,000/mm3 in the previous cycle, then increase doses of etoposide, doxorubicin, and cyclophosphamide doses by 20%. If ANC nadir <500/mm3 on at least 3 measurements OR nadir platelet count less than 25,000/mm3 on at least 1 measurement during the previous cycle, then decrease etoposide, doxorubicin, and cyclophosphamide doses by 20%. Only cyclophosphamide doses are reduced at dose levels -1, -2 and -3.

Note: **Cycle 2**: Cyclophosphamide, etoposide, and doxorubicin doses can be escalated by 20% if the investigator feels grade 3-4 febrile neutropenia was precipitated by marrow infiltration by malignant cells

Cycle 3-6: The cyclophosphamide, etoposide, and doxorubicin doses should be escalated only if there is no previous episode in cycles 2-5 of grade 3-4 febrile neutropenia or any other toxicity that required a dose reduction of cyclophosphamide

Doses for doxorubicin, etoposide and cyclophosphamide will be based on measurements of the previous cycle ANC or platelet nadir whichever is lower **(i.e., twice weekly starting 3-4 days after completion of chemotherapy).**

If ANC ≥ 500/μL on all measurements:	↑ one dose level*
If ANC < 500/μL on 1 or 2 measurements (3-4 days apart):	maintain dose level
If ANC < 500/μL ≥ 3 measurements: (3-4 days apart):	↓ one dose level**
**OR**	
If platelet < 25,000/μL on ≥ 1 measurement:	↓ one dose level

* to a maximum of Dose Level 7.

** to a minimum of Dose Level –2. Recurrence of a toxicity requiring a dose reduction below Dose Level –2 will result in removal of patient from protocol therapy.

Dose adjustments for hematologic toxicity apply only to etoposide, doxorubicin and cyclophosphamide. Only cyclophosphamide is reduced in Dose Levels –1 through –3.

	Dose Levels									
Adjusted Agents	-3	-2	-1	**1**	2	3	4	5	6	7
Doxorubicin (mg/m^2/day)	10	10	10	**10**	12	14.4	17.3	20.7	24.8	29.8
Etoposide (mg/m^2/day)	50	50	50	**50**	60	72	86.4	103.7	124.4	149.3
Cyclophosphamide (mg/m^2)	192	240	300	**375**	450	540	648	778	933	1120
Non-Adjusted Agents										
Vincristine (mg/m^2))(No cap)	0.4	0.4	0.4	**0.4**	0.4	0.4	0.4	0.4	0.4	0.4
Prednisone (mg/m^2 qd)	60	60	60	**60**	60	60	60	60	60	60

Raltegravir is given orally 400 mg bid every day starting with cycle 2 therapy.
Note: See section 7.0 for dose modifications for hematololgic and non-hematologic toxicity.

Cycles will be repeated every 21-28 days for two cycles beyond best response, or a maximum of 6 cycles. Best response is the response achieved when 1 or more additional cycles of chemotherapy are given and no additional tumor shrinkage is noted. That may include stable or progressive disease after 2 cycles of chemotherapy.

Plus: Neulasta 6 mg SQ on days 6, 7, or 8 or G-CSF 5 ug/kg (rounded to nearest 300 or 480 mcg) SQ daily for 10 days starting on day 6 or until the absolute neutrophil account has recovered to >4000 cells/mm3

6.2 Bortezomib

Bortezomib is a proteasome inhibitor that blocks the degradation of several critical cellular proteins, including inhibitor of kappa B (IκB). It is 83% protein bound, metabolized in the liver via CYP2C19 and CYP3A4 and to a lesser extent CYP1A2 with a half-life of 9-15 hrs.

Bortezomib for Injection is a sterile lyophilized powder for reconstitution and is supplied in vials containing bortezomib and mannitol at a 1:10 ratio (3.5 mg of bortezomib and 35 mg of mannitol). Bortezomib is a modified dipeptidyl boronic acid. The product is provided as a mannitol boronic ester which, in reconstituted form, consists of the mannitol ester in equilibrium with its hydrolysis product, the monomeric boronic acid. The drug substance exists in its cyclic anhydride form as a trimeric boroxine.

6.2.1 Preparation, Reconstitution, and Dispensation

Detailed instructions for preparation of the study drug are provided in the drug labels for Bortezomib and in the pharmacy manual.

6.2.2 Bortezomib reconstitution

Drug is available in sterile, single-use vials containing 3.5 mg of bortezomib and 35 mg of mannitol. Each vial of bortezomib for Injection should be reconstituted in 3.5 mL of normal (0.9%) saline, Sodium Chloride for Injection, USP, within 8 hours prior to dosing, so that the reconstituted solution contains bortezomib at a concentration of 1 mg/mL. Dissolution is completed in approximately 10 seconds. The reconstituted solution is clear and colorless, with a final pH of 5 to 6. Reconstituted bortezomib should be administered promptly and in no case more than 8 hours after reconstitution.

Reconstitution should be performed inside a laminar flow biological cabinet (hood). Prior to reconstitution, bortezomib vials should remain in original packaging to protect them from light. All materials that have been used for drug preparation should be disposed of according to standard practices. A log must be kept of all disposed materials.

6.2.3 Packaging and Labeling

Bortezomib will be supplied in vials as open-label stock. Both the box label and vial label will fulfill all requirements specified by governing regulations.

Bortezomib will be provided in labeled cartons, each containing 10 labeled vials. Each label will contain the lot number and expiration date of the drug.

6.2.4 Administration

Drug will be administered only to eligible patients under the supervision of the investigator or identified sub-investigator(s). Patients may be treated on an out-patient basis, if possible.

The pharmacist will prepare the drug under aseptic conditions. The amount (in mg) of drug to be administered will be determined based on body surface area. Body surface area is to be calculated based on body weight using a standard nomogram (see Appendix V). The dose should be calculated on Day 1 of each cycle; the dose administered should remain the same throughout each cycle but should be recalculated

at the start of the next cycle. If a patient experiences a notable change in weight (eg, loss or gain of ≥8 lbs or 3.6 kg) within a cycle, as determined by an unscheduled weight assessment, then the patient's dose should be recalculated at that time.

The appropriate amount of bortezomib will be drawn from the injection vial and administered as an intravenous (IV) push over 3 to 5 seconds followed by a standard saline flush or through a running IV line. Vials are for single use administration.

6.2.5 Bortezomib Return

For commercially-labeled bortezomib for IND-exempt studies, please contact your Millennium Clinical Operations representative to arrange for return of study drug procedures. Any unused or expired bortezomib must be returned to Millennium. Be sure to document drug return on your drug accountability logs.

6.2.6 Storage, Handling, and Accountability

All investigational supplies are to be kept in a secure area with controlled access.

A drug dispensing log, including records of drug received from the sponsor and drug dispensed to the subjects, will be provided and kept at the study site.

Vials containing lyophilized bortezomib for Injection should be stored according to the label requirements. For the US, store at USP Controlled Room Temperature which is 25ºC (77ºF); excursions permitted from 15ºC to 30ºC (59−86ºF). To date, stability data indicate that the lyophilized drug product is stable for at least 18 months when stored under the recommended conditions. Stability studies are ongoing, and Millennium Pharmaceuticals, Inc. will notify the investigator should this information be revised during the conduct of the study.

Bortezomib is cytotoxic. As with all cytotoxic drugs, caution is required when preparing and handling bortezomib solutions. Cytotoxic drugs should only be handled by staff specially trained in the safe handling of such preparations. The use of gloves and other appropriate protective clothing is recommended. In case of skin contact, wash the affected area immediately and thoroughly with soap and water for at least 15 minutes. If product contacts eye, immediately flush eye thoroughly with water for at least 15 minutes. Always contact a physician after any form of body contact. All materials that have been used for preparation should be disposed of according to standard practices. A log must be kept of all disposed materials.

6.3 Chemotherapy Medications

6.3.1 Etoposide

Etoposide or VP-16 is an epipodophyllotoxin derived from the mandrake plant Podophyllum peltatum. It is a cell cycle phase specific agent that blocks topoisomerase II. It has a biphasic half-life and is eliminated by both renal clearance and metabolism.

6.3.1.1 Preparation, Reconstitution, and Dispensation

Etoposide injection contains polysorbate 80 which may cause leaching of diethylhexyl phthalate (DEHP), a plasticizer contained in polyvinyl chloride (PVC) bags and tubing. Higher concentrations and longer storage time after preparation in PVC bags may increase DEHP leaching. Preparation in glass or polyolefin containers will minimize patient exposure to DEHP. Etoposide should be diluted to a concentration of 0.2-0.4 mg/mL in NS for administration. If the etoposide dose is ≤150 mg/day, then place in 500 mL NS. If the etoposide dose is >150 mg/day, place in 1000 mL, NS.

Diluted solutions have concentration-dependent stability: More concentrated solutions have shorter stability times. Precipitation may occur with concentrations >0.4 mg/mL. Reconstitution should be performed inside a laminar flow biological cabinet (hood). All materials that have been used for drug preparation shuld be disposed of according to standard practice. A log must be kept of all disposed materials.

6.3.1.2 Packaging and Labeling

Injection, solution: 20 mg/mL (5 mL, 25 mL, 50 mL) [contains benzyl alcohol, ethanol 30.5%, polyethylene glycol 300, and polysorbate 80]. Each label will contain the lot number and expiration date of the drug.

6.3.1.3 Administration

Concentrations >0.4 mg/mL are very unstable and may precipitate within a few minutes. For large doses, where dilution to ≤0.4 mg/mL is not feasible, consideration should be given to slow infusion of the undiluted drug through a running normal saline, dextrose or saline/dextrose infusion; or use of etoposide phosphate. Etoposide solutions of 0.1-0.4 mg/mL may be filtered through a 0.22 micron filter without damage to the filter or significant loss of drug.

6.3.1.4 Storage, Handling, and Accountability

Store intact vials of injection at 15°C to 30°C (59°F to 86°F). Protect from light. Solutions for infusion, at room temperature, in D_5W or NS in polyvinyl chloride, the concentration is stable as follows:

0.2 mg/mL: 96 hours
0.4 mg/mL: 24 hours

Cytotoxic drugs should only be handled by staff specially trained in the safe handling of such preparations. The use of gloves and other appropriate protective clothing is recommended. In case of skin contact, wash the affected area immediately and thoroughly with soap and water for at least 15 minutes. If product contacts eye,

immediately flush eye thoroughly with water for at least 15 minutes. Always contact a physician after any form of body contact. All materials that have been used for preparation should be disposed of according to standard practices. A log must be kept of all disposed materials.

6.3.1.5 Side-effects

Likely: Nausea and vomiting, hair loss, weakness, tiredness, leucopenia, anemia

Less Likely: Anorexia, decreased blood pressure during the infusion, rashes, diarrhea, pain in the abdomen, mouth sores, neuropathy, extreme tiredness or weakness, loosening of the finger or toe nails, phlebitis at infusion site, chest pain

Rare: Damage to the liver, anaphylactoid reactions, low blood pressure, tachycardia, chills and fever, a new cancer or leukemia resulting from this treatment, severe rashes with skin sloughing, amenorrhea, infertility, cardiomyopathy.

6.3.2 Vincristine Sulfate

Vincristine sulfate is a vinca alkaloid from the plant Cantharanthus roseus. It acts by binding to or crystallizing microtubular proteins of the mitotic spindle. It is a cell cycle phase specific agent and can also affect DNA directed RNA polymerase. It has a triphasic half-life and primary elimination is by the liver into the bile and feces. The major and dose-limiting side effect of vincristine is neurotoxicity.

6.3.2.1 Preparation, Reconstitution, and Dispensation

Injection, solution, as sulfate [preservative free]: Vincasar PFS®: 1 mg/mL (1 mL, 2 mL). I.V. solution: Diluted in 20-50 mL NS or D_5W, stable for 7 days under refrigeration, or 2 days at room temperature. In ambulatory pumps, solution is stable for 7-10 days at room temperature. Solutions for I.V. infusion may be mixed in NS or D_5W. **Note:** The World Health Organization recommends dispensing vincristine in a minibag, rather than a syringe. Reconstitution should be performed inside a laminar flow biological cabinet (hood). All materials that have been used for drug preparation shuld be disposed of according to standard practice. A log must be kept of all disposed materials.

6.3.2.2 Packaging and Labeling

Each label will contain the lot number and expiration date of the drug.

6.3.2.3 Administration

Vesicant. For I.V. use only. **Fatal if given intrathecally.** Usually administered as short (10-15 minutes) infusion (preferred) or slow (1-2 minutes) push; 24-hour continuous infusions are occasionally used. Follow guidelines for handling cytotoxic agents. Drug should be administered by qualified personnel. Do not allow to come in contact with

skin. If contact occurs, wash thoroughly with soap and water. Avoid extravasation; agent is a vesicant and will cause sloughing.
pH: 3.5-5.5

6.3.2.4 Storage, Handling, and Accountability

Undiluted vials: Store under refrigeration. May be stable for up to 30 days at room temperature. Cytotoxic drugs should only be handled by staff specially trained in the safe handling of such preparations. The use of gloves and other appropriate protective clothing is recommended. In case of skin contact, wash the affected area immediately and thoroughly with soap and water for at least 15 minutes. If product contacts eye, immediately flush eye thoroughly with water for at least 15 minutes. Always contact a physician after any form of body contact. All materials that have been used for preparation should be disposed of according to standard practices. A log must be kept of all disposed materials

6.3.2.5 Side-effects

Likely: Hair loss, reversible neuropathy, constipation.

Less Likely: Jaw pain, headache, muscle weakness, abdominal pain and bloating, neuropathy, wrist or foot drop, drooping eyelids, double vision, difficulty seeing at night, hoarseness, dysphagia, difficulty with urination dizziness, SIADH, leucopenia, anemia, thrombocytopenia

Rare: Ileus, if drug extravasates it will cause damage to nearby tissue, seizures, vocal cord paralysis, difficulty breathing, inability to walk, decreased hearing, optic nerve damage, damage to the liver which can lead to inflammation and/or scarring which could lead to jaundice, ascites.

6.3.3 Doxorubicin

Doxorubicin is an anthracycline antibiotic which binds tightly with DNA, inhibits nucleic acid synthesis and causes DNA strand breaks. Although active throughout the cell cycle, cells in S phase are most sensitive.

6.3.3.1 Preparation, Reconstitution, and Dispensation

Reconstitute lyophilized powder with NS to a final concentration of 2 mg/mL (may further dilute in 50-1000 mL D_5W or NS for infusion). Unstable in solutions with a pH <3 or >7. Reconstitution should be performed inside a laminar flow biological cabinet (hood). All materials that have been used for drug preparation shuld be disposed of according to standard practice. A log must be kept of all disposed materials.

6.3.3.2 Packaging and Labeling

Injection, powder for reconstitution, as hydrochloride: 10 mg, 50 mg
Adriamycin®: 10 mg, 20 mg, 50 mg, [contains lactose]
Injection, solution, as hydrochloride: 2 mg/mL (5 mL, 10 mL, 25 mL, 100 mL)
Adriamycin®: 2 mg/mL (5 mL, 10 mL, 25 mL, 100 mL)
Each label will contain the lot number and expiration date of the drug.

6.3.3.3 Administration

Vesicant. I.V. push over at least 3-5 minutes or IVPB over 15-60 minutes. Infusion via central venous line recommended. May be further diluted in either NS of D_5W for I.V. administration. Avoid extravasation associated with severe ulceration and soft tissue necrosis. Flush with 5-10 mL of I.V. solution before and after drug administration. Incompatible with heparin. Monitor for local erythematous streaking along vein and/or facial flushing (may indicate rapid infusion rate). pH: 3.8-6.5 (lyophilized doxorubicin HCl reconstituted with sodium chloride 0.9%); 2.5-4.5 (adjusted solution)

6.3.3.4 Storage, Handling, and Accountability

Store intact vials of solution under refrigeration at 2°C to 8°C. Protected from light. Store intact vials of lyophilized powder at room temperature (15°C to 30°C). Reconstituted vials are stable for 7 days at room temperature (25°C) and 15 days under refrigeration (5°C) when protected from light. Infusions are stable for 48 hours at room temperature (25°C) when protected from light. Solutions diluted in 50-1000 mL D_5W or NS are stable for 48 hours at room temperature (25°C) when protected from light. Cytotoxic drugs should only be handled by staff specially trained in the safe handling of such preparations. The use of gloves and other appropriate protective clothing is recommended. In case of skin contact, wash the affected area immediately and thoroughly with soap and water for at least 15 minutes. If product contacts eye, immediately flush eye thoroughly with water for at least 15 minutes. Always contact a physician after any form of body contact. All materials that have been used for preparation should be disposed of according to standard practices. A log must be kept of all disposed materials

6.3.3.5 Side-effects

Likely: Nausea, vomiting, temporary hair loss, pink or red color to urine, sweat, tears and saliva, leukopenia, anemia, thrombocytopenia, decreased cardiac ejection fraction

Less Likely: Sores in mouth and esophagus, dysphagia, cardiomyopathy, facial flushing, fever/chills, hives, high levels of uric acid in the blood which can cause renal insufficiency, discoloration of the hands, feet and under the fingernails with possible separation of the nail from the nail bed, extravasation injury, phlebitis, increased liver enzymes, conjunctivitis, radiation recall, diarrhea

Rare: Severe allergic reaction which can be life-threatening with shortness of breath, low blood, pressure and a rapid heart rate, ulceration of the lower intestinal tract, an

irregular heart beat which can be life-threatening, severe cardiomyopathy, new cancer or leukemia resulting from this treatment.

6.3.4 Cyclophosphamide

Cyclophosphamide is an alkylating agent and is cell cycle nonspecific. It causes cross linking of DNA and is the most active single agent in the treatment of non-Hodgkin's lymphoma.

6.3.4.1 Preparation, Reconstitution, and Dispensation

Injection, powder for reconstitution: 500 mg, 1 g, 2 g. Reconstitute vials with 250 ml NS. Reconstitution should be performed inside a laminar flow biological cabinet (hood). All materials that have been used for drug preparation shuld be disposed of according to standard practice. A log must be kept of all disposed materials.

6.3.4.2 Packaging and Labeling

Each label will contain the lot number and expiration date of the drug.

6.3.4.3 Administration

I.V. infusions are administered over 30 min. To minimize bladder toxicity, increase normal fluid intake during and for 1-2 days after cyclophosphamide dose. Most adult patients will require a fluid intake of at least 2 L/day. High-dose regimens should be accompanied by vigorous hydration with or without mesna therapy.

6.3.4.4 Storage, Handling, and Accountability

Store intact vials of powder at room temperature of 15°C to 30°C (59°F to 86°F). Reconstituted solutions are stable for 24 hours at room temperature and 6 days under refrigeration 2°C to 8°C (36°F to 46°F). Further dilutions in D_5W or NS are stable for 24 hours at room temperature (25°C) and 6 days at refrigeration.

Cytotoxic drugs should only be handled by staff specially trained in the safe handling of such preparations. The use of gloves and other appropriate protective clothing is recommended. In case of skin contact, wash the affected area immediately and thoroughly with soap and water for at least 15 minutes. If product contacts eye, immediately flush eye thoroughly with water for at least 15 minutes. Always contact a physician after any form of body contact. All materials that have been used for preparation should be disposed of according to standard practices. A log must be kept of all disposed materials

6.3.4.5 Side Effects

Likely: Loss of appetite, nausea, vomiting, leucopenia, hair loss, decrease in sperm count

Less-likely: SIADH, abdominal pain, diarrhea, anemia, thrombocytopenia, hemorrhagia cystitis, amenorrhea, infertility, temporary blurred vision, nasal stuffiness with IV infusions, skin rash, darkening of areas of the skin and finger nails, slow healing of wounds and infections.

Rare: Cardiomyopathy may occur with very high doses and which may be fatal, abnormal heart rhythms, pneumonitis, new cancer or leukemia resulting from this treatment, damage or scarring of urinary bladder tissue, severe allergic reaction which can be life-threatening, infertility.

6.3.5 Prednisone

Prednisone is a corticosteroid and its mechanism of action as a cytotoxic agent is not clearly understood.

6.3.5.1 Preparation, Reconstitution, and Dispensation

Solution, oral: 1 mg/mL (5 mL, 120 mL, 500 mL) [contains alcohol 5%, sodium benzoate; peppermint vanilla flavor]
Solution, oral [concentrate]: PredniSONE Intensol™: 5 mg/mL (30 mL) [contains alcohol 30%]
Tablet: 1 mg, 2.5 mg, 5 mg, 10 mg, 20 mg, 50 mg

6.3.5.2 Packaging and Labeling

Each vial will contain a lot number and expiration date of the drug.

6.3.5.3 Administration

Administer with food to decrease GI upset. Should be taken after meals or with food or milk; may require increased dietary intake of pyridoxine, vitamin C, vitamin D, folate, calcium, and phosphorus; may require decreased dietary intake of sodium

6.3.5.4 Storage, Handling, and Accountability

Drug should only be handled by staff specially trained in the safe handling of such preparations.

6.3.5.5 Side-effects

Likely: High blood pressure, swelling, headache, sores in mouth or stomach, hyperglycemia, myopathy, vascular fragility and bruising, increase in appetite and nausea, weight gain, high risk of infections, mood changes, difficulty sleeping.

Less Likely: Changes in appetite or mood including feelings of elation or depression, nausea, sodium retention, impaired skin healing, increased risk of infection, osteoporosis and hyperglycemia.

Rare: Glaucoma and/or cataracts, Addison's disease, myopathy, blood clots, acne, increased hair growth, roundness of the face and allergic reaction

6.4 Antiviral Medication

Raltegravir is a diketone integrase inhibitor approved for treatment of HIV-1 infection in combination with other antiretroviral agents in treatment-experienced adult patients who have evidence of viral replication and HIV-1 strains resistant to multiple antiretroviral agents.

6.4.1 Preparation, Reconstitution, and Dispensation

Tablet: Isentress®: 400 mg

6.4.2 Packaging and Labeling

Ralgetravir will be supplied and labeled stock for investigational use. The labeling fulfills all requirements specified by governing regulations.

Raltegravir will be provided in labeled cartons, each containing labeled vials. Each label will contain the lot number and expiration date of the drug.

6.4.3 Administration

May be taken with or without food.

6.4.4 Storage, Handling, and Accountability

Store at room temperature of 20°C to 25°C (68°F to 77°F); excursions permitted to 15°C to 30°C (59°F to 86°F). All investigational supplies are to be kept in a secure area with controlled access.
A drug dispensing log, including records of drug received from the sponsor and drug dispensed to the subjects, will be provided and kept at the study site.

6.4.5 Side-effects

Likely: None known

Less likely: Headache, fatigue, dizziness, itching, glucose increased, loss of body fat, vomiting, increased in the enzymes amylase and lipase, abdominal pain, increase in liver function with jaundice, increase CPK.

Rare: Leukopenia, anemia, myocardial infarction, hepatitis, herpes simplex and kidney damage or failure.

6.5 Concurrent Medication

6.5.1 Antiretroviral Therapy

For patients receiving antiretroviral therapy, prior to study initiation, for HIV HBV, or HCV infection or other indication, these agents (other than zidovudine or atazanavir or interferon) will be continued. HIV infected patients must be receiving at least two additional active anti-HIV medications.

6.5.2 Pneumocystis Carinii Prophylaxis

All patients will receive prophylaxis with either co-trimoxazole or dapsone.

6.5.3 Prevention of Tumor Lysis Syndrome (Cycle 1 only)

It is recommended that patients with evidence of high number of circulating tumor cells (>50,000/ml) or high tumor burden (bone marrow involvement, LDH>500) receive allopurinol, 600 mg 24 hours prior to the initiation of chemotherapy followed by 300 mg daily to continue for at least 7 days following administration of the first cycle of chemotherapy. If allopurinol was initiated at any earlier date, it should be given at 300 mg daily as described above, and then 600 mg dose may be omitted. Additional

measures such as aggressive IV hydration and urinary alkalinization will be used at the discretion of the investigator.

6.5.4 Anti-Infectives

All appropriate anti-infectives may be administered as clinically indicated.

6.5.5 Herpes Zoster Prophylaxis

All patients will receive prophylaxis with acyclovir or valacyclovir to prevent herpes zoster as a result of bortezomib treatment.

6.5.6 Prohibited Concurrent Medication

Any investigational agent other than bortezomib.or raltegravir

6.6 CNS Prophylaxis

CNS prophylaxis will be left to the discretion of the investigator, but it is recommended that either of the following two regimens be provided:

a. Cytosine arabinoside 50 mg administered intrathecally on days 1, 8, 15, and 22 of the first chemotherapy cycle. Please refer to the approved package insert for complete prescribing and toxicity information.

b. Methotrexate 12 mg administered intrathecally on days 1, 8, 15, and 22 of chemotherapy cycle 1. Please refer to the approved package insert for complete prescribing and toxicity information.

6.7 Meningeal Lymphoma

Patients with positive CSF cytology will receive treatment as follows: AraC, 50 mg in preservative-free saline will be instilled using an intraventicular reservoir two-three times weekly until CSF cytology normalizes, then once weekly for 4 weeks, then on alternate weeks for 4 doses, then monthly for a total duration of intrathecal therapy of one year. Intrathecal administration should be given only if platelets are >25,000/mm3. Alternatively, liposomal AraC 50 mg every 14 days for 2 doses for induction, every 14 days for 3 doses for consolidation, then every 28 days for 5 doses for maintenance. Liposomal AraC should be given with 5 days of dexamethasone (4 mg po bid) beginning at least 1 hr before liposomal AraC.

6.8 Radiotherapy

Whole brain radiotherapy is recommended for patients who present with neurologic signs and symptoms. It should be administered as soon as possible after diagnosis according to a standard protocol at each institution.

6.9 Treatment Compliance

All drug will be administered to eligible patients under the supervision of the investigator or identified sub-investigator(s). The pharmacist will maintain records of drug receipt (if applicable), drug preparation, and dispensing, including the applicable lot numbers, patients' height, body weight, and body surface area (see Appendix V), and total drug administered in milliliters and milligrams. Any discrepancy between the calculated dose and dose administered and the reason for the discrepancy must be recorded in the source documents.

7.0 DOSE MODIFICATION AND DELAY/TOXICITY MANAGEMENT

7.1 Bortezomib Modifications

Dose escalation will not be allowed in any patient, and there must be at least 72 hours between each dose of bortezomib.

Before each drug dose, the patient will be evaluated for possible toxicities that may have occurred after the previous dose(s). Toxicities are to be assessed according to the NCI Common Terminology Criteria for Adverse Events (CTCAE), Version 3.0.

Table 7-1 Management of Patients with Bortezomib Related Neuropathic Pain and/or Peripheral Sensory or Motor Neuropathy

**Recommended Dose Modification for Bortezomib related Neuropathic Pain and/or Peripheral Sensory or Motor Neuropathy**	
**Severity of Peripheral Neuropathy Signs and Symptoms**	**Modification of Dose and Regimen**
Grade 1 (paresthesias, weakness and/or loss of reflexes) without pain or loss of function	No action
Grade 1 with pain or Grade 2 (interfering with function but not with activities of daily living)	Reduce bortezomib to 0.7 mg/m^2
Grade 2 with pain or Grade 3 (interfering with activities of daily living)	Withhold* bortezomib therapy until toxicity resolves.<br>When toxicity resolves reinitiate with a reduced dose of bortezomib reduced by 30% and change treatment schedule to once per week.*
Grade 4 (Sensory neuropathy which is disabling or motor neuropathy that is life threatening or leads to paralysis)	Discontinue bortezomib
Grading based on NCI Common Terminology Criteria CTCAE v3.0<br>NCI Common Terminology Criteria website - http://ctep.info.nih.gov/reporting/ctc.html	

ADL = activities of daily living

*Key:

Reduce by one dose level: bortezomib dose reduction from 1.0 to 0.7 mg/m^2/dose.

Hold: Interrupt bortezomib for up to 2 weeks until the toxicity returns to Grade 1 or better.

Schedule change: Schedule change from bortezomib twice per week (Days 1 and 4 on a Q3W cycle) to once per week (Days 1 only on a Q3W cycle). If the treatment schedule is already once weekly, then it should remain once weekly.

The neurotoxicity-directed questionnaire (see Appendix VII) is a useful tool for determining the presence and intensity of neuropathic pain and/or peripheral neuropathy from the patient's perspective. Neuropathic symptoms are more prominent than abnormalities on the clinical examination. After the patient completes the neurotoxicity-directed questionnaire, the questionnaire should be reviewed to assist with the evaluation of the onset and intensity of peripheral neuropathy and other neurotoxicities that may possibly require intervention or dose modification.

- For hematologic toxicities, bortezomib is to be held for up to 2 weeks until the patient has a hemoglobin value of 8.0, platelet count >75,000cells/mm3 and ANC>1000 cells/mm3. If 2 or more consecutive bortezomib doses are withheld reduce dose level from 1.0 to 0.7 mg/m^2/dose.
- For nonhematological toxicitities withhold bortezomib until toxicity resolves to grade 1 or baseline. May reinitiate bortezomib at 0.7 mg/m^2/dose.
- If there is neuropathy interfering with function, reduce dose to 0.7 mg/m^2/dose. If painful grade 2 or grade 3 neuropathy interfering with daily level, withhold bortezomib until toxicity resolves, and then reinitiate at 0.7 mg/m^2 once weekly on weeks 1 and 2 of chemotherapy cycles. If grade 4 neuropathy, discontinue bortezomib.

All previously established or new toxicities observed any time, with the exception of neuropathic pain and peripheral sensory neuropathy, are to be managed as follows:

If the patient experiences febrile neutropenia, a Grade 4 hematologic toxicity (including a platelet count $<25 \times 10^9$/L) or any ≥Grade 3 non-hematologic toxicity considered by the investigator to be related to bortezomib, then drug is to be held.

Dose interruption or study discontinuation is **not** required for lymphopenia of any grade.

If, after bortezomib has been held, the toxicity does not resolve, as defined above, then drug must be discontinued.

If the toxicity resolves, as defined above, and bortezomib is to be restarted, the dose must be reduced by approximately 30% as follows:

- If the patient was receiving 1.0 mg/m^2, reduce the dose to 0.7 mg/m^2.
- If the patient was receiving 0.7 mg/m^2, discontinue drug, unless patient is responding, in which case this should be discussed with the PI. Dose reductions below 0.7 mg/m^2 should be avoided, but will be considered if the patient is having a good response or hepatic impairment is present.

Patients who experience bortezomib-related neuropathic pain and/or peripheral sensory neuropathy are to be managed as presented in Table 7-1 Management of Patients with Bortezomib-Related Neuropathic Pain and/or Peripheral Sensory Neuropathy.

7.1.1 Dosage in Patients with Hepatic Impairment

Patients with mild hepatic impairment do not require a starting dose adjustment and should be treated per the recommended bortezomib dose. Patients with moderate or severe hepatic impairment should be started on bortezomib at a reduced dose of 0.7 mg/m^2 per injection during the first cycle, and a subsequent dose escalation to 1.0 mg/m^2 or further dose reduction to 0.5 mg/m^2 may be considered based on patient tolerance (see Table 7-2).

Table 7-2: Recommended Starting Dose Modification for Bortezomib in Patients with Hepatic Impairment

SGOT (AST) Levels	Modification of Starting Dose	
≤ 1.0x ULN	> ULN	None
> 1.0x−1.5x ULN	Any	None
> 1.5x−3x ULN	Any	Reduce bortezomib to 0.7 mg/m^{2}in the first cycle. Consider dose escalation to 1.0 mg/m^2 or further dose reduction to 0.5 mg/m^2 in subsequent cycles based on patient tolerability.
> 3x ULN	Any	

7.2 Chemotherapy Modifications: Hematologic Toxicity

If ANC<1000/mm3 on day 1 of any treatment cycle, the next cycle will be delayed one week.

If after one week of delay, ANC>1000 with platelets >75,000, full dose therapy will be administered.

If ANC has not returned >1000 with platelets>75,000 after one week delay, drug doses will be modified on day 29 of the treatment cycle as follows:

PLATELETS	ABSOLUTE NEUTROPHIL COUNT		
	>1000/mm3	500-1000/mm3	<500/mm3
>75 K	Full dose	50% cyclophosphamide<br>50% doxorubicin<br>50% etoposide	Hold treatment
50-75K	50% cyclophospamide<br>50% doxorubicin<br>50% etoposide	50% cyclophospamide<br>50% doxorubicin<br>50% etoposide	Hold treatment
<50 K	Hold treatment	Hold treatment	Hold treatment

Patients unable to receive chemotherapy after a one-week delay will be followed with weekly CBC. If ANC>500 and platelets>50,000, these patients will be treated with 50% doses of cyclophosphamide, doxorubicin, and etoposide.

If ANC has not returned to >500 and platelets>50,000 by day 43, the patient will be withdrawn from the study.

Dose adjustments for nadir ANC and platelet count on previous cycle are made as follows:

Nadir Measurement	Dose Adjustment
If Nadir ANC at least 500/mm3	20% increase in etoposide, doxorubicin, and cyclophopshamide above last cycle
If Nadir ANC<500/mm3 on 1 or 2 measurements	Same doses as last cycles
If Nadir ANC<500/mm3 on at least 3 measurements OR<br>Nadir platelet counts <25,000/mm3 on 1 measurement	20% decrease in etoposide, doxorubicin, and cyclophosphamide below last cycle

However, only cyclophosphamide dose is reduced at dose levels -1, -2 and -3.

7.3 Chemotherapy Modifications: Non-Hematologic Toxicity

Any patients experiencing any grade 4 toxicity other than hematologic or infection will have no further therapy on this protocol.

7.3.1 Dose adjustment for hyperbilirubinemia

- Bilirubin 1.2 to 3.0 mg/dl, reduce doxorubicin and vincristine doses by 50%.
- Bilirubin 3.1-5.0 mg/dl, reduce doxorubicin and vincristine doses to 25% of full dose.
- Bilirubin >5.0 mg/dl, doxorubicin and vincristine should not be administered.
- If bilirubin>3.0 mg/dl for more than 30 days and is not associated with the presence of hepatic involvement with ATLL, the patient will be removed from the study.

7.3.2 Dose Adjustment for Cardiac Toxicity

If clinical findings suggesting congestive heart failure are present, doxorubicin will be discontinued and evaluation by MUGA or 2-D ECHO will be performed.

7.3.3 Dose Adjustment for Neutrotoxicity

Vincristine will be discontinued permanently for peripheral neuropathy of grade 3 or more

7.3.4 Dose Adjustment for Renal Impairment

For Creatinine Clearance < 50cc/min

Drug	Reduce by
Etoposide	25% based on full dose initially or previous dose for cycles 2-6

7.4 Antiviral Toxicity Management

If any grade 3 or greater toxicity (except anemia or lymphopenia), hold Raltegravir until grade 1 or less, and then restart at same dose.

8.0 CRITERIA FOR TREATMENTAND/OR STUDY PARTICIPATION DISCONTINUATION

8.1 Patient Withdrawal

Patients will be informed that they have the right to withdraw from the study at any time for any reason, without prejudice to their medical care. The investigator also has the right to withdraw patients from the study for any of the following reasons:

- Patients developing a life threatening infection who are in the chemotherapy portion of the protocol will have chemotherapy interrupted until the infectious process has cleared. The subject will be withdrawn from the study treatment only if chemotherapy has been held for more than six weeks.
- Chemotherapy delays for more than six weeks, for any reason.
- Severe toxicities or occurrence of an unacceptable adverse event as determined by the Principal Investigator
- Progressive ATLL after at least 2 cycles of chemotherapy and 1 cycle of antiviral therapy.
- The investigator has the right to remove subjects from study for clinical reasons which he or she believes to be life threatening or resulting in significant morbidity to the subject.
- Major protocol violation.
- Pregnancy.
- Lost to follow-up.
- Siteman Cancer Center closes the study.
- Intercurrent illness
- A treatment cycle delay or bortezomib interruption of >2 weeks or missing three of four bortezomib doses within a treatment cycle because of toxicity
- Patient request
- Non-compliance

- Administrative reasons
- General or specific changes in the patient's condition unacceptable for further treatment in the judgment of the investigator

At the time of withdrawal, all study procedures outlined for the End of Study visit should be completed. The primary reason for a patient's withdrawal from the study is to be recorded in the source documents.

9.0 REGULATORY AND REPORTING REQUIREMENTS

9.1 Multi-center Sites Timeframe for Reporting Adverse Events

Reportable adverse events will be tracked for 30 days following the last day of study treatment.

All multi-center sites are to report all reportable events electronically (via email) to the Protocol Chair, Lee Ratner, M.D., Ph.D. according to the following table:

Timeframe for Reporting Required Events:

**Deaths**	
Any reportable death while on study or within 30 days of study	Immediately, within 24 hours Lee Ratner, M.D., Ph.D. & IRB
Any reportable death while off study	Immediately, within 24 hours Lee Ratner, M.D., Ph.D. & IRB
**Adverse Events/Unanticipated Problems**	
Any ***reportable*** adverse events as described above in Section 9 (other than death)	Immediately, within 24 hours<br>- Lee Ratner, M.D., Ph.D.<br>- Within 10 working days IRB
All adverse events regardless of grade and attribution should be submitted cumulatively	Include in DSM report
**Non-Compliance and Serious Non-Compliance**	
All noncompliance and serious noncompliance as described in Sections 9.4 & 9.5	Immediately, within 24 hours<br>-Lee Ratner, M.D., Ph.D.<br>-Within 10 working day IRB

ADVERSE EVENT TELEPHONE CONTACT:

Protocol Chair
Lee Ratner, M.D., Ph.D.
Phone: 314-362-8836
Fax: 314-747-2120

9.2 Adverse Events (AEs)

Definition: any unfavorable medical occurrence in a human subject including any abnormal sign, symptom, or disease.

Grading: the descriptions and grading scales found in the revised NCI Common Terminology Criteria for Adverse Events (CTCAE) version 3.0 will be utilized for all toxicity reporting. A copy of the CTCAE version 3.0 can be downloaded from the CTEP web site:
(http://ctep.cancer.gov/protocolDevelopment/electronic_applications/docs/ctcaev3.pdf).

(http://www.hhs.gov/ohrp/policy/AdvEvntGuid.htm).

Attribution (relatedness), Expectedness, and Seriousness: The definitions for the terms listed that should be used are those provided by the Department of Health and Human Services' Office for Human Research Protections (OHRP). A copy of this guidance can be found on OHRP's website: (http://www.hhs.gov/ohrp/policy/AdvEvntGuid.htm).

9.3 Unanticipated Problems

Definition:

- Unexpected (in terms of nature, severity, or frequency) given (a) the research procedures that are described in the protocol-related documents, such as the IRB-approved research protocol and informed consent document; and (b) the characteristics of the subject population being studied;

- Related or possibly related to participation in the research (in this guidance document, possibly related means there is a reasonable possibility that the incident, experience, or outcome may have been caused by the procedures involved in the research); and

- Suggest that the research places participants or others at a greater risk of harm (including physical, psychological, economic, or social harm) than was previously known or recognized.

9.4 Noncompliance

Definition: failure to follow any applicable regulation or institutional policies that govern human subjects research or failure to follow the determinations of the IRB. Noncompliance may occur due to lack of knowledge or due to deliberate choice to ignore regulations, institutional policies, or determinations of the IRB.

9.5 Serious Noncompliance

Definition: noncompliance that materially increases risks, that result in substantial harm to subjects or others, or that materially, compromises the rights or welfare of participants.

9.6 Reporting to the Human Research Protection Office (HRPO) and the Quality Assurance and Safety Monitoring Committee (QASMC) at Washington University:

The PI is required to promptly notify the IRB of the following events:

- Any unanticipated problems involving risks to participants or others which occur at WU, any BJH or SLCH institution, or that impacts participants or the conduct of the study.
- Noncompliance with federal regulations or the requirements or determinations of the IRB.
- Receipt of new information that may impact the willingness of participants to participate or continue participation in the research study

These events must be reported to the IRB within **10 working days** of the occurrence of the event or notification to the PI of the event. The death of a research participant that qualifies as a reportable event should be reported within **1 working day** of the occurrence of the event or notification to the PI of the event.

9.7 Protocol Exceptions

Definition: A planned deviation from the approved protocol that are *under the research team's control.* Exceptions apply only to a single participant or a singular situation.

Local IRB pre-approval of all protocol exceptions must be obtained prior to the event.

For secondary sites, the Principal Investigator and Protocol Chair must be contacted and approved by both the Principal Investigator and the Protocol Chair if an exception is being sought. For secondary sites, the Washington University PI will issue approval of the exception, but it must also be submitted to the local IRB with documentation of approval forwarded to Washington University prior to the event. HRPO approval is not required for protocol exceptions occurring at secondary sites.

9.8 Reporting of AE Information Following Study Completion

All SAEs must be collected which occur within 30 days of discontinuation of dosing or completion of the patient's participation in the study if the last scheduled visit occurs at a later time

9.9 Reporting of Adverse Events at Participating Institutions

9.9.1 To the Washington University Research Patient Coordinator

All reportable events should be submitted to the Washington University Research Patient Coordinator according to the guidelines/timeframes described in Section 9.1. The Washington University Research Patient Coordinator will be responsible for reporting all events received to the HRPO promptly upon receipt disseminating this information to the participating sites.

Procedures for Adverse Event and Serious Adverse Event Reporting

Dr. Ratner must report all serious adverse events (SAE) regardless of relationship with any study drug or expectedness to Millennium within 24 hours. All sub-investigators must report all SAEs to Dr. Ratner so that he can meet his foregoing reporting obligations to Millennium.

Dr. Ratner must also provide Millennium with a copy of all communications related to the Study or Drug with the applicable regulatory authority, including, but not limited to, telephone conversation logs, within 24 hours of that communication. **The sponsor-investigator should fax the SAE form within 24 hours after becoming aware of the event.**

Millennium Pharmacovigilance
SAE and Pregnancy Reporting Contact Information: North America
PPD, Inc.
Safety and Medical Management, US
Fax: +1 888-488-9697
Hotline number (available 24/7): 1-800-201-8725

For both serious and non-serious adverse events, the investigator or sub-investigator must determine both the intensity of the event and the relationship of the event to drug administration.

Relationship to drug administration will be determined by Dr. Ratner or sub-investigator responding yes or no to the question: Is there a reasonable possibility that the adverse event is associated with the drug?

Intensity for each adverse event, including any lab abnormality, will be determined by using the NCI CTCAE, version 3.0, as a guideline, wherever possible. The criteria are available online at (http://ctep.cancer.gov/protocolDevelopment/electronic_applications/docs/ctcaev3.pdf).

9.10 Procedures for Reporting Drug Exposure During Pregnancy and Birth Events

If a woman becomes pregnant or suspects that she is pregnant while participating in this study, she must inform the investigator immediately and must permanently discontinue study drug(s). All pregnancies and suspected pregnancies must be reported to Millennium Pharmacovigilance (or designee; see Section 9.9 for contact information) immediately. The pregnancy must be followed for the final pregnancy outcome (ie, delivery, still birth, miscarriage) and Millennium Pharmacovigilance will request this information from the investigator.

If a female partner of a male patient becomes pregnant during the male patient's participation in this study, this must be reported to Millennium Pharmacovigilance (or designee) immediately (see Section 9.9 for contact information). Every effort should be made to follow the pregnancy for the final pregnancy outcome.

9.11 Monitoring of Adverse Events and Period of Observation

Adverse events, both serious and non-serious, and deaths that occur during the patient's study participation will be recorded in the source documents. All SAEs should be monitored until they are resolved or are clearly determined to be due to a patient's stable or chronic condition or intercurrent illness(es).

9.12 Reporting Requirement for Secondary Sites

The research team at each secondary site is required to promptly notify the Washington University PI and research coordinator of all reportable events (as described in Section 9.6) within **1 working day** of the occurrence of the event or notification of the secondary site's PI of the event. This notification may take place via email if there is not yet enough information for a formal written report (using FDA MedWatch 3500A form).
A formal written report must be sent to the Washington University PI and research coordinator within **10 working days** of the occurrence of the event or notification of the secondary site's PI of the event. The death of a research participant that qualifies as a reportable event should be reported within **1 working day** of the occurrence of the event or notification of the secondary site's PI of the event.

The research team at a secondary site is responsible for following its site's guidelines for reporting applicable events to its site's IRB according to its own institutional guidelines.

9.13 Reporting to Secondary Sites

The Washington University PI (or designee) will notify the research team at each secondary site of all reportable events that have occurred at other sites (as described in Section 9.6) within **10 working days** of the occurrence of the event or notification of the PI of the event. This includes events that take place both at Washington University and at other secondary sites, if applicable.

9.14 Multicenter Regulatory Requirements

Washington University requires that the each participating site must send their informed consent document to be reviewed and approved by the Principal Investigator/Study Chair or designee prior to IRB/IEC submission.

Each participating institution must have the following documents on file at Washington University prior to first subject enrollment:

- Documentation of IRB approval in the form of a letter or other official document from the participating institution's IRB. This documentation must show which version of the protocol was approved by the IRB.
- Documentation of IRB approved informed consent and protocol. Consent document must include a statement that data will be shared with Washington University, the Quality Assurance and Safety Monitoring Committee (QASMC), the DSMC, and the Washington University study team.
- Documentation of FWA, form, 1572, signed and dated CVs of all principal investigators.
- Documentation of training in protection of human subjects by all investigators.
- Protocol signature page signed and dated by the investigator at each participating site.

The Principal Investigator is responsible for disseminating to the participating sites all study updates, amendments, reportable adverse events, etc. There will be one current version of the protocol document at any given time and each participating institution will utilize that document. Protocol/consent modifications and IB updates will be forwarded electronically to the secondary sites within 2 weeks of obtaining Washington University IRB approval with acknowledgement of receipt requested. Secondary sites are to submit protocol/consent/IB modifications to their local IRBs within 4 weeks of receipt, and confirmation of submission must be forwarded to the appropriate contact person on the Washington University study team at the time of submission. Upon the secondary sites obtaining local IRB approval, documentation of such shall be sent to the Washington University study team within 2 weeks of receipt of approval.

Documentation of participating sites' IRB approval of annual continuing reviews, protocol amendments or revisions, all SAE reports, and all protocol violations/deviations/exceptions must be kept on file at Washington University.

9.15 Regular Conference Call Participation

The investigator or a designee from each institution must participate in a regular conference call to update and inform regarding the progress of the trial and to discuss toxicity.

10.0 DATA SUBMISSION SCHEDULE

Case Report Form (paper forms)	Submission Schedule
Original Consent Form	*Prior to registration*
Registration Form<br>*Eligibility Checklist*<br>*Baseline/Pretreatment Examination*<br>*Baseline Laboratory Studies*<br>*Concomitant Medications*<br>*Pathology/Bone Marrow Aspirate/Biopsy & Lumbar Puncture*<br>*Correlative Studies (Viral Studies)*	*Prior to starting treatment*
Treatment Record	*At completion of therapy, or q cycle*
During Treatment & Post Therapy Examination<br>*During Treatment & Post Therapy Laboratory Studies*<br>*Concomitant Medications*<br>*Tumor Measurement Form*<br>*Pathology/Bone Marrow Aspirate/Biopsy & Lumbar Puncture*<br>*Correlative Studies (Viral Studies)*	*At completion of therapy, or q cycle*
Early Discontinuation of Therapy	*At time of discontinuation of therapy*
Survival /Follow up	*Per protocol or at time of death*
Adverse Event Assessment Form<br>*MedWatch Form 3500A*	*See Section 9.1 for reporting requirements*

Any queries generated by Washington University must be responded to within 28 days of receipt by the participating site. The Washington University research team will conduct a regular review of data status at all secondary sites, with appropriate corrective action to be requested as needed.

10.1 Data Submission Guidelines

Completed case report forms and appropriate source documentation must be faxed to the Washington University Research Patient Coordinator according to the schedule listed in this section. Please save any verification notice of fax transmission.

11.0 EVALUATION OF RESPONSE

All patients will be evaluated for clinical response by physical examination following each chemotherapy cycle and by imaging studies at the conclusion of the second cycle of chemotherapy and after the fourth cycle, if given, and after the sixth cycle if given. Complete re-staging will also be performed every 4 months during the first year of follow-up after completion of therapy.

11.1 Response Assessment

Response is assessed on the basis of clinical, radiologic, and pathologic (i.e. bone marrow) criteria.

1. PET/CT scans remain the standard for evaluation of nodal disease. Thoracic, abdominal, and pelvic CT scans will be performed for staging even if those areas were not initially involved because of the unpredictable pattern of recurrence in NHL.
2. A bone marrow aspirate and biopsy should be performed to confirm a CR if they were initially positive or if it is clinically indicated by new abnormalities in the peripheral blood counts or blood smear unless peripheral blood involvement is unequivocally demonstrated by flow cytometry studies

11.2 Definition of Response

The response definitions used for this study are the 2007 Cheson criteria. A major distinction in the 2007 criteria is that PET/Gallium studies are used to facilitate the distinction between persistent tumor and scar/fibrosis.[1]

Complete Response (CR):

1. Complete disappearance of all detectable clinical evidence of disease and disease-related symptoms if present before therapy.

2a. Typically FDG-avid lymphoma: in patients with no pretreatment PET scan or when the PET scan was positive before therapy, a post-treatment residual mass of any size is permitted as long as it is PET negative.

2b. Variably FDG-avid lymphomas/FDG avidity unknown: in patients without a pretreatment PET scan, or if a pretreatment PET scan was negative, all lymph nodes and nodal masses must have regressed on CT to normal size (1.5 cm in their greatest transverse diameter for nodes > 1.5 cm before therapy). Previously involved nodes that were 1.1 to 1.5 cm in their long axis and more than 1.0 cm in their short axis before treatment must have decreased to 1.0 cm in their short axis after treatment.

3. The spleen and/or liver, if considered enlarged before therapy on the basis of a physical examination or CT scan, should not be palpable on physical examination and should be considered normal size by imaging studies, and nodules related to lymphoma should disappear. However, determination of splenic involvement is

not always reliable because a spleen considered normal in size may still contain lymphoma, whereas an enlarged spleen may reflect variations in anatomy, blood volume, the use of hematopoietic growth factors, or causes other than lymphoma.

4. If the bone marrow was involved by lymphoma before treatment, the infiltrate must have cleared on repeat bone marrow biopsy. The biopsy sample on which this determination is made must be adequate (with a goal of > 20 mm unilateral core). If the sample is indeterminate by morphology, it should be negative by immunohistochemistry. A sample that is negative by immunohistochemistry but that demonstrates a small population of clonal lymphocytes by flow cytometry will be considered a CR until data become available demonstrating a clear difference in patient outcome.
5. If the CNS was involved by lymphoma before treatment, the CSF cytology must be negative and any masses resolved.
6. For patients with acute (leukemic) form of ATLL malignant cells must be eliminated from peripheral blood based on morphologic and flow cytometric studies.

Partial Response (PR) requires all of the following:

1. At least a 50% decrease in sum of the product of the diameters (SPD) of up to six of the largest dominant nodes or nodal masses. These nodes or masses should be selected according to all of the following: they should be clearly measurable in at least two perpendicular dimensions; if possible they should be from disparate regions of the body; and they should include mediastinal and retroperitoneal areas of disease whenever these sites are involved.
2. No increase should be observed in the size of other nodes, liver, or spleen.
3. Splenic and hepatic nodules must regress by 50% in their SPD or, for single nodules, in the greatest transverse diameter.
4. With the exception of splenic and hepatic nodules, involvement of other organs is usually assessable and no measurable disease should be present.
5. Bone marrow assessment is irrelevant for determination of a PR if the sample was positive before treatment. However, if positive, the cell type should be specified (e.g., large-cell lymphoma or small neoplastic B cells). Patients who achieve a CR by the above criteria, but who have persistent morphologic bone marrow involvement will be considered partial responders. When the bone marrow was involved before therapy and a clinical CR was achieved, but with no bone marrow assessment after treatment, patients should be considered partial responders.
6. No new sites of disease should be observed.
7. Typically FDG-avid lymphoma: for patients with no pretreatment PET scan or if the PET scan was positive before therapy, the post-treatment PET should be positive in at least one previously involved site.
8. Variably FDG-avid lymphomas/FDG-avidity unknown: for patients without a pretreatment PET scan, or if a pretreatment PET scan was negative, CT criteria should be used.

9. For patients with acute (leukemic) form of ATLL a ≥ 50% from CD4+ cell in the peripheral blood and/or bone marrow.

<u>Stable disease (SD)</u> is defined as the following:

A patient is considered to have SD when he or she fails to attain the criteria needed for a CR or PR, but does not fulfill those for progressive disease (see Relapsed Disease [after CR]/Progressive Disease [after PR, SD]) below.

Typically FGD-avid lymphomas: the PET should be positive at prior sites of disease with no new areas of involvement on the post-treatment CT or PET.

Variably FDG-avid lymphomas/FDG-avidity unknown: for patients without a pretreatment PET scan or if the pretreatment PET was negative, there must be no change in the size of the previous lesions on the post-treatment CT scan.

<u>Relapsed Disease</u> (after CR)/Progressive Disease (after PR, SD):

Lymph nodes should be considered abnormal if the long axis is more than 1.5 cm regardless of the short axis. If a lymph node has a long axis of 1.1 to 1.5 cm, it should only be considered abnormal if its short axis is more than 1.0. Lymph nodes 1.0 x 1.0 cm will not be considered as abnormal for relapse or progressive disease.

Appearance of any new lesion more than 1.5 cm in any axis during or at the end of therapy, even if other lesions are decreasing in size. Increased FDG uptake in a previously unaffected site should only be considered relapsed or progressive disease after confirmation with other modalities. In patients with no prior history of pulmonary lymphoma, new lung nodules identified by CT are mostly benign. Thus, a therapeutic decision should not be made solely on the basis of the PET without histologic confirmation.

At least a 50% increase from nadir in the SPD of any previously involved nodes, or in a single involved node, or the size of other lesions (e.g., splenic or hepatic nodules). To be considered progressive disease, a lymph node with a diameter of the short axis of less than 1.0 cm must increase by 50% and to a size of 1.5 x 1.5 cm or more than 1.5 cm in the long axis.

At least a 50% increase in the longest diameter of any single previously identified node more than 1 cm in its short axis.

Lesions should be PET positive if observed in a typical FDG-avid lymphoma or the lesion was PET positive before therapy unless the lesion is too small to be detected with current PET systems (< 1.5 cm in its long axis by CT).

Measurable extranodal disease should be assessed in a manner similar to that for nodal disease. For these recommendations, the spleen is considered nodal disease. Disease

that is only assessable (e.g., pleural effusions, bone lesions) will be recorded as present or absent only, unless, while an abnormality is still noted by imaging studies or physical examination, it is found to be histologically negative.

A 50% increase in number of malignant cells compared to baseline in patients who have primarily bone marrow and/or leukemic disease.

Time To Response (TTR) is defined as time from the first dose of chemotherapy until documentation of first response.

Time To Progression (TTP) is defined as time from initiation of chemotherapy to documentation of first progression.

Response Duration is defined as the time from first documentation of response to documentation of first progression.

12.0 DATA AND SAFETY MONITORING

12.1 Data Safety Monitoring Reports

Each investigator will review all patient data at least every 4 months and provide a report to the PI for preparation of the DSM Report. Participating institutions are required to send this report one month prior to the report being due to the QASM Committee (every 4 months after Washington University HRPO approval). Each report will include:

1. Protocol title, IRB protocol number, and activation date of the study
2. Number of patients enrolled to date in each group
3. Date of first and most recent patient enrollment
4. Summary of all adverse events regardless of grade and attribution for each group
5. A response evaluation for evaluable patients
6. Summary of any recent literature that may affect the ethics of the study

The study principal investigator and study coordinator will monitor for reportable adverse events on an ongoing basis. Once the principal investigator or study coordinator becomes aware of an adverse event, the AE will be reported to the HRPO and QASM Committee according to guidelines in section 9.0.

12.2 Data and Safety Monitoring Committee (DSMC) Requirements

DSMC Documentation should include: DSM report (as prepared by the research team), recommendation report from DSMC (prepared & signed by DSMC chair), current consent & AE summary.

12.3 DSMC Membership

A DSMC will consist of no fewer than 3 members including 2 clinical investigators and a biostatistician. The DSMC members are Ben Tan, M.D., medical oncologist, Nina Wagner-Johnston, M.D., medical oncologist and Feng Gao, M.D., Ph.D. biostatistician.

Like investigators, DSMC members are subject to the Washington University Medical School policies regarding standards of conduct. Individuals invited to serve on the DSMC will disclose any potential conflicts of interest to the trial principal investigator and/or appropriate university officials, in accordance with institution policies. Potential conflicts that develop during a trial or a member's tenure on a DSMC must also be disclosed.

12.4 DSMC Responsibilities

The DSMC must meet on a regular schedule (not less than three times a year) over the course of study (with additional meetings as needed) to:

- Review data (including blinded data) over the course of the trial relating to efficacy, recruitment, randomization, compliance, retention, protocol adherence, trials operating procedures, form completion, intervention effects, gender and minority inclusion and subject safety.
- Identify problems relating to safety over the course of the study. Inform study principal investigator via written report, who in turn will ensure that all clinical collaborative site principal investigators receive this report.
- Identify needs for additional data relevant to safety issues and request these data from the study investigators.
- Propose appropriate analyses and periodically review developing data on safety and endpoints.
- At each meeting, consider the rationale for continuation of the study, with respect to recruitment, progress of randomization, retention, protocol adherence and compliance, data management, safety issues, and outcome data, if relevant, and make a recommendation for or against continuation of the trial.
- Provide the principal investigator and QASM, and PRMC Chairs written reports following each DSMC meeting. The principal investigator will then forward the report to the HRPO.
- Provide advice on issues regarding data discrepancies found by the data auditing system or other sources. If the QASM Chair requests this advice, it should be provided by the DSMC in writing within one month of the date of the request.
- If there is more than one clinical site, the study principal investigator is responsible for sending the reports to individual site principal investigators, who in turn are required to distribute the report to their local IRBs, as detailed in the NIH "Guidance on Reporting Adverse Events to Institutional Review Commitees for NIH-Supported Multicenter Clinical Trials" (NIH Guide for Grants and Contracts, June 11, 1999).

12.5 DSMC Meetings

DSMC meeting coordination is the responsibility of the research team. Data must be provided to the DSMC members by the research team prior to the meeting. DSMC meetings will be divided into an open and closed session. First is an open session during which members of the clinical trial team may be present, at the request of the DSMC, to review the conduct of the trial and to answer questions from members of the DSMC. Issues discussed may include accrual, protocol compliance, and general toxicity. Outcome results must not be discussed during the open session. Following the open session, a closed session involving the DSMC, and study statistical staff will be held to allow the DSMC opportunity to discuss the general conduct of the trial and all outcome results, including toxicities and adverse events, develop recommendations, and take votes as necessary.

12.6 DSMC Recommendations

DSMC recommendations should be based on results for the trial being monitored as well as on data available to the DSMC from other studies. It is the responsibility of the research team to ensure that the DSMC is kept apprised of non-confidential results from other related studies that become available. It is the responsibility of the DSMC to determine the extent to which this information is relevant to its decisions related to the specific trial being monitored.

A written copy of DSMC recommendation(s) will be given to the research team and QASMC. If the DSMC recommends that a study be changed for patient safety or efficacy reasons, or that a study be closed early because of slow accrual, the research team must act to implement the change as expeditiously as possible. In the unlikely situation that the research team does not concur with the DSMC, then the QASMC chair must be informed of the reason for disagreement. The research team, DSMC Chair, and the QASMC Chair will be responsible for reaching a mutually acceptable decision about the study. Confidentiality must be maintained during these discussions. However, in some cases, relevant data may be shared with other selected trial investigators and/or QASMC members to seek advice to assist in reaching a mutually acceptable decision.

If a recommendation is made to change a trial for other than patient safety or efficacy reasons or for slow accrual, the DSMC will provide an adequate rationale for its decision.

13.0 AUDITING

13.1 Auditing Process

Since Washington University is the coordinating center, each site will be audited annually by Siteman Cancer Center personnel (QASMC Office) unless the outside institution has an auditing mechanism in place and can provide a report. The outside sites will be asked to send copies of all audit materials including source documentation.

The audit notification will be sent to the Washington University Research Patient Coordinator, who will obtain the audit materials from the participating institution.

Notification of an upcoming audit will be sent to the research team one month ahead of the audit. Once accrual numbers are confirmed, and approximately 30 days prior to the audit, a list of the cases selected (up to 10 for each site) for review will be sent to the research team. However, if during the audit the need arises to review cases not initially selected, the research team will be asked to provide the additional charts within two working days.

Additional Details Regarding the Auditing Policies and Procedures can be found at the following website:
http://www.siteman.wustl.edu/uploadedFiles/Research_Programs/Clinical_Research_Resources/Protocol_Review_and_Monitoring_Committee/QASMCQualityAssurcance.pdf

14.0 STATISTICAL CONSIDERATIONS

14.1 Study objectives and endpoints

The primary objective of this study is to determine the tolerability and efficacy (response rate) of dose adjusted bortezomib-EPOCH (DA bortezomib-EPOCH) chemotherapy combined with Raltegravir in patients with HTLV-1 associated leukemia/lymphoma (ATLL). The secondary objectives include:

- To determine the duration of response of DA bortezomib-EPOCH chemotherapy combined with Raltegravir for HTLV-1 associated leukemia/lymphoma (ATLL).
- To evaluate the effects of DA bortezomib-EPOCH chemotherapy combined with Raltegravir on HTLV-1 DNA and RNA load, HTLV-1 integrase gene sequence, and HTLV-1 integration sites. To determine if relapsed or progressive disease is a result of renewed virus replication.
- To evaluate the relation of NFκB gene expression profile on response to DA bortezomib-EPOCH chemotherapy combined with Raltegravir.

14.2 Sample Size Estimation and Accrual

A two-stage Simon's Optimal design will be adopted to allow early termination for treatment inefficacy. It is assumed that the level of response below which one considers the regimen insufficiently active for further pursuit is 10%, and that the level at which one would have considerable interest in future testing is 30%. To test the null hypothesis that the response rate is less than or equal to 10% versus the alternative hypothesis that it is at least 30% with a significance level of 10% and power of 80% will require 18 evaluable patients. Seven patients will be enrolled during the first stage. An interim analysis will be conducted after all 7 patients have completed chemotherapy. If at least 1 objective response is observed, the study will proceed to the second stage and an additional 11 patients will be enrolled into the study. Preliminary evidence of

efficacy will be concluded if at least 4 out of the 18 patients exhibit response. Assuming a 10% dropout rate, 20 subjects will be required to ensure at least 18 evaluable patients. It is estimated that this study will accrue patients at a rate of 10 patients per year.

14.3 Statistical Analysis Plan

Data analysis of the study will be descriptive in nature. Demographic and clinical characteristics of the sample, toxicity by grade, as well as tumor response, duration of response and length of follow-up will be summarized using descriptive statistics. Binomial proportions and their 95% confidence intervals will be used to estimate the response rates to therapy. The Kaplan-Meier method will be used to evaluate the response duration. Analyses of variance methods will be used to evaluate the effects of treatment and time on the viral load measurements, as well as measurement of viral transcripts. The incidence of toxicities will be estimated using the binomial proportion and its 95% confidence interval. A proportional hazards analysis with viral load measures as time dependent covariates will be used to evaluate the effects of these measures on duration of response.

14.4 Safety Monitoring Plan

To assess the tolerability of regimen, a sequential probability ratio test (SPRT) will be used to monitor the frequency of subjects fail to complete 2 cycles of chemo-antiviral therapy due to toxicity. We assume that an intolerance rate less than 5% is acceptable and that an intolerance rate of 25% or more would definitely be not of interest. A SPRT-based early stopping rule, with 80% power at 0.05 significance level, will be followed to alert the investigators against excessive intolerance. That is, the study will be suspended or modified if it observes 2 intolerance in the first 4 patients, or 3 in the first 12 patients, or if the 4th case is observed before the 20th patient has completed the study.

15.0 ETHICAL AND REGULATORY CONSIDERATIONS

15.1 Good Clinical Practice

The study will be conducted in accordance with the International Conference on Harmonisation (ICH) for Good Clinical Practice (GCP) and the appropriate regulatory requirement(s). The Investigator will be thoroughly familiar with the appropriate use of the drug as described in the protocol and Investigator’s Brochure. Essential clinical documents will be maintained to demonstrate the validity of the study and the integrity of the data collected. Master files should be established at the beginning of the study, maintained for the duration of the study and retained according to the appropriate regulations.

15.2 Ethical Considerations

The study will be conducted in accordance with ethical principles founded in the Declaration of Helsinki. The IRB/IEC will review all appropriate study documentation in order to safeguard the rights, safety and well-being of the subjects. The study will only be conducted at sites where IRB/IEC approval has been obtained. The protocol, packet insert, Investigator's Brochure, informed consent, advertisements (if applicable), written information given to the subjects, safety updates, annual progress reports, and any revisions to these documents will be provided to the IRB/IEC by the Investigator. Millennium requests that the primary site (Washington University) protocol and informed consent documents be reviewed by Millennium or designee prior to IRB/IEC submission. Informed consent documents must be reviewed by Dr. Ratner/Washington University designee prior to enrolling subjects.

15.3 Informed Consent

The principles of informed consent described in Food and Drug Administration (FDA) regulations (21 CFR part 50) must be followed. IRB approval of the protocol and the informed consent form must be given in writing.

The sponsor must receive a copy of the letter of approval from the IRB, which specifically approves the protocol and informed consent, before patient enrollment. The IRB must also approve any significant changes to the protocol and documentation of this approval must be sent to the sponsor. Records of all study review and approval documents must be kept on file by the investigator and are subject to FDA inspection during or after completion of the study. Adverse events must be reported to the IRB.

The IRB should receive notification of completion of the study and final report within three months of study completion and termination. The investigator will maintain and accrue and complete record of all submissions made to the IRB, including a list of all reports and documents submitted.

15.4 Women and Minorities

This is a study being conducted by the Siteman Cancer Center which is a NCI Comprehensive Cancer Center. Each participating site is required to assure that the participation of women and minority subjects reflects the percentage representation of these populations in their geographic region and, for, the United States as a whole. As such, it is expected that the representation of subjects on this trial will reflect the constitution of the respective populations.

15.5 Research Authorization

Each institution should insert the appropriate research authorization sections into the informed consent document.

15.6 Subject Confidentiality

In order to maintain subject privacy, all data capture records, drug accountability records, study reports and communications will identify the subject by the assigned subject number. The Investigator will grant monitor(s) and auditor(s) from Millennium or its designees, the NCI and regulatory authority(ies) (FDA) access to the subject's original medical records for verification of data gathered on the data capture records and to audit the data collection process. The subject's confidentiality will be maintained and will not be made publicly available to the extent permitted by the applicable laws and regulations.

15.7 Protocol Compliance

The Investigator will conduct the study in compliance with the protocol given approval/favorable opinion by the IRB/IEC and the appropriate regulatory authority(ies). Changes to the protocol will require approval from Millennium and written IRB/IEC approval/favorable opinion prior to implementation, except when the modification is needed to eliminate an immediate hazard(s) to subjects. The IRB/IEC may provide, if applicable regulatory authority(ies) permit, expedited review and approval/favorable opinion for minor change(s) in ongoing studies that have the approval /favorable opinion of the IRB/IEC. The Investigator will submit all protocol modifications to Millennium and the regulatory authority(ies) in accordance with the governing regulations.

Any departures from the protocol must be fully documented in the source documents.

15.8 On-site Audits

Regulatory authorities, the IRB/IEC and/or Millennium's clinical quality assurance group may request access to all source documents, data capture records, and other study documentation for on-site audit or inspection. Direct access to these documents must

be guaranteed by the investigator, who must provide support at all times for these activities.

15.9 Drug Accountability

Accountability for the drug at all study sites is the responsibility of the Principal Investigator. The Investigator will ensure that the drug is used only in accordance with this protocol. Drug accountability records indicating the drug's delivery date to the site (if applicable), inventory at the site (if applicable), use by each subject, and return to the drug manufacturer or disposal of the drug (if applicable and if approved by the drug manufacturer) will be maintained by the clinical site. Accountability records will include dates, quantities, lot numbers, expiration dates (if applicable), and subject numbers.

All material containing bortezomib will be treated and disposed of as hazardous waste in accordance with governing regulations.

15.10 Premature Closure of the Study

This study may be prematurely terminated, if in the opinion of the investigator or Millennium, there is sufficient reasonable cause. Written notification documenting the reason for study termination will be provided to the investigator or Millennium by the terminating party.

Circumstances that may warrant termination include, but are not limited to:

- Determination of unexpected, significant, or unacceptable risk to patients
- Failure to enter patients at an acceptable rate
- Insufficient adherence to protocol requirements
- Insufficient complete and/or evaluable data
- Plans to modify, suspend or discontinue the development of the drug

Should the study be closed prematurely, all study materials must be returned to Millennium.

15.11 Product Complaints

A product complaint is a verbal, written, or electronic expression which implies dissatisfaction regarding the identity, strength, purity, quality, or stability of a drug product. Individuals who identify a potential product complaint situation should immediately contact MedComm Solutions (see below) and report the event. Whenever possible, the associated product should be maintained in accordance with the label instructions pending further guidance from a Millennium quality representative.

A medication error is a preventable event that involves an identifiable patient and that leads to inappropriate medication use, which may result in patient harm. While overdoses and underdoses constitute medication errors, doses missed inadvertently by

a patient do not. Individuals who identify a potential medication error situation should immediately contact MedComm Solutions (see below) and report the event.

**For Product Complaints or Medication Errors, call MedComm Solutions at**<br><br>**1-510-740-1273 (international number)**<br><br>**1-866-835-2233 (for US sites)**

Product complaints and medication errors in and of themselves are not AEs. If a product complaint or medication error results in an SAE, an SAE form should be completed and sent to PPD (refer to Section 9.9).

15.12 Record Retention

The investigator will maintain all study records according to ICH-GCP and applicable regulatory requirement(s).

16.0 REFERENCES

1. Takatsuki, K., Adult T-cell leukemia. Int Med. 34:947-952, 1995
2. Gessain, A., Mahieux, R., de The, G., Genetic variability and molecular epidemiology of human and simian T cell leukemia/lymphoma virus type I. J AIDS 13: Suppl 1:S132-145, 1996.
3. Manns A., Hisada, M., La Grenade, L. Human T-lymphotropic virus type I infection. Lancet 353:1951-1958, 1999.
4. Franchini, G. Molecular mechanisms of human T cell leukemia/lymphotropic virus type 1 infection. Blood 86:3619-3639, 1995.
5. Hollsberg, P. Mechanisms of T-cell activation by human T-cell lymphotropic virus type I. Microb Molec Biol Rev 63:308-333, 1999.
6. Mesnard, J-M., Devaux, C. Multiple control levels of cell proliferation by human Tcell leukemia virus type 1 tax protein. Virol 257:277-284, 1999.
7. Shimoyama, M. Diagnostic criteria and classification of clinical subtypes of adult Tcell leukaemia-lymphoma: a report from the Lymphoma Study Group (1984-87). Br J. Haematol 79:428-437, 1991.
8. Nagai, M., Usukiu, K., Wataru, M., et al., Analyis of HTLV-I proviral load in 202 HAM/TSP pateints and 243 asymptomatic HTLV-I carriers: high proviral load strongly predisposes to HAM/TSP. J. NeuroVirology. 4:685-593, 1998.
9. Ohshima, K., Suzumiya, J., Izumo, S., et al., Detection of human T-lymphotropic virus type-I DNA and mRNA in the lymph nodes; using polymerase chain reaction in situ hybridization (PCR/ISH) and reverse transcription (RT-PCR/ISH). Int J. Cancer 66:18-23, 1996.
10. Yoshida, M., Seiki M., Yamaguchi, K., Takatsuki, K. Monoclonal integration of human T-cell leukemia provirus in all primary tumors of adult T-cell leukemia suggests causative role of human T-cell leukemia virus in the disease. Proc. Natl. Acad. Sci. U.S.A. 81:2534-2538 , 1984.
11. Wattel, E., Cavrois, M., Gessain, A., Wain-Hobson, S. Clonal expansion of infected cells: a way of life for HTLV-I. J AIDS 13:Supple1:S92-99, 1996.
12. Bazarbachi, E., El-Sabban, M. E., Nasr, R., et al., Arsenic trioxide and Interferon-Alpha-2a synergize to induce cell cycle arrest and apoptosis in human T-cell lymphotropic virus type I-transformed cells. Blood 93:278-283, 1999.
13. Lymphoma Study Group. Major prognostic factors of patients with adult T-cell leukemia-lymphoma: a cooperative study. Leuk Res 15:81-90, 1991.
14. Bunn, P. A., Schechter, G. P., Jaffe, E,., et al. Clinical course of retrovirus-associated adult T-cell lymphoma in the United States. N Engl. J. Med. 309:257-264, 1983
15. Lofters, W., Campbell, M., Gibbs, W. N., Cheson, B. D. 2'Deoxycoformycin therapy in adult T-cell leukemia/lymphoma. Cancer 60:2605-2608, 1987.
16. Taguchi, H., Kinoshita, K. I., Takatsuki, K., et al. An intensive chemotherapy of adult T-cell leukemia/lymphoma: CHOP followed by etoposide, vindesine, ranimustine, and mitoxantrone with granulocyte colony-stimulating factor support. J. AIDS 12:182-186, 1996.
17. Gill, P. S., Harrington, W., Kaplan, M. H., et al. Treatment of adult T-cell leukemialymphoma with a combination of Interferon Alpha-2a and zidovudine. N Engl. J Med 332: 1744-1748, 1995.

18. Hermine, O., Bouscary, D., Gessain, A., et al., Treatment of adult T-cell leukemialymphoma with zidovuine and Interferon Alpha-2 a. N Engl J. Med 332:
19. Dega, H., Chosidow, O, Charlotte, F., et al., Unsuccessful association of zidovudine and Interferon Alpha-2a for acute adult T-cell leukemia lymphoma. Dermatology 198:103-105, 1999.
20. Waldmann, T. A., Goldman, C. K., Bongiovanni, K. F., et al., Therapy of patients with human T-cell lymphotropic virus I-induced adult T-cell leukemia with anti-Tac, a monoclonal antibody to the receptor for interleukin-2. Blood 72:1805-1816, 1988.
21. Taylor, G. P., Hall, S. E., Navarrete, S., et al., Effect of lamivudine on human T-cell leukemia virus type 1 (HTLV-1) DNA copy number, T-cell phenotype, and anti-Tax cytotoxic T-cell frequency in patients with HTLV-1-associated myelopathy J. Virol. 1999 73: 10289-10295.
22. Kchour G., Makhoul N. J., Mahmoudi M., et al. Zidovudine and interferon-alpha treatment induces a high response rate and reduces HTLV-1 proviral load and VEGF plasma levels in patients with adult T-cell leukemia from North East Iran. Leuk. Lymphoma. 48:330-336, 2007.
23. Gutierrez, M., Chabner, B. A., Pearson, D., Steinberg, S. M., Jaffe, E. S., Cheson, B. D., Fojo, A., Wilson, W. H. Role of a Doxorubicin-Containing Regimen in Relapsed and Resistant Lymphomas: An 8-Year Follow-Up Study of EPOCH. J. Clin Oncol, 2000 18:3633-3642
24. Wilson WH, Bryant G, Bates S, et al: EPOCH chemotherapy: Toxicity and efficacy in relapsed and refractory non-Hodgkin's lymphoma. J Clin Oncol 11: 1573-1582, 1993
25. Little R. F., Pittaluga S. Grant N., et al: Highly effective treatment of acquired immunodeficiency syndrome-related lymphoma with dose-adjusted EPOCH: impact of antiretroviral therapy suspension and tumor biology. Blood 101: 4653-4639, 2003.
26. Ratner L, Harrington W, Feng X, Grant C, Jacobson S, Noy A, Sparano J, Lee J, Ambinder R, Campbell N, Lairmore M, for the AIDS Malignancy Consortium. Human T-cell leukemia virus reactivation with progression of adult T-cell leukemia-lymphoma. PLOS One 4:e4429, 2009.
27. Satou Y, Nosaka K, Koya Y, Yasunaga JI, Toyokuni S, Matsuoka M. Proteasome inhibitor, bortezomib, potently inhibits the growth of adult T-cell leukemia cells both in vivo and in vitro. Leukemia 18:1357-1363, 2004.
28. Mitra-Kaushik S, Harding JC, Hess JL, Ratner L. Effects of the proteasome inhibitor PS-341 on tumor growth in HTLV-1 Tax transgenic mice and Tax tumor transplants. Blood 104:802-809, 2004.
29. Tsukasaki K, Hermine O, Bazarbachi A, et al. Definition, prognostic factors, treatment, and response criteria of adult T-cell leukemia-lymphoma: a proposal from an international consensus meeting. J Clin Oncol. 27:453-9, 2009.
30. Adams J, Palombella VJ, Sausville EA, Johnson J, Destree A, Lazarus DD et al. Proteasome inhibitors: a novel class of potent and effective antitumor agents. Cancer Res 1999; 59 (11):2615-22.

31. Steiner P, Neumeier H, Lightcap E, Sadis S, Pien C, Pink M et al. Adaptation of Human Tumor Cell Lines to PS-341. AACR-NCI-EORTC International Conference, 2001; Miami Beach, FL. Millennium Pharmaceuticals, Inc., 75 Sidney Street, Cambridge, MA 02139. Abstract
32. Teicher BA, Ara G, Herbst R, Palombella VJ, Adams J. The proteasome inhibitor PS-341 in cancer therapy. Clin Cancer Res 1999; 5 (9):2638-45.
33. Cusack JC, Jr., Liu R, Houston M, Abendroth K, Elliott PJ, Adams J et al. Enhanced chemosensitivity to CPT-11 with proteasome inhibitor PS-341: implications for systemic nuclear factor-kappaB inhibition. Cancer Res 2001; 61 (9):3535-40.
34. LeBlanc R, Catley LP, Hideshima T, Lentzsch S, Mitsiades CS, Mitsiades N et al. Proteasome inhibitor PS-341 inhibits human myeloma cell growth in vivo and prolongs survival in a murine model. Cancer Res 2002; 62 (17):4996-5000.
35. Pink MM, Pien CS, Ona VO, Worland P, Adams J, Kauffman MG. PS-341 enhances chemotherapeutic effect in human xenograft models. Proc Am Assoc Cancer Res, 2002; San Francisco, CA. Abstract 787
36. McConkey DJ, Pettaway C, Elliott P, Adam J, Papandreou C, Herrmann JL et al. The proteasome as a new drug target in metastatic prostate cancer. 7th Annual Genitourinary Oncology Conference, 1999; Houston, TX. Abstract
37. Hideshima T, Richardson P, Chauhan D, Palombella VJ, Elliott PJ, Adams J et al. The proteasome inhibitor PS-341 inhibits growth, induces apoptosis, and overcomes drug resistance in human multiple myeloma cells. Cancer Res 2001; 61 (7):3071-6.
38. Lightcap ES, McCormack TA, Pien CS, Chau V, Adams J, Elliott PJ. Proteasome inhibition measurements: clinical application. Clin Chem 2000; 46 (5):673-83.
39. Orlowski RZ, Stinchcombe TE, Mitchell BS, Shea TC, Baldwin AS, Stahl S et al. Phase I Trial of the Proteasome Inhibitor PS-341 in Patients with Refractory Hematologic Malignancies. J Clin Oncol 2002; 20 (22):4420-7.
40. Jagannath S, Barlogie B, Berenson J et al. A Phase 2 study of two doses of bortezomib in relapsed or refractory myeloma. Br J Haematol 2004: 127:165-172
41. Richardson PG, Barlogie B, Berenson J et al. A Phase II study of bortezomib in Relapsed, Refractory Myeloma. N Engl J Med 2003; 348:2609-2617
42. Blade J, Samson D, Reece D, Apperley J, Bjorkstrand B, Gahrton G et al. Criteria for evaluating disease response and progression in patients with multiple myeloma treated by high-dose therapy and haemopoietic stem cell transplantation. Myeloma Subcommittee of the EBMT. European Group for Blood and Marrow Transplant. Br J Haematol 1998; 102 (5):1115-23.
43. Richardson PG, et al., VELCADE or High-Dose Dexamethasone for Relapsed Multiple Myeloma. N Engl J Med 2005; 352:2487-98.
44. Richardson et al, VELCADE Continues to Demonstrate Superior Efficacy Compared With High-Dose Dexamethasone in Relapsed Multiple Myeloma: Updated Results of the APEX Trial. Blood, 2005; 106: Abstract 2547.
45. VELCADE (bortezomib) [package insert]. Cambridge, MA: Millennium Pharmaceuticals, Inc.; 2003.

We would like to thank the Alvin J. Siteman Cancer Center at Washington University School of Medicine and Barnes-Jewish Hospital in St. Louis, Missouri, for the use of the Clinical Trials Core which provided Protocol Development service. The Siteman Cancer Center is supported in part by an NCI Cancer Center Support Grant #P30 CA91842.

Appendix I: Karnofsky Performance Status Scale

The following table presents the Karnofsky performance status scale[1]:

Grade	Description
**100**	Normal; no complaints; no evidence of disease
**90**	Able to carry on normal activity; minor signs or symptoms of disease
**80**	Normal activity with effort; some signs of symptoms of disease. Unable to work, able to live at home and care for most personal needs; a varying amount of assistance is needed
**70**	Cares for self; unable to carry on normal activity or to do active work
**60**	Requires occasional assistance but is able to care for most of his needs
**50**	Requires considerable assistance and frequent medical care. Unable to care for self; requires equivalent of institutional or hospital care; disease may
**40**	Disabled; requires special care and assistance
**30**	Severely disabled; hospitalization is indicated although death is not imminent
**20**	Very sick; hospitalization necessary; active supportive treatment is necessary
**10**	Moribund; fatal processes progressing rapidly
**0**	Dead

[1] Mor V, Laliberte L, Morris JN, Wiemann M. The Karnofsky Performance Status Scale: an examination of its reliability and validity in a research setting. *Cancer* 1984;53:2002-2007.

Appendix II: Ann Arbor Staging Criteria

STAGE DESCRIPTION

STAGE I Involvement of a single lymph node region (I) or of a single extralymphatic organ or site (IE)

STAGE II Involvement of two or more lymph node regions on the same side of the diagphragm (II), or localized involvement of extralymphatic organ or site and of one or more lymph node regions on the same side of the diagphragm (IIE)

STAGE III Involvement of lymph node regions on both sides of the diaphragm (III), which may also be accompanied by localized involvement of extralymphatic organ or site (IIIE) or by involvement of the spleen (IIIS), or both (IIISE)

STAGE IV Diffuse or disseminated involvement of one or more extralymphatic organs or tissues with or without associated lymph node enlargement

A Absence of systemic symptoms

B Presence of one or more general symptoms: (1) unexplained weight loss of more than 10% of the body weight in the 6 months before admission; (2) unexplained fever with temperature above 38C; (3) night sweats

Notes:

1. The lymphatic structures are defined as the lymph nodes (N), spleen (S), thymus, Waledeyer's ring, appendix, and Peyer's patches
2. The reasons for classifying the patient as stage IV is defined further by defining sites by symbols:

H- Liver L-Lung
M- Marrow O-Bone
P- Pleura D-Skin

3. Liver involvement is always considered Stage IV disease, as is bone marrow involvement away from a site of an involved lymph node.

Appendix III: Central Pathology Review

Diagnostic slides will be centrally reviewed by an expert panel of hematopathologists. Within 30 days of registration, diagnostic slides, a copy of the original pathology report, 10 unstained slides or the diagnostic paraffin block and forwarded to:

Immunopathology
Attention: Sharon Barouk
Starr 715
New York Presbyterian Hospital
Cornell Campus
525 East 68th Street
New York, NY 10021
Contact: Dr Wayne Tam
Phone: 212-746-2442
Fax: 212-746-8173
Monday thru Friday, 8:00 a.m. – 6:00 p.m. Eastern Time
Specify in the paperwork that the sample is from ATLL Study 09-1758 / 201108212 for Central Pathology

Records of Specimens: Specimens should be accompanied by the appropriate specimen procurement and shipping forms in CRF package. Copies of these forms should also be forwarded to: The Operations Center at Washington University.

Appendix IV: Study Calendar

	Baseline/ Pretreatment	Cycle 1				Cycle 2 thru 6				Post therapy Every 2 months Yr 1 (+-/ 7 days Every 3 months Yr 2 (+/- 7 days) Every 6 months, Yrs 3-5 (+/- 14 days)
Description		Day 1 (up to -4 days)	Day 4	Day 5	Day 6	Day 1 (up to -4 days)	Day 4	Day 5	Day 6	
Complete History	X	X####				X				X
Physical Exam	X	X####				X				X
FACT/GOG-Neurotoxicity Questionnaire	X	X####				X				X###
CBC, Diff, Platelets	X	Days 1, 8, 10, 12 & 15 up to -4 days of each cycle				Days 1, 8, 10 12 & 15 up to -4 days of each cycle				X
Serum Chemistries#	X	X				X				X
Urinalysis	X									
T-cell Subsets	X					Day 1 of each odd numbered cycle & conclusion of chemo				Every 4 months Yr 1 only
Correlative Studies (Viral Studies)	X	X 50 ml				50 ml on Day 1 of cycle 1, 30 ml on Day 1 of cycle 2 and the each subsequent odd numbered cycle				30 ml every 4 months Yr 1 only*
EKG	X									
ECHO or MUGA	X									
PET/CT**	X					Prior to Day 1 of each odd numbered cycle				Every 4 months Yr 1 only
Serum Pregnancy****	X									
Bone Marrow Aspirate and Biopsy##	X					Prior to day 1 of each odd numbered cycle				At first follow up visit if positive at baseline
Lumbar Puncture*****	X									
Bortezomib		X	X			X	X			
Etopside		X----------------------X				X-----------------------------X				
Vincristine		X----------------------X				X-----------------------------X				

Doxorubicin		X---------------------X				X----------------------------X				
Prednisone		X----------------------------------X				X--X				
Cyclophosphamide				X				X		
Raltegravir						X --------------------------------Thru Day 21				
Neulasta or G-CSF***					X				X	

*At time of relapse 50 ml of blood in yellow top tubes is requested for viral studies (including gene expression assays).

**CT scans alone are sufficient at centers that do not have PET or gallium scans, CT or MRI's may be performed up to Day-7 before next cycle.

*** Neulasta 6 mg SQ on days 6, 7, or 8 or G-CSF 5 ug/kg (rounded to nearest 300 or 480 mcg) SQ daily for 10 days starting on day 6 or until the absolute neutrophil account has recovered to >4000 cells/mm3

****Female patients of child bearing potential, This should be performed within 72 hrs of initiation of therapy.

***** Lumbar Puncture should be deferred until after completion of Cycle 1 therapy if patient has leukemia at presentation.

Serum chemistries should include phosphorus, uric acid, and LDH

Bone marrows need to be repeated only if positive at baseline unless peripheral blood involvement is unequivocally demonistrated by flow cytometry

###At the first follow-up visit only

Does not need to be repeated on cycle 1 day 1 if done within 4 days.

Appendix V: Body Surface Area and Creatinine Clearance Calculations

Body surface area (BSA) should be calculated using a standard nomogram that yields the following results in meters squared (m^2):

$$BSA = \sqrt{\frac{Ht(inches) \times Wt(lbs)}{3131}}$$

or

$$BSA = \sqrt{\frac{Ht(cm) \times Wt(kg)}{3600}}$$

Creatinine clearance (CrCl) can be calculated using the Cockroft-Gault equation as follows:

$$CrCl\ (ml/min) = \frac{(140\text{-age})\ (\text{actual wt in kg})}{72 \times \text{serum creatinine (mg/dl)}}$$

For females use 85% of calculated CrCl value.

Note: In markedly obese patients, the Cockroft-Gault formula will tend to overestimate the creatinine clearance. (Adipose tissue tends to contribute little creatinine requiring renal clearance.)

Appendix VI: New York Heart Association Classification of Cardiac Disease

The following table presents the NYHA classification of cardiac disease:

Class	Functional Capacity	Objective Assessment
I	Patients with cardiac disease but without resulting limitations of physical activity. Ordinary physical activity does not cause undue fatigue, palpitation, dyspnea, or anginal pain.	No objective evidence of cardiovascular disease.
II	Patients with cardiac disease resulting in slight limitation of physical activity. They are comfortable at rest. Ordinary physical activity results in fatigue, palpitation, dyspnea, or anginal pain.	Objective evidence of minimal cardiovascular disease.
III	Patients with cardiac disease resulting in marked limitation of physical activity. They are comfortable at rest. Less than ordinary activity causes fatigue, palpitation, dyspnea, or anginal pain.	Objective evidence of moderately severe cardiovascular disease.
IV	Patients with cardiac disease resulting in inability to carry on any physical activity without discomfort. Symptoms of heart failure or the anginal syndrome may be present even at rest. If any physical activity is undertaken, discomfort is increased.	Objective evidence of severe cardiovascular disease.

Appendix VII : FACT/GOG-Neurotoxicity Questionnaire, Version 4.0

FACT/GOG-Neurotoxicity Questionnaire, Version 4.0

By circling one (1) number per line, please indicate how true each statement has been for you during the past 7 days.

**ADDITIONAL CONCERNS**	**Not at all**	**A little bit**	**Some-what**	**Quite a bit**	**Very much**
I have numbness or tingling in my hands	0	1	2	3	4
I have numbness or tingling in my feet	0	1	2	3	4
I feel discomfort in my hands	0	1	2	3	4
I feel discomfort in my feet	0	1	2	3	4
I have joint pain or muscle cramps	0	1	2	3	4
I feel weak all over	0	1	2	3	4
I have trouble hearing	0	1	2	3	4
I get a ringing or buzzing in my ears	0	1	2	3	4
I have trouble buttoning buttons	0	1	2	3	4
I have trouble feeling the shape of small objects when they are in my hand	0	1	2	3	4
I have trouble walking…	0	1	2	3	4

Sources: Cella DF, Tulsky DS, Gray G, Sarafian B, Lloyd S, Linn E, et al. The functional assessment of cancer therapy (FACT) scale: development and validation of the general measure. *J Clin Oncol* 1993;11(3):570-79.

Appendix VIII: Blood for HTLV-1 DNA Proviral Load, RNA, HTLV-1 Integrase Gene Sequence, Integration Site Analysis, & NFKB Expression Profiling

COLLECTION

Collect five yellow top tubes (50 ml) at baseline or three yellow top tubes (30 ml) at other time points of whole blood on the days when HTLV-1 proviral load, RNA, HTLV-1 integrase gene sequence, integration site, and NFκB expression profiling are done (see Appendix IV: Study Calendar).

SHIPPING

To ship bloods, place three yellow top tubes (30 ml) into a canister of a STP-100 SAF-T-PAK shipper (VWR# 11217-163) wrapping each tube in bubble wrap and using the absorbent paper at the bottom of the canister. Each sample tube should be labeled using a sharpie pen with the following information:

- Protocol #:
- Patient #
- Date and time of collection
- Specimen type – "Whole Blood"
- Specimen purpose: Virologic assays

Place the lid on the canister and place it inside of the ambient SAF-T-PAK shipper indicating on packaging "Virologic Assays, Attention: Dr Ratner". Seal the ambient shipper with cellophane shipping tape. Label the ambient shipper with the "Infectious substance" diamond shaped label. On one side, in black marker write "INFECTIOUS SUBTANCE AFFECTING HUMANS (HTLV) UN2814, your name or name of responsible person, date of collection and 24 hour phone number of the person responsible for the package.

Specimen Shipment Specimens may be shipped MONDAY through THURSDAY. All specimens should be shipped by overnight express at room temperature to:

Lee Ratner MD PhD
MSB562 - Oncology
Washington University
4566 Scott Ave
St Louis, MO 63110
TEL: (314) 362-8836
FAX: (314) 747-2120
Lratner@dom.wustl.edu

Use FED-EX "Dangerous Goods" airway bills for shipping. FED-EX account for the shipment: 2071-7627-9. It is only to be used for billing shipment of specimens to the lab where the sample is processed and/or stored. Call FED-EX at1-800-

463-3339 and press 0. Ask for customer service "dangerous goods" department. A FED-EX representative will assist in the specific wording required on the airway bills for pick-up and delivery of "Dangerous Goods". Place the completed airway bill marked **“Priority Overnight”** and the typed “**Shippers Declaration for Dangerous Goods”** on top of the shipper box inside of a plastic FED-EX pouch.

*****PLEASE DOUBLE CHECK PACKAGING OF SHIPPER AND DO NOT DEVIATE FROM REQUESTED LABELING.**

Please Note: The shipper will be mailed back to the site.
The STP-100 SAF-T-PAK shipper (VWR Cat #11217-163) is a complete kit w/all trappings, bubble wrap, adsorbent paper, labels, everything (but to reuse the shipper, you will need new labels, wrap, etc). There is a refurbishment kit w/extra b- wrap, adsorbent material (STP 102) (VWR Cat #11217-166) enough for 15 mailings.

Record of Specimens. Specimens should be accompanied by a copy of the “Peripheral Blood Correlative Samples Submission Form” (pages 11-12 of CRF packet). Copies of these forms should also be forwarded to the Operations Center at Washington University.

<u>Methodology for Correlative Studies</u>

PBMCs will be isolated on Ficoll for the correlative studies.

<u>HTLV-1 DNA Proviral Load</u>
Genomic DNA will be extracted from PBMCs isolated from whole blood obtained at baseline, day 1 of cycle 2, 3, and 5, and every 4 months during the 1st year of followup and at relapse. The HTLV-1 DNA assay is performed with peripheral blood mononuclear cells (PBMCs), prepared in a BSL3 facility, measuring the number of copies of integrated or unintegrated viral genome using ABI PRISM 7700 sequence detector (Perkin Elmer/Applied Biosystems) with primers in pX (26). The assay was standardized by measuring the number of copies of β-actin DNA, and performed in triplicate. The amount of HTLV-1 proviral DNA was calculated as copy number of HTLV-1 per 100 PBMC = [(copy number of px)/(copy number of β-actin/2)]×100.

<u>HTLV-1 RNA Levels</u>
RNA will be extracted from PBMCs isolated from whole blood obtained at baseline, day 1 of cycle 2, 3, and 5, and every 4 months during the 1st year of followup and at relapse. The HTLV-1 RNA assay was performed with PBMCs. RNA extraction, complementary DNA (cDNA) synthesis, and real time PCR are performed with primers designed for amplification of HTLV-1 *tax* cDNA, and human housekeeping gene hypoxanthine ribosyl transferase (*hprt*) for internal calibration. Standard curves are generated using cDNA from MT-2 cells, and all assays done in duplicate, with correlation values of standards more than 99%.

The relative *tax* mRNA load was calculated by the following formula: HTLV-1 *tax* mRNA load = {(value of *tax*)/(value of *hprt*)}×10,000.

HTLV-1 Integrase Gene Sequence

Genomic DNA will be extracted from PBMCs isolated from whole blood obtained at baseline, day 1 of cycle 2, 3, and 5, and every 4 months during the 1st year of followup and at relapse. Extraction will be performed with the the QIAamp DNA Blood Mini Kit (Qiagen, Valencia, CA) and quantified using an Ultrospec 2000 UV/Visible Spectrophotometer. 50ng of DNA be PCR amplified using the following primers:

Fwd 5' TTCGCAGCCATACCAATCTACC 3' (ACH 4215 – 4236)

Rev 5' GGAGCCACTAATGTCGGGGTCG 3' (ACH 5234 – 5255)

Automated DNA sequence analysis is performed with Big Dye terminator V3.1 using the primers listed above.

HTLV-1 Integration Site Analysis

Genomic DNA will be extracted from PBMCs isolated from whole blood obtained at baseline, day 1 of cycle 2, 3, and 5, and every 4 months during the 1st year of followup and at relapse. Extraction will be performed with the the QIAamp DNA Blood Mini Kit (Qiagen, Valencia, CA) and quantified using an Ultrospec 2000 UV/Visible Spectrophotometer. 500ng of DNA will be digested with MseI and NheI restriction enzymes (New England BioLabs, Ipswich, MA). A double stranded linker (lower strand 5'PO4-TAGTCCCTTAAGCGGAG-NH2-C7-3'; upper strand 5'-GTAATACGACTCACTATAGGGCTCCGCTTAAGGGAC-3') was ligated to the digested DNA fragments using NEB T4 DNA Ligase, followed by a first round of PCR amplification (fwd primer 5'-GTAATACGACTCACTATAGGGC-3', rev primer 5'-GCCGCTACAGATCGAAAGTT-3', amp cycle 7x: 94°C for 30s, 72°C for 4min; 32x: 94°C for 30s, 51°C for 30s, 72°C for 4min; 1x: 72°C for 5min) and a nested PCR step (fwd primer: 5'-19bp 454 primer-5bp bar code -ACGACTGACTGCCGGCTTG-3', rev primer 5'-AGGGCTCCGCTTAAGGGAC-3', amp cycle 5x: 94°C for 30s, 72°C for 4min; 32x: 94°C for 30s, 58°C for 30s, 72°C for 4min; 1x: 72°C for 5min). The PCR product will be submitted to the Genome Sequencing Center at Washington University for analysis on the 454 Life Science Genome Sequencer FLX.

Authentic integration sites will be identified based on those 1) that have >95% identity to human genomic DNA sequence, 2) start immediately after the LTR sequence, minus 6 nucleotides of repeat sequences, and 3) contain terminal LTR sequences minus 2 terminal nucleotides removed during integration. If an integration site matches multiple genomic sites, the best match is chosen only if it has a Blast socre of at least 10 higher than the second-best match. The 1000 most frequent unique integration sites from each sample identified, and ordered from more to least frequent site, and the number of times each site is identified recorded.

NFkB Expression Profile

High purity RNA will be prepared with the RNAwiz kit (Ambion) from purified $5x10^6$ CD25+ cells obtained at baseline and relapse. cDNA synthesis is performed with Affymetrix GeneChip double stranded cDNA synthesis kit. Gene expression analysis is performed with 5 µg RNA on the Affymetrix Gene Chip SNP Array 6.0 at the Washington University Multiplexed Gene Analysis Core. Complementary DNA (cDNA) is synthesized using the Ambion WT Expression kit according to manufacturer's guidelines. Labeling and hydridization with Affymetrix gene chips are performed using the Affymetrix GeneChip WT terminal labeling and hybridization kit. An NFkB expression score will be generated based on the level of expression of targets of the classical or alternative or both pathways, as employed in our previous studies. We will compare these gene expression results with our previous results using Tax transgenic tumor cell line expressing siRNAs to components of the classical, alternative, or both pathways. We will also compare the gene expression results to those from studies of activated B cell diffuse large B cell lymphomas. We will also determine if genes within 2 kb of integration sites are overexpressed or underexpressed at baseline or relapse, and we will determine if there are differences at these time points.

Purification of CD25+ Lymphocytes from PBMCs

CD25+ cells will be purified by immunomagnetic beads from 25 ml of PBMCs obtained with baseline and relapse samples using the Miltenyi protocol. FACS analysis will be performed before and after immunomagnetic bead purification to assess the level of purity and yield. After isolation of PBMCs from blood by Ficoll, the cells will be resuspended in cold MACS buffer (PBS, 0.5% BSA, 2 mM EDTA).10^7 cells will be resuspended in 90ul buffer and 10ul of the CD25 microbeads, incubated at 4° for 15 minutes, washed with 1-2 ml buffer and resuspended in MACS buffer. The column in the magnetic field will be pre-rinsed with 500ul buffer. The cell suspension will be added and unlabeled cells collected that pass through. The column will be washed three times with buffer. After the column dries, the column is removed from the magnet and buffer added. Labeled cells are flushed out using the plunger into a collection tube. We will count the cells and store the cells in a viable manner for future testing. A small amount of the cells are used to stain with anti-CD25 to determine the purity of separation by FACS.
