# Supplementary material for: Dose-adjusted EPOCH chemotherapy with bortezomib and raltegravir for human T-cell leukemia virus-associated adult T-cell leukemia lymphoma
Source: Blood Cancer J. 2016 Mar 25;6(3):e408–. doi: 10.1038/bcj.2016.21 (PMC4817103; doi:10.1038/bcj.2016.21)
Supplement: Supplementary Information [file bcj201621x2.pdf]

Supplemental Methods

Proviral DNA Assay:

Genomic DNA, extracted from PBMCs, was isolated from whole blood obtained at baseline, and at the beginning of cycles 2, 3, and 5 of treatment, and the end of study. The HTLV-1 DNA assay was performed with peripheral blood mononuclear cells (PBMCs), prepared in a BSL3 facility, measuring the number of copies of integrated or unintegrated viral genome with a Biorad digital drop PCR assay. PCR was performed with *tax* primers that amplify a 154 bp region, and a FAM/MGB probe. Additionally, in a duplex PCR, a cellular housekeeping gene, ribonuclease P protein subunit P30 was amplified and detected with VIC/MGB probe. DNA was digested with BamHI, mixed with the primers and probes and Bio-Rad 2× Supermix, and then emulsified using a QX-200 droplet generator, and PCR performed in a 96 well plate, and analyzed on the QX200 droplet reader. QuantaSoftware version 1.3.2.0 was used to quantify the copies/μl. Thresholds are determined manually for each experiment, according to the negative controls, which include a no template control and DNA from a healthy volunteer. Droplet positivity was determined by fluorescence intensity; only droplets above a minimum amplitude threshold are counted as positive. The PVL was calculated as the percentage of infected cells Data normalization was accomplished by applying the log (base 2) transformation, calculating the mean and standard deviation (SD), and defining the lower (mean-2SD) and upper (mean+2SD) values of the expected range for each assay type. These values were used to convert the log transformed values to a percentage of the expected range for each assay type, by subtracting the lower range value for the assay first then dividing by the difference of the upper and lower values of the expected range for the assay. Linear relationship of the post-normalized values was assessed using Pearson correlation.

Viral RNA Assays:

RNA, extracted from PBMCs, was isolated from whole blood obtained at baseline, and at the beginning of cycles 2, 3, and 5 of treatment, and the end of study. The HTLV-1 RNA assay was performed with PBMCs. RNA extraction, complementary DNA (cDNA) synthesis, and then digital drop PCR was performed. The extracted RNA was converted to cDNA using High Capacity cDNA Reverse Transcription Kit (Applied Biosystems, Foster City, CA) according to the manufacturer’s instructions. The conversion was performed using a GeneAmp 9700 thermocycler (Applied Biosystems, Grand Island, NY) with the following paramters: 10 min at 25 °C, 120 min at 37 °C, 5 min at 85 °C, and a hold at 4 °C. HTLV-1 tax and HBZ primers and

FAM/MGB probes were designed for ddPCR using NCBI Primer Blast and Primer3Plus. Additionally, we amplified a housekeeping gene, Hypoxanthine-guanine phosphoribosyltransferase (HPRT), using a commercially available mix (Life Technologies, Frederick, MD). The final concentrations in the ddPCR reaction were 900 nM of each primer and 250 nM of each probe.

For digital droplet PCR, the cDNA was mixed with the HTLV-1 tax (or HBZ) and HPRT1 primers and probes and Bio-Rad 2x Supermix, which was then emulsified with droplet generator oil (Bio-Rad, Hercules, CA) using a QX-200 droplet generator according to the manufactuter's instructions. The droplets were transferred to a 96-well reaction plate (Eppendorf, Hauppage, NY) and heat-sealed with pierceable sealing foil sheets (Thermo Fisher Scientific, West Palm Beach, FL.). PCR amplification was performed using a GeneAmp 9700 thermocycler (Applied Biosystems, Grand Island, NY) with the following cycling parameters: 10 min at 95 °C, 40 cycles consisting of a 30-s denaturation at 94 °C and a 60-s extension at 59 °C, followed by 10 min at 98 °C and a hold at 12 °C. Following PCR amplification, the 96-well plate was transferred to a QX100 droplet reader (Bio-Rad, Hercules, CA). Each well was queried for fluorescence to determine the quantity of positive events. QuantaSoft software version # 1.7.4.0917 (Bio-Rad, Hercules, CA) was used to quantify the copies/µl of each queried target per well. Droplet positivity was determined by fluorescence intensity. Thresholds were determined manually for each experiment, according to negative controls, which included a no template control and cDNA from a HTLV-I seronegative healthy volunteer. All samples were run in duplicate and the gene expression was the average of the two measurements. The gene expression was normalized to the housekeeping gene expression using the following formula: Gene expression = ((quantity of HTLV-1 tax or HBZ) / (quantity of housekeeping gene))*100

Primers and Probe Sequences:

Gene Name	Forward Primer (5'-3')	Reverse Primer (5'-3')	Probe Sequence (5'-3')
Tax cDNA	ATCCCGTGGAGACTCCTCAA	CCAAACACGTAGACTGGGTATCC	6FAM CCCCGCCGATCCCAAA MGBNFQ
HBZ cDNA	AGAACGCGACTCAACCGG	TGACACAGGCAAGCATCGA	6FAM ATGGCGGCCTCAGGGCT MGBNFQ

RNA Seq

Total RNA was obtained from peripheral blood mononuclear cells (PBMCs) from 4 acute ATLL patients at baseline and the endpoint of the study using RNAeasy (Qiagen) and submitted to the Washington University Genome Technology Access Center (GTAC) for RNAseq Analysis. Reads per kilobase of transcript per million

mapped reads (RPKM) for all transcripts was determined and the average of protein coding transcripts was used for analysis. Transcripts elevated in both patients who failed to respond to treatment compared to both patients who responded to treatment were identified in baseline and endpoint samples.

Analysis of GSE33615 Microarray Data

Data from an ATLL gene expression microarray study [11] was downloaded from the Gene Expression Omnibus at the NCBI (GEO accession: GSE33615). In the original study, RNA was extracted from PBMCs isolated from patients with acute (n=26), chronic (n=20), lymphomatous (n=1), and smoldering (n=4) ATLL, and compared to RNA obtained from $CD4^+$ cells from 21 normal subjects. In this study, values were normalized to actin (ACTB) then represented as fold-Patient 10 (a smoldering ATLL sample with the lowest proviral load in the study). The 60-mer probe for Blk used in the Agilent-014850 Whole Human Genome Microarray (TCGCACGGTCATCCGGAGTACTAAGCCCCAGTAAGGTGTTCAGGACTGGTAAGCGACTGT) corresponds to chr8:11564349-11564408 in the GRCh38.p2 assembly, the 3'UTR of full-length protein coding Blk transcripts. The 60-mer Probe used for CD101 (AAGTAAGGTACGTGTCTCCAAAGTGTACTGGACCGAAAATGTGACTGAGCACAGAGAAGT) corresponds to chr 1:117025510-117025569 in the GRCh38.p2 assembly within an exon present in both full-length, protein-coding transcripts.

Illumina Sequencing

Genomic DNA from PBMCs of ATLL patients was extracted and fragmented by sonication to generate 200-300 bp fragments of genomic DNA. Viral DNA was captured with four long terminal repeat (two each at the 5' and 3' termini of the LTR) and nine integrase gene probes, spanning the entire gene, each biotin labeled and120 nucleotides in length. After the hybridization of probes to the target sequences, a pull down assay was performed with anti-biotin beads to select viral sequences. After washing to remove the excess material, the beads were removed and the pulled-down sequences were used to make the DNA libraries, with Illumina linkers and amplified using specific index primers for each patient's DNA sample. For the sequencing process, the ultra-high-throughput sequencing system, HiSeq 2500, was used. Thirty-five samples were mixed together and sequenced by paired-end reads of 250 nucleotides each. A third read was used to identify the index of

each DNA sample. Sequence data were demultiplexed and aligned to a reference fasta file comprising human GRCh37 and the HTLV-1 proviral DNA sequence, complete genome (GenBank: AB513134.1) using bwa mem (v0.7-10; "Li H. (2013) Aligning sequence reads, clone sequences and assembly contigs with BWA-MEM. arXiv:1303.3997v1 [q-bio.GN]."). PCR duplicates were marked using picard (v1.99; http://picard.sourceforge.net) and target coverage calculated using bedtools (v2.23.0; PMID: 20110278). Viral integration sites were detected using lumpy (v0.2.11; PMID: 24970577), to find breakpoints in which one side of the breakpoint mapped uniquely to the human genome and the other mapped to the HTLV-1 5' LTR. For sequence fragments (filtered for mapping quality>20) that aligned to the HTLV-1 integrase genome, counts of unique reads encoding each amino acid at each codon position were determined using a custom walker written using the Genome Analysis Toolkit (PMID: 20644199). Intra-sample divergence was calculated as the count of reads encoding a non-reference amino acid, averaged over all codon positions.

Table S1. Toxicity Table

Number of subjects with each of the follow adverse events are listed according to maximal grade during clinical trial participation.

	Grade 1	Grade 2	Grade 3	Grade 4	Grade 5
**Allergy**					
Sinusitis	1				
Rhinorrhea	1				
**Cardiovascular**					
Edema	2				
Hypertension	1				
Chills	1				
**Constitutional**					
Fatigue	5	1	1		
Fever-no infection	1	1			
Weight gain					
Weight loss		1			
Dry Skin	1				
**Dermatologic**					
Alopecia	1				
Rash		1			
Dry Eyes	1				
Nail Discoloration	1				
Pigment Changes	1				
**Gastrointestinal**					
Anorexia		2			
Constipation		2			
Dehydration	1				
Indigestion	2				
Dysgeusia	1				
Mucositis	3				
Nausea	2				
Vomiting	1		1		
GI Bleed/Ulcers		1			
Spontaneous bacterial peritonitis			1		
Abdominal distention			2		
Abdominal pain			1		
**Hematological**					
Hemoglobin	4	1	6		
Leukocytes (WBC)		1	5	2	
Lymphopenia			1		
Neutrophils (ANC)		1	1	5	
Platelets	2		2	4	
**Hepatic**					
Alkaline Phosphatase	3	1			
Bilirubin					
SGOT (AST)	3	1			
SGPT (ALT)	3	1			
**Infection**					
Infection without neutropenia	1		1		
Infection with neutropenia			1		
Omaya port infection			1		
IV port infection			1		

	Grade 1	Grade 2	Grade 3	Grade 4	Grade 5
Sepsis				1	1
Neutropenic fever			2	1	
Septic arthritis		1			
Vaginal infection		1			
**Metabolic/Laboratory**					
Albumin Low ( ) High ( )	2	2			
Calcium Low (x) High ( )		2			
Calcium Low ( ) High (x)	2	1			
Glucose Low (x) High ( )		1	1		
Glucose Low ( ) High (x)	1	1	2		
Magnesium Low ( ) High ( )	2		1		
Potassium Low (x) High ( )	3		1		
Sodium Low (x) High ( )	4				
Hypertriglyceridemia				1	
Sodium High	1				
Uric Acid	1				
Hypophosphatemia	2				
**Neurological**					
Confusion		1	1		
Headache	1		1		
Neuropathy (Type _Sensory_____)	5	4			
Seizure		1			
Encephalitis			1		
Fatigue	1				
Opthalmoplegia/laryngealn/aphasia		1			
Blurred vision	1				
**Pain**					
Abdominal			1		
Bone	2				
Back	1				
Headache	1				
Extremity pain	1				
**Pulmonary**					
Cough	2	1	1		
Dyspnea (SOB)			1		
Congestion	1				
**Renal/Genito-Urinary**					
Creatinine	1	1			

Patient A
Best Response: PR

Patient B
Best Response: PD

Patient C
Best Response: PR

Patient D
Best Response: PD

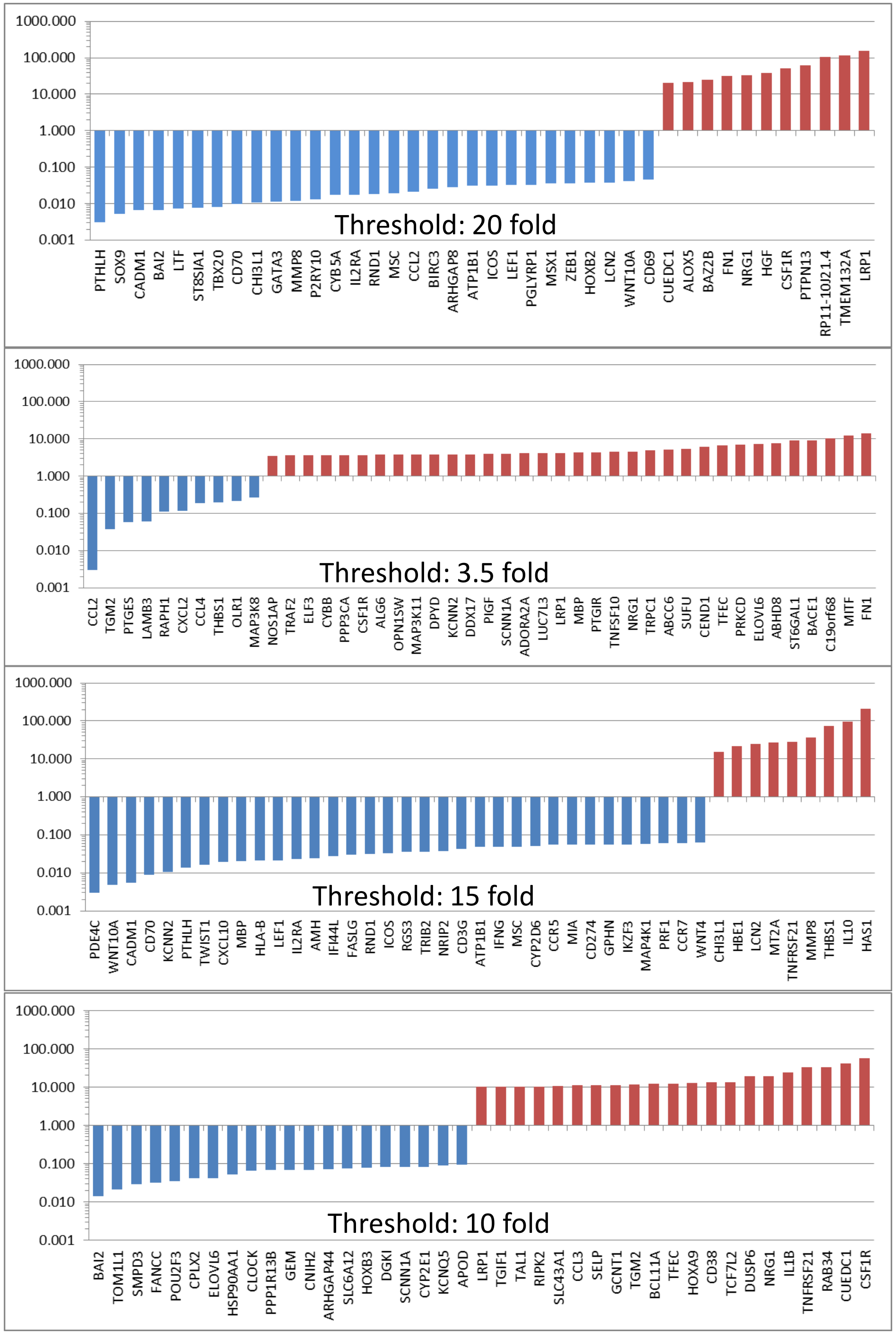

Fig S1. The effect of therapy on NFkB target genes.
RNAseq was performed on RNA obtained from PBMCs collected from 4 patients (A,B,C,D) with acute disease before and after treatment with Dose-Adjusted EPOCH Chemotherapy combined with Bortezomib and Raltegravir. The ratio of RPKM values for each transcript after therapy vs. before therapy are shown for transcripts of NFkB target genes. Independent thresholds for each patient were established to identify the 40 genes most affected by treatment in each patient. In patients that achieved a partial response (PR) to therapy (A and C), the most affected NFkB targets were more likely to be repressed after therapy than in patients B and D with progressive disease (PD).

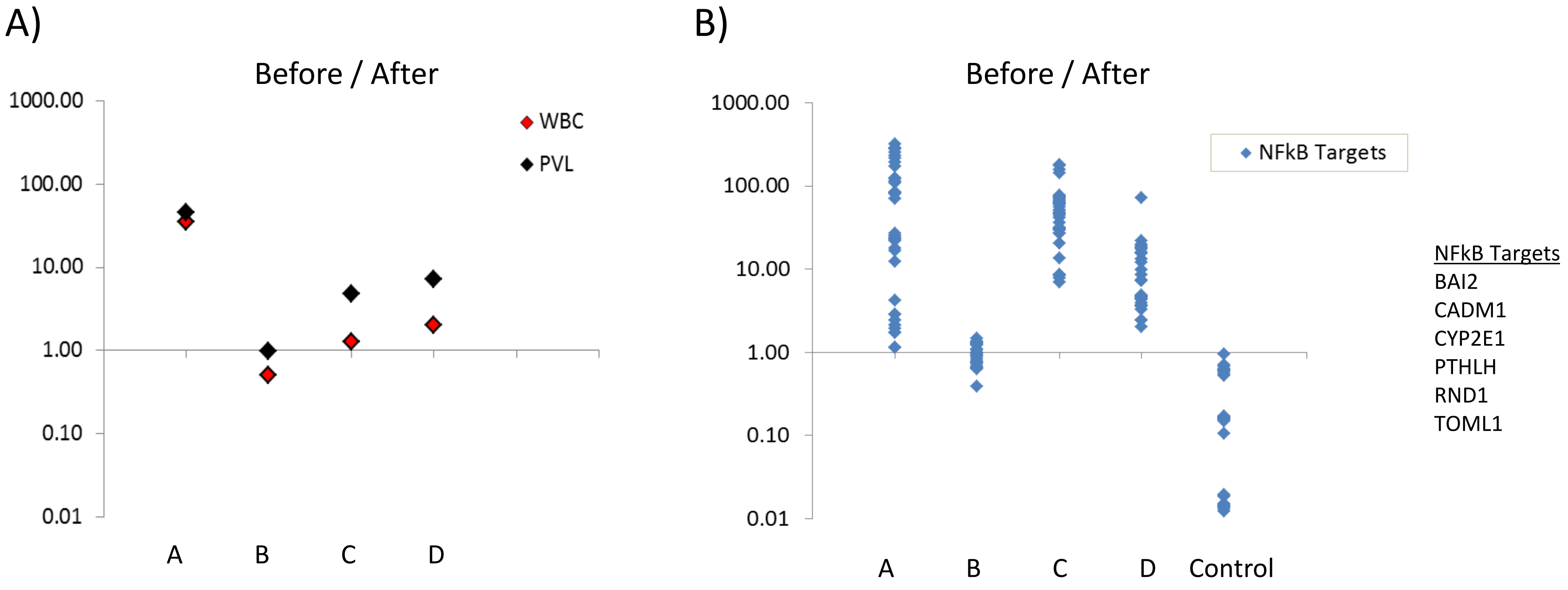

Fig S2. The effect of therapy on NFkB target genes

RNAseq was performed on RNA obtained from PBMCs collected from 4 patients (A,B,C,D) with acute disease before and after treatment with dose-adjusted EPOCH chemotherapy combined with Bortezomib and Raltegravir. The ratio of A) WBC and PVL and B) RPKM values for protein coding transcripts of 6 NFkB target genes before therapy vs. after therapy are shown. The control shown in B is the ratio of transcripts from the same NFkB target genes taken at presentation and relapse from a patient not in this study who did not receive Dose-Adjusted EPOCH Chemotherapy combined with Bortezomib and Raltegravir.

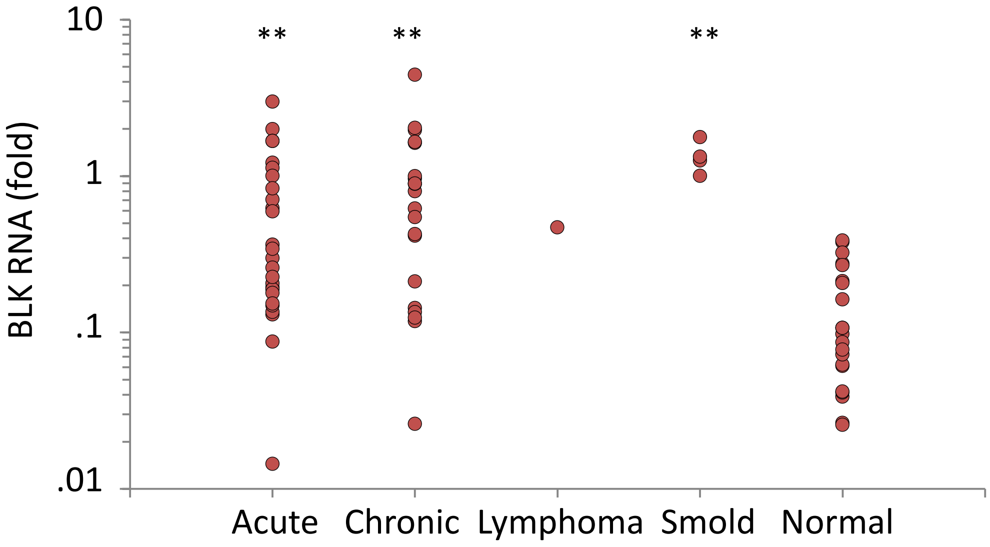

Figure S3. Blk expression is elevated in a subset of primary ATLL samples. Quantitation of Blk RNA obtained from an ATLL gene expression microarray study available in the Gene Expression Omnibus at the NCBI (GEO accession: GSE33615). Acute (n=26), Chronic (n=20), Lymphomatous (n=1), and Smoldering (n=4) primary ATLL samples, were compared to RNA obtained from CD4+ cells from normal subjects (n=21). Values were normalized to Actin (ACTB) then represented as fold Patient 10 (a smoldering ATLL sample with the lowest proviral load in the study).
** indicate p-value <0.01 (2-tailed T-Test)

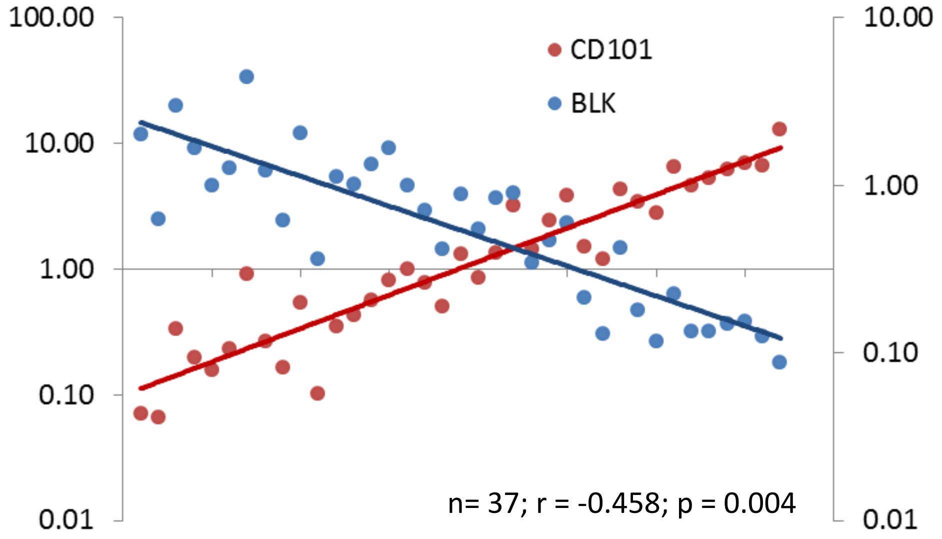

Figure S4. Blk expression inversely correlates with CD101 (IGSF2) in most ATLL cases.
Pearson's Correlation of 37 of 52 primary ATLL samples (GSE33615) comparing expression of BLK to CD101 results in an inverse correlation (r) of -0.458.
